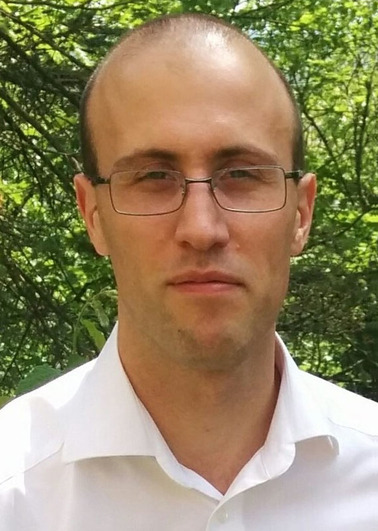# Biosensing with Fluorescent Carbon Nanotubes

**DOI:** 10.1002/anie.202112372

**Published:** 2022-03-01

**Authors:** Julia Ackermann, Justus T. Metternich, Svenja Herbertz, Sebastian Kruss

**Affiliations:** ^1^ Physical Chemistry Ruhr-University Bochum Universitätsstrasse 150 44801 Bochum Germany; ^2^ Biomedical Nanosensors Fraunhofer Institute for Microelectronic Circuits and Systems Finkenstrasse 61 47057 Duisburg Germany; ^3^ Department EBS University Duisburg-Essen Bismarckstrasse 81 47057 Duisburg Germany

**Keywords:** biosensors, carbon nanotubes, imaging, molecular recognition, near-infrared fluorescence

## Abstract

Biosensors are powerful tools for modern basic research and biomedical diagnostics. Their development requires substantial input from the chemical sciences. Sensors or probes with an optical readout, such as fluorescence, offer rapid, minimally invasive sensing of analytes with high spatial and temporal resolution. The near‐infrared (NIR) region is beneficial because of the reduced background and scattering of biological samples (tissue transparency window) in this range. In this context, single‐walled carbon nanotubes (SWCNTs) have emerged as versatile NIR fluorescent building blocks for biosensors. Here, we provide an overview of advances in SWCNT‐based NIR fluorescent molecular sensors. We focus on chemical design strategies for diverse analytes and summarize insights into the photophysics and molecular recognition. Furthermore, different application areas are discussed—from chemical imaging of cellular systems and diagnostics to in vivo applications and perspectives for the future.

## Graphical abstract

Optical biosensors are important tools for basic research and non‐invasive diagnostics. Carbon nanotubes are versatile near‐infrared fluorescent and non‐bleaching materials that can be chemically functionalized to detect a broad variety of biomolecules. This Review highlights chemical design strategies and provides a comprehensive overview of the recent developments in this dynamic field.

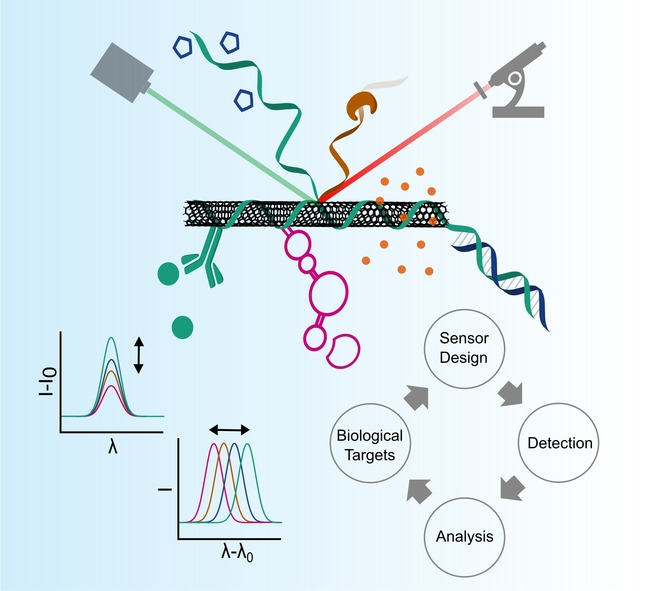

## 1 Introduction

Future challenges in medicine such as early disease detection, point‐of‐care diagnostics, and tailored therapies require novel methods of biosensing. Additionally, biosensors can provide insights into the complex dynamics of biological and chemical systems. Consequently, they are essential tools for both fundamental research and biomedicine. In particular, optical sensing approaches possess a great potential for contactless real‐time readouts that are required in biomedical research, as well as industrial healthcare and agriculture applications.[^1^, ^2^, ^3^] During the last decade, the field of biosensors based on nanomaterials has seen vast improvements.[^4^, ^5^] These materials include carbon‐based nanomaterials such as graphene, graphene quantum dots, and carbon nanotubes (CNTs).[^4^, ^6^, ^7^, ^8^] Here, single‐walled carbon nanotubes (SWCNTs) are of particular interest. Their optoelectronic properties are sensitive to the surrounding environment, which makes them suitable for highly selective biosensing.[^8^, ^9^, ^10^, ^11^, ^12^, ^13^, ^14^, ^15^, ^16^] When dispersed in aqueous solutions, SWCNTs fluoresce without bleaching in the near‐infrared region (NIR, around *λ*=870–2400  nm).[^17^, ^18^] This region of the electromagnetic spectrum is beneficial for detection and imaging as it offers an ultralow background and high penetration depths in biological tissues (tissue transparency window).[^1^, ^2^, ^9^, ^19^, ^20^, ^21^] Fluorescence methods using common visible fluorophores often suffer from high scattering, absorption, and autofluorescence, which limits the penetration depth and signal to noise ratios.^[1]^ Additionally, phototoxicity is increased by excitation of common fluorophores with visible (Vis) or ultraviolet (UV) light. Consequently, SWCNTs offer an advantage as they combine the biocompatibility and photostability required for optical sensing and imaging with emission in the NIR region.[^14^, ^22^, ^23^] Furthermore, the structural diversity of SWCNTs promises tunable emission wavelengths.[^10^, ^12^] SWCNTs are highly sensitive to environmental changes, which is the basis for molecular recognition and was pioneered by optical sensors for glucose detection and DNA polymorphism.[^24^, ^25^] Both covalent or noncovalent functionalization approaches play an essential role in tailoring molecular interactions close to the SWCNT surface.[^10^, ^14^, ^17^, ^23^, ^26^] By using such concepts, SWCNT‐based biosensors for many highly important biomolecules have been developed.

More recently, this allowed chemical signaling to be mapped in a completely new manner, for example, release patterns of neurotransmitters from cells with high spatial and temporal resolution, which provides unique insights into fundamental biological questions.[^27^, ^28^] Moreover, recent advances have been made in remote in vivo biosensing applications by the multimodal optical detection of several analytes. By combining multiple nanosensor elements and integrating them into functional arrays, analytes can be identified and distinguished on the basis of their characteristic image signatures.^[29]^ Such a combination of optical nanosensors could pave the way for the next generation of fast and reliable in situ diagnostics. In addition, these approaches provide completely new opportunities for standoff process controlling, for example, fabrication of antibodies or monitoring in food and agriculture industries (smart plant sensors).[^3^, ^19^, ^30^, ^31^, ^32^, ^33^]

In this Review we focus on optical biosensing with SWCNTs to give an update on this fast‐evolving field. We evaluate in detail the specificity, sensitivity, spatial resolution, and biocompatibility of different SWCNT‐based biosensors. This Review follows on from previous reviews,[^2^, ^10^, ^14^, ^23^, ^34^, ^35^] and discusses new chemical strategies developed in the last few years. SWCNTs can also serve as NIR labels. However, this is not discussed here and we refer to other excellent reviews.[^1^, ^2^]

In Section 2, the basic structural properties and photophysics of SWCNTs as well as functionalization strategies are described. To conclude this section, we touch on the most important aspects of SWCNT biocompatibility. Section 3 contains an overview of general chemical recognition strategies. We provide a detailed and up‐to‐date summary of all currently accessible biomolecular target groups, including reactive oxygen species (ROS), neurotransmitters, proteins, antibodies, lipids, and sugars. This overview is complemented with mechanistic insights into how these sensors work. Finally, we provide a perspective on the field (Sections 3 and 4) and discuss possible future directions. This includes novel biological topics such as plants, advanced chemical tools (defects), methods for improved (hyperspectral) imaging, novel screening approaches, and multiplexing.

## 2 Functionalization Concepts

Since the report of their structure, CNTs have attracted wide interest within the scientific community and beyond. Their remarkable mechanical, electrical, and photophysical properties have paved the way for applications in the fields of advanced materials, microelectronics, biosensing, imaging, drug delivery, and many more.[^8^, ^36^] Here, we will briefly describe the structure and photophysics of SWCNTs, followed by approaches to tailor their surface chemistry and biocompatibility.

### 2.1 SWCNT Structure and Photophysics

**Figure 1:**
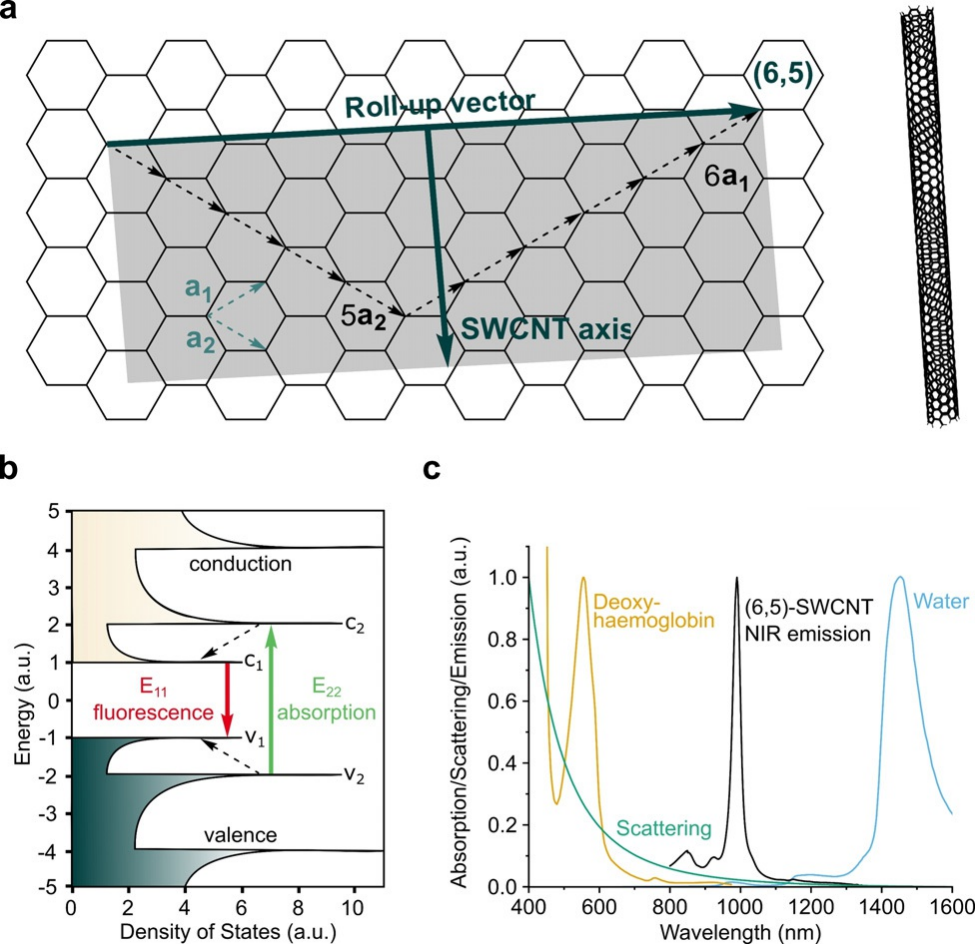
Structure and properties of single‐walled carbon nanotubes (SWCNTs). a) The structure of SWCNTs can be rationalized by rolling a sheet of graphene along its roll‐up vector, for example, *c*=6a_1_+5a_2_. b) The band gap structure gives rise to fluorescence emission in the near‐infrared (NIR) region. c) The E_11_ transition of SWCNTs^[49]^ overlaps with the tissue transparency window, thus offering the advantage of reduced light absorption,^[50]^ scattering (e.g. Rayleigh), and background fluorescence. Here, the emission spectrum of SWCNTs of (6,5)‐chirality is shown, but the emission wavelength for other chiralities span the whole NIR region.

CNTs can be conceptualized as rolled‐up cylinders of graphene.^[37]^ Their properties are determined by the exact sp^2^‐hybridized carbon lattice as well as by the number of cylinders that are stacked into each other.^[37]^ CNTs derived from a single graphene cylinder are called single‐walled carbon nanotubes (SWCNTs),^[38]^ whereas tubes consisting of multiple layers are called multiwalled carbon nanotubes (MWCNTs).^[7]^ SWCNTs are commonly labeled using the chiral index (*n*,*m*), where *n* and *m* are integers that describe the carbon lattice structure (Figure 1 a).[^37^, ^39^] In this notation, the SWCNT is conceptually rolled up along the vector c=na_1_+ma_2_ (a_1_ and a_2_ are the graphene lattice vectors). Consequently, the roll‐up vector also determines the diameter. For SWCNTs, the reported diameters range from 0.4  nm to 10  nm.[^8^, ^40^]

The roll‐up vector affects the density and energy of the electronic states of SWCNTs and consequently the optoelectronic properties are directly related to chirality. As a result, for *n*−*m*=0 (armchair configuration), SWCNTs are metallic, for *n*−*m*=3j (j$\;\in \mathbb{N}\setminus \left\{0\right\}$ ), semimetallic, and semiconducting for all other (*n*,*m*) chiralities.^[10]^ When SWCNTs are excited with light, an electron–hole pair (exciton) can be created and diffuses along the SWCNT axis.^[41]^ For semiconducting SWCNTs,^[18]^ the absorption of photons with energies corresponding to the visible spectrum of light typically leads to excitation to the second conducting band (Figure 1 b).[^9^, ^12^]

Fast decay (femtosecond time scale) to the first conduction band followed by radiative recombination, then causes fluorescent emission in the NIR region (>870 nm),[^18^, ^42^] a region that is particularly interesting for biological imaging (Figure 1 c). Quantum chemical considerations predict 4 singlet and 12 triplet excitonic states.[^43^, ^44^] However, only the transition from the singlet state is optically allowed.[^43^, ^44^] As the energy of this state is higher than the majority of other singlet and triplet states,[^43^, ^44^] a variety of dark exciton decay pathways exist.^[45]^ For (6,5)‐SWCNTs, the size of an exciton is approximately 2 nm.^[46]^ During their lifetimes they diffuse in the range of 100 nm along the SWCNT axis.[^47^, ^48^] As all carbon atoms are located on the surface of the SWCNT, excitons are affected by the nanotube corona (i.e. the organic phase around the SWCNTs). Consequently, the photophysics of SWCNTs are highly influenced by chemical processes around their surfaces. This renders them ideal building blocks and transducers for chemical and biological sensing.

### 2.2 Surface Functionalization

The extended π‐system makes SWCNTs hydrophobic and consequently they easily aggregate in solvents like water. Therefore, an important step in the preparation of SWCNT‐based sensors is their functionalization to isolate, solubilize and colloidally stabilize single SWCNTs. The functionalization also serves the purpose to a) interact (specifically) with other molecules and b) translate this interaction into a fluorescence change.

**Figure 2:**
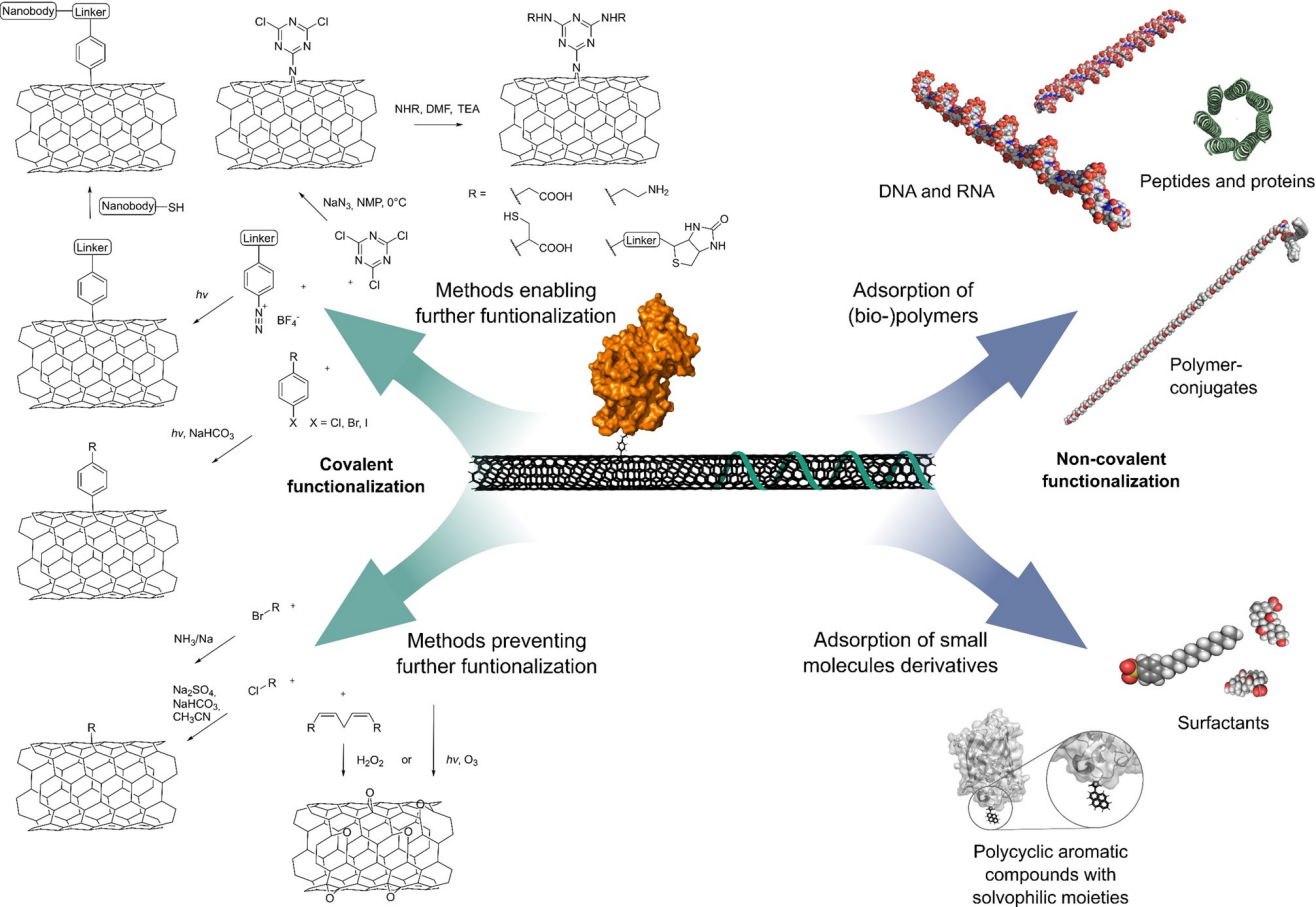
Covalent and noncovalent (bio)functionalization of SWCNTs. Note that only covalent approaches that preserve the NIR fluorescence are included and that double bonds in the SWCNT carbon lattice are not shown for clarity.

In the past years, different covalent and noncovalent modification strategies (Figure 2) have been developed. For a complete overview, we refer the reader to several excellent reviews and discuss only concepts relevant for sensing here.[^26^, ^51^, ^52^, ^53^]

**Figure 3:**
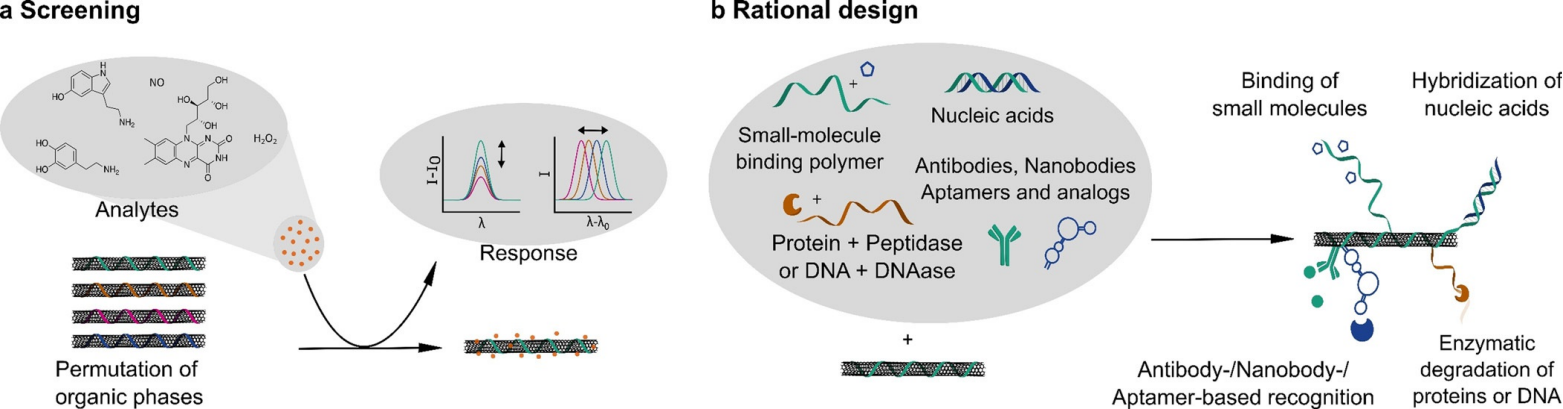
Chemical concepts for the design of SWCNT‐based NIR fluorescent sensors. a) Screening of different organic phases identifies biopolymer/SWCNT conjugates with the desired analyte response. b) Rational concepts use known recognition motifs and assemble them on the SWCNT surface. Note that for both concepts colloidal stability in aqueous solution determines the usable reactions.

On a more abstract level, two strategies to assemble selective SWCNT‐based sensors have been used, namely screening and rational design (Figures 3 and 5). The first one relies on permutations of the organic corona around the SWCNT (e.g. deoxyribonucleic acid (DNA) sequence) whereas the second one uses known recognition motifs (e.g. antibodies).

#### 2.2.1 Noncovalent Functionalization

Noncovalent functionalization in aqueous solution is achieved by sonication with surfactants that form micellar structures around the SWCNT or through strong π‐π interactions with the SWCNT surface (Figure 2). Prominent examples of surfactants are sodium dodecylsulfonate (SDS), sodium dodecylbenzenesulfonate (SDBS), sodium cholate (SC), sodium deoxycholate (DOC), lithium dodecyl sulfate, Triton X‐100, and pluronic F127.[^17^, ^26^] Additionally, functional surfactants—for example, with a perylene core together with a hydrophilic dendron—adsorb through π‐π stacking and enable energy transfer.^[54]^ In general, a surfactant concentration above the critical micelle concentration is required to stabilize dispersed SWCNTs in solution.^[17]^ Thus, these approaches are limited with regards to experiments in complex (biological) systems.

In contrast, functionalization with larger biopolymers enables the formation of stable conjugates. Here, DNA and ribonucleic acid (RNA) form strong π‐stacking interactions between the nucleobases and the SWCNT surface, thereby exposing their negatively charged phosphate backbones and solvating the SWCNT–nucleic acid complex (Figure 2).[^22^, ^55^] As the conformation of the SWCNT–nucleic acid complex is affected by changes in the local ion concentration,[^25^, ^56^, ^57^] locked nucleic acids have been used as more rigid synthetic derivatives at higher salt concentrations.^[57]^

**Figure 4:**
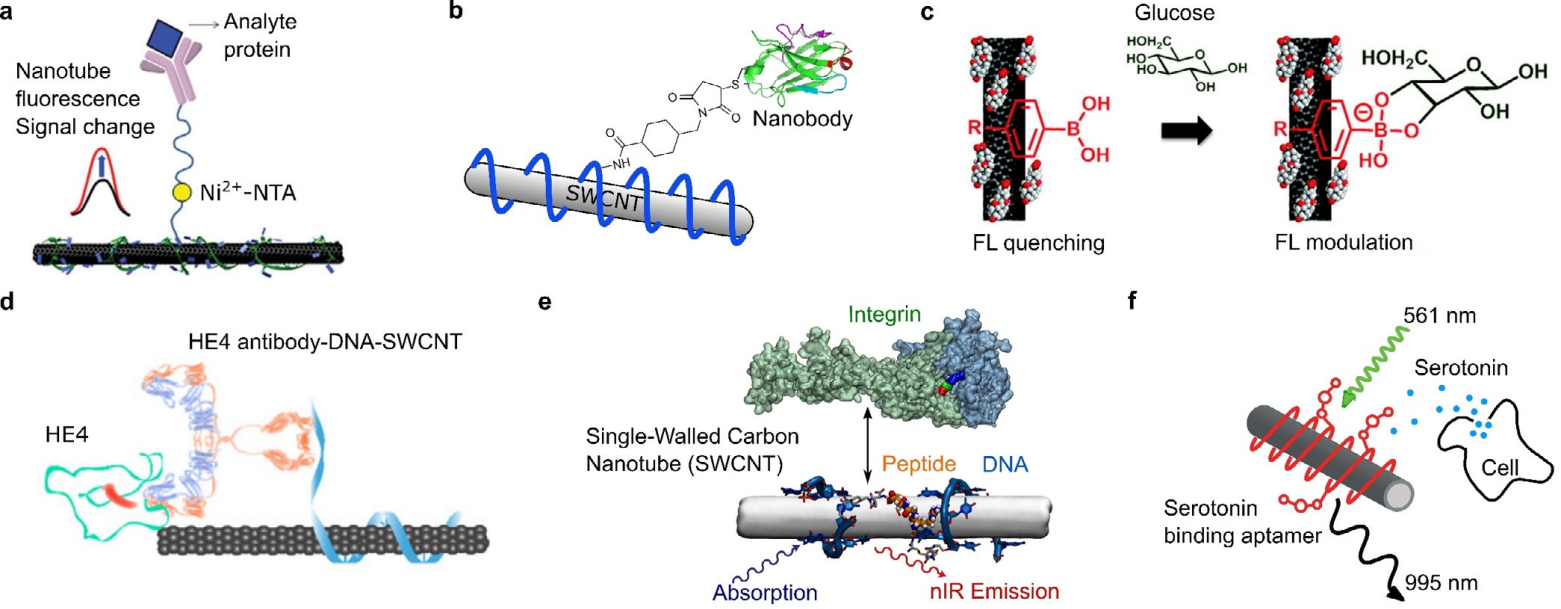
Rational molecular recognition concepts in SWCNT‐based biosensors. a) Conjugation of His‐tagged troponin antibodies to SWCNTs wrapped with Ni^2+^‐chelating chitosan for the detection of the cardiac biomarker troponin. Adapted from Ref. [64] with permission. Copyright 2014 John Wiley and Sons. b) Attachment of a nanobody against the green fluorescent protein (GFP) to DNA‐functionalized SWCNTs to target GFP‐tagged proteins in vivo. Adapted from Ref. [47] with permission. Copyright 2019 John Wiley and Sons. c) Adsorbed aryl boronic acids react with sugars, which modulates the SWCNT fluorescence. Adapted from Ref. [68] with permission. Copyright 2012 American Chemical Society. d) Antibody‐DNA‐SWCNT complex for the detection of the ovarian cancer biomarker human epididymis protein 4 (HE4). Adapted from Ref. [65] with permission. e) Short peptides conjugated to DNA adsorbed on SWCNTs enable the binding of cell adhesion receptors. Adapted from Ref. [66] with permission. Copyright 2018 American Chemical Society. f) Serotonin‐binding aptamers on SWCNTs enable the detection of serotonin release from cells. Adapted from Ref. [27] with permission. Copyright 2019 American Chemical Society.

**Figure 5:**
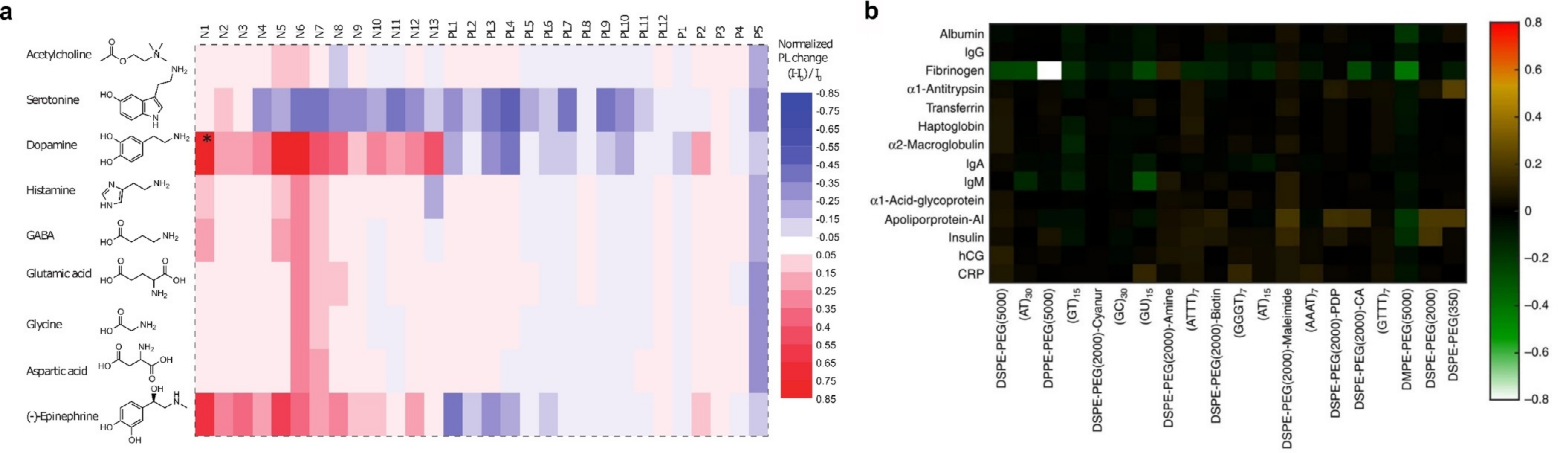
Screening approaches based on corona phase molecular recognition (CoPhMoRe). a) The screening of SWCNT–polymer conjugates (*x* axis, N1–N13: nucleic acids; PL1–PL12: phospholipids; P1–P5: amphiphilic polymers) identifies SWCNT‐based sensor candidates with a strong fluorescence in response to different neurotransmitters (*y* axis). Reprinted from Ref. [15] with permission. Copyright 2014 American Chemical Society. b) CoPhMoRe screening procedure of SWCNT–polymer conjugates (*x* axis) for the detection of proteins (*y* axis). Reprinted from Ref. [71] with permission.

As alternative to nucleic acids,^[25]^ certain polycyclic aromatic compounds carrying hydrophilic moieties have effectively solubilized SWCNTs. Similar to the π‐stacking[^51^, ^58^] of those compounds, the functionalization of SWCNTs with peptides,[^59^, ^60^] proteins,[^60^, ^61^] and other polymers[^62^, ^63^] has been widely demonstrated (Figure 2). SWCNT‐based biosensors have been rationally designed by the attachment of antibodies (or analogues; Figure 4 a,b,d)[^64^, ^65^] and peptides (Figure 4 e)[^66^, ^67^] to polymers or by the adsorption of boronic acids (Figure 4 c)^[68]^ and aptamers (Figure 4 f) on SWCNTs.^[27]^ In cases when sonication would destroy the structural integrity of the (bio‐)polymers, primary suspension of the SWCNTs in a surfactant, followed by subsequent exchange to the polymer by dialysis has been employed.[^24^, ^60^, ^69^] An alternative to this rational design is the screening/search for novel organic phases. This concept was named corona‐phase molecular recognition (CoPhMoRe).^[70]^ Here, a heteropolymer adsorbs onto the carbon nanotube surface and forms a new structure (corona) that serves as a molecular recognition site for interaction with an analyte. The biomolecules used are typically amphiphilic with hydrophobic domains that enable SWCNT adsorption and hydrophilic domains to be responsible for the entropic stabilization of the SWCNT in suspension and formation of a binding site for the analyte.^[70]^ It is important to note that the biomolecules/polymers alone do not necessarily need to interact selectively with the analyte of interest.[^34^, ^70^] As such, the formation of these recognition sites cannot be predicted and are typically found by screening or high‐throughput approaches. Prominent examples of CoPhMoRe screenings are the identification of SWCNT‐based neurotransmitter sensors^[15]^ as well as the adaptation of the CoPhMoRe concept to proteins^[71]^ (Figure 5).

#### 2.2.2 Covalent Functionalization

The covalent functionalization of SWCNTs introduces new σ‐bonds into the sp^2^‐hybridized SWCNT structure. In contrast to noncovalent functionalization methods, the conjugates promise higher stability.^[10]^ However, the uncontrolled introduction of covalent sp^3^ bonds (defects) destroys the electronic and optical properties and diminishes the intrinsic NIR fluorescence.^[10]^ One strategy to overcome this problem preserves the sp^2^‐hybridized structure of SWCNTs during their covalent functionalization.^[72]^ In contrast, a certain number of sp^3^ defects give rise to novel properties, such as red‐shifted emission features that are capable of single photon emission.[^53^, ^73^, ^74^, ^75^] Therefore, these defects are also called quantum defects or quantum color centers.^[53]^

**Figure 6:**
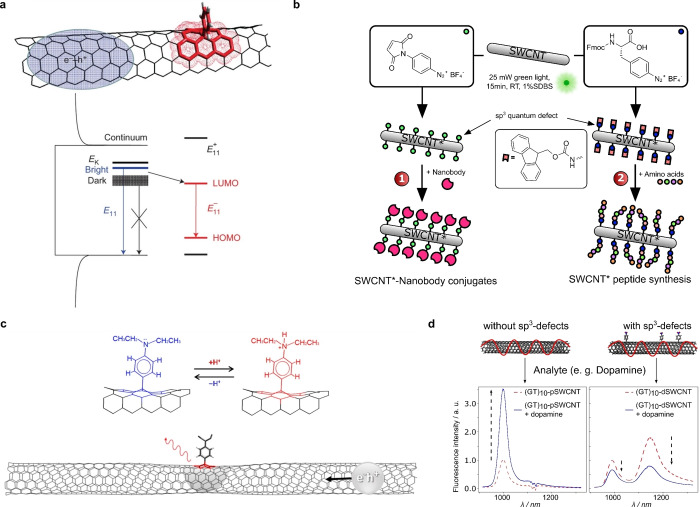
Covalent functionalization of SWCNTs. a) The controlled introduction of sp^3^ defects creates an alternative decay pathway that brightens dark excitons without destroying the normal E_11_ NIR fluorescence. Reprinted from Ref. [74] with permission. Copyright 2013 Nature Publishing Group. b) Introduction of certain aryl defects as generic handles to functionalize SWCNTs with biomolecules. Adapted from Ref. [79] with permission. c) The protonation of covalently attached aminobenzene groups modifies the energy level of the sp^3^ defect state and changes the photoluminescence. Adapted from Ref. [85] with permission. Copyright 2015 American Chemical Society. d) Quantum defects change the fluorescence response of DNA‐functionalized SWCNTs to the important biomolecule and neurotransmitter dopamine. (GT)_10_‐functionalized pristine SWCNTs (pSWCNT) increase their fluorescence in response to dopamine. The same SWCNTs with sp^3^ defects (dSWCNT) decrease their fluorescence, which shows the strong impact of defects on the sensing mechanism. Adapted from Ref. [87] with permission. Copyright 2021 American Chemical Society.

These sp^3^ defects have, at low densities, been shown to increase the fluorescence of SWCNTs.[^53^, ^74^, ^76^] Incorporation of these defects at low concentrations leads to the trapping of excitons and an alternative decay pathway (E_11_^*^) that results in a new red‐shifted fluorescence feature (Figure 6 a).[^74^, ^76^] A wide range of sp^3^ defects has been incorporated into SWCNTs to increase the fluorescent properties by using diazo ether, aryl halide, (bis‐)diazonium, as well as Billup‐Birch and alkyl halide reductions.^[53]^ Additionally, O‐doping approaches using ozone and light,^[75]^ sodium hypochlorite,^[77]^ as well as hydroperoxides of polyunsaturated fatty acids^[78]^ have been reported to increase the red‐shifted emission of SWCNTs at low defect concentrations (Figure 2).

Covalent functionalization approaches also offer opportunities beyond changes in the photophysics. Defects that can be further functionalized enable the conjugation of important biomolecules. Recently, maleimide defects were used to link proteins such as nanobodies and phenylalanine defects to grow peptides directly on the SWCNT surface, similar to a solid‐phase peptide synthesis^[79]^ (Figure 6 b). The covalent conjugation approach with dichlorotriazine allows subsequent nucleophilic aromatic substitution of the chlorides using amine‐containing linkers[^72^, ^80^] (Figure 2). Another example are defects that are already able to interact with other biomolecules, such as phenyl boronic acids that interact with saccharides, and change the E_11_^*^ (S_11_^*^) and E_11_ (S_11_) emissions.^[81]^

It is interesting to note that the resulting bathochromic shifts (E_11_^*^) caused by sp^3^ defects can be tuned using the electronic properties of the incorporated moieties.[^74^, ^76^] That said, defects provide a rich chemical playground and interested readers are referred to several excellent reviews.[^53^, ^82^] With regards to SWCNTs with aryl defects, electron‐withdrawing substituents generally introduce red‐shifts to the E_11_^*^ emission that can be correlated to the Hammet constants (σ) of the substituents.[^74^, ^83^] Furthermore, the E_11_^*^ red‐shift shows a 1/*d* ^2^ dependence on the diameter (*d*) of the SWCNT.^[74]^ The protonation of diethylamino‐substituted aryl defects (σ_HR2N+_=+0.82 vs. σ_R2N_=−0.66)^[84]^ is a good example of the effect caused by the inductive effects of substituents. Furthermore, this type of defects allows a precise sensing of the pH value down to 0.2 units through the changes in the E_11_^*^ emission (Figure 6 c).^[85]^ Apart from the introduction of defects for sensing, the covalent functionalization of SWCNTs can be used for site assembly using different (bio‐)polymers and linkers.^[86]^

Defects also change the exciton decay pathways and, therefore, affect the photophysics and sensing mechanism of SWCNT‐based sensors. This approach was recently used to perturb the sensing and elucidate the rate constants that are involved^[87]^ (Figure 6 d).

In this study it was also found that a small number of defects can inverse the sensing response from a strong increase to a strong decrease in fluorescence.

### 2.3 SWCNT Biocompatibility

Biocompatibility is highly important for materials in direct contact to biological matter. Even though many biocompatibility studies exist, the conclusions are difficult to compare.^[88]^ The main reason is that different materials, surface reactions, and biological systems are compared, which leads to a noncoherent view. Moreover, the SWCNT field has evolved dramatically over the years and well‐defined chirality pure SWCNTs‐based sensors with ultrahigh purity are available today, whereas older studies used less well‐defined materials.^[49]^ In addition, the application itself determines the perspective.^[89]^ As a research tool, SWCNTs should not affect the biological system in such a way that the results are biased. For long‐term in vivo studies and applications in humans, the fate of SWCNTs in biological systems is highly relevant for their biocompatibility. SWCNTs have been shown to be susceptible to degradation by oxidative processes introduced by neutrophils^[90]^ and macrophages.^[91]^ Furthermore, the functionalization changes the surface properties of the nanotubes and, thus, ultimately the way SWCNTs interact with the molecules in a (biological) system.^[92]^

For example, endocytosis experiments have shown that the DNA sequence length plays an important role in endocytosis and retention time scales of DNA‐functionalized SWCNTs within mammalian cells.^[93]^ Additionally, experiments using a combination of NIR fluorescence spectroscopy and resonance Raman scattering have been used to analyze the fate of DNA‐functionalized SWCNTs through the endosomal process.^[94]^ Based on the experimental findings, the authors propose that DNA‐SWCNTs enter the cell, where they are transported into early endosomes. Maturation of the endosome begins with a decrease in the luminal pH value, which is followed by a series of physicochemical processes that transform the endosome into a lysosome, where the SWCNTs finally aggregate.^[94]^

As correct functionalization has been shown to alleviate the pathogenicity of SWCNTs,^[95]^ stable functionalization is one possible way to safeguard the future design of SWCNT‐based sensors in environments where long‐term stability is of the highest importance. Adequately functionalized SWCNTs have been shown to possess excellent biocompatible properties. A good example is the recently published long‐term biodistribution and compatibility assessment of DNA‐encapsulated SWCNTs after intravenous administration in mice.^[96]^ After an initial increase in the SWCNT fluorescence in the liver, the SWCNT fluorescence decreased rapidly over the course of 14 days.^[96]^ The same trend is also seen in the long‐term SWCNT biodistribution in different organs. By using hyperspectral microscopy, low levels of SWCNTs were detected in murine hearts, lungs, livers, kidney, and spleen tissues one month after injection. Assessment of these tissues after three and five months showed no SWCNT fluorescence in lung tissue, or in heart and lung tissues.^[96]^ Moreover, no abnormalities were found in chronically exposed tissues after hematoxylin** **and eosin (H&E) staining at all observed time points and the assessed biomarkers showed negligible changes up to four months, and minor changes after five months.^[96]^

The aforementioned studies suggest remarkable opportunities for SWCNTs in biomedical applications. As a consequence of the interplay between different materials, surface reactions, and biological systems it becomes evident that biofunctionalized SWCNTs represent a class of different materials. As for all new materials, the biocompatibility should be evaluated for every type of chirality, purity, functionalization, and route of administration.^[96]^ The scientific community is well‐aware of this problem and it has been pointed out that an assessment of these parameters in the context of biocompatibility depends on the context of the experiment, timescale, and the application of the nanomaterial.[^88^, ^97^] As a consequence, it is fundamentally important to place experimental data in the right context.^[88]^

An important requirement for a biocompatible design of SWCNT‐based sensors is an in‐depth understanding of the composition of the protein corona in biological media. In this regard, a recent study characterized the enrichment of certain proteins in the SWCNT corona.^[98]^

In the future, long‐term studies comparing the biocompatibility of different SWCNT subclasses (purity, chirality, surface chemistry) would be desirable to safeguard the development of biocompatible sensors. A foundation for the standardization of protocols could be the MIRIBEL (Minimum Information Reporting in Bio‐Nano Experimental Literature) reporting standard.^[99]^ As the functionalization of the SWCNT plays a critical role in the biocompatibility of SWCNT‐based sensors, the design of stable SWCNT functionalizations needs to be carefully ensured for long‐term applications. In particular, the recent advances in covalent functionalization strategies might, therefore, offer interesting starting points for future development.^[79]^

## 3 SWCNT‐Based Sensors

### 3.1 Development of Chemical Design Strategies

The discovery of band gap fluorescence from SWCNTs and their structure‐dependent NIR emission wavelength marks the starting point for SWCNT‐based sensors.[^12^, ^18^] Given the high surface to volume ratio of SWCNTs, it was quickly anticipated that SWCNT fluorescence would be sensitive to the chemical environment.^[24]^ The first generation of sensors targeted mainly smaller molecules including protons and reactive oxygen/nitrogen species (ROS/RNS). In these cases, the fluorescence changes were most likely caused by direct quenching. At the same time, it was discovered that different surface reactions with biopolymers lead to molecular interactions that are surprisingly specific even without using a standard approach with antibodies. This idea was conceptualized as corona‐phase molecular recognition (CoPhMoRe).^[70]^ During the last few years, great progress has been made in the chemical design of sensors for both biomedical and environmental applications. In the next sections we will give an overview on different sensing strategies, organized according to the molecular target. Here, sensing of the target molecule has partly environmental as well as biomedical applications. We focus on the advances in the last few years but also report previous studies (see Tables 1–, 9 in the Appendix).

### 3.2 Biosensing of Target Analytes

The ongoing advances in the development of recognition strategies have led to powerful biosensors based on SWCNTs. Various targets can be detected with high selectivity and sensitivity by combining a recognition unit with the SWCNTs. Recognition strategies (Figure 3) can mainly be categorized into a screening (Figure 5) and a rational approach (Figure 4). The first approach is in principle achieved with a library of synthetic organic phases (coronas) consisting of different amphiphilic polymers wrapped around SWCNTs and screened against a panel of various analytes to find a selective interaction.[^15^, ^70^, ^71^, ^100^, ^101^] The latter approach is mostly applied for the detection of larger molecules such as proteins[^65^, ^102^, ^103^, ^104^, ^105^, ^106^] or sugars[^11^, ^107^, ^108^] by conjugating a known binding partner of the target analyte to the SWCNT surface. Several approaches are based on the use of SWCNTs wrapped by single‐stranded DNA (ssDNA), whereby different lengths of the (GT)_*x*_ sequence is probably the most used sequence up to now.

It has shown its versatility in the detection of divalent ions,[^25^, ^109^] genotoxins,^[110]^ NO,^[20]^ H_2_O_2_,[^20^, ^32^, ^110^, ^111^, ^112^] riboflavin,[^49^, ^113^, ^114^] doxorubicin,^[115]^ β‐carotene,^[116]^ endolysosomal lipids,^[117]^ arsenite,^[33]^ and neurotransmitters, especially dopamine.[^15^, ^49^, ^63^, ^80^, ^87^, ^118^, ^119^, ^120^, ^121^] This section highlights the major advances from the last few years for different categories of biomolecules. A detailed overview of most of the known fluorescent SWCNT‐based sensors, subdivided into the target categories, can be found in Tables 1–9 in the Appendix.

#### 3.2.1 ROS/RNS

ROS/RNS are important signaling molecules in many organisms,^[122]^ but their detection is challenging because they diffuse fast and have short lifetimes due to their high reactivity with O_2_ and other molecules.[^10^, ^123^] Since the finding of the first NO sensor based on SWCNTs^[124]^ the performance of SWCNT‐based ROS sensors has grown from the first selective detection of NO and H_2_O_2_ at the single molecule level[^13^, ^125^, ^126^] and first in vivo applications^[127]^ to a new approach to study NO generation and spatiotemporal imaging of intracellular NO signaling.^[128]^ Recently, a mathematical model that calculated the NO concentration based on the change in the SWCNT fluorescence was derived.^[129]^ This was previously not possible due to a nonlinear fluorescence quenching rate in response to NO.^[13]^

**Figure 7:**
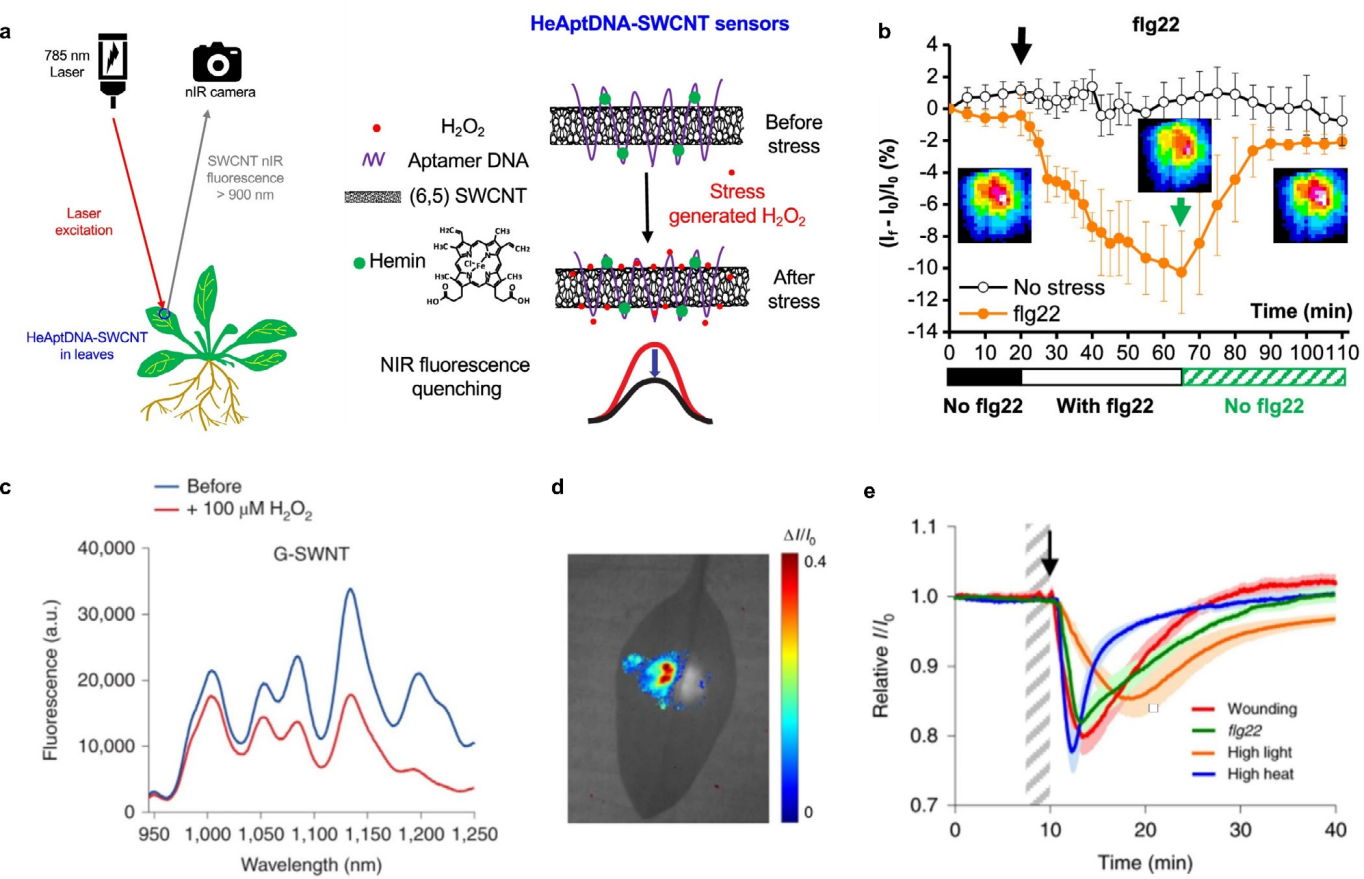
In vivo monitoring of plant health. a) SWCNTs functionalized with a DNA aptamer for hemin (HeAptDNA‐SWCNT) serve as a sensor for H_2_O_2_, which is an important signaling molecule for plant stress. Hemin catalyzes the reaction of H_2_O_2_ to hydroxyl radicals, which quench the fluorescence of the SWCNT. Spatial and temporal changes in the NIR fluorescence intensity in leaves embedded with HeAptDNA‐SWCNT sensors are remotely recorded by a NIR camera to assess plant health. b) The sensor's NIR fluorescence decreases reversibly in the presence of the peptide flg22, which mimics a pathogen attack. Reprinted from Ref. [31]. Copyright 2020 American Chemical Society. c) SWCNTs functionalized with (GT)_15_‐ssDNA (G‐SWNT) respond to H_2_O_2_. d) Bright‐field image of a spinach leaf infiltrated with G‐SWNT (left) and nonresponsive A‐SWNT (right) in combination with a false color plot after wounding shows that only the G‐SWNT spot decreases in intensity. e) Ratiometric sensor response after application of different types of stress. Reprinted from Ref. [32] with permission. Copyright 2020 Springer Nature.

ROS play a mediating role in the cell‐to‐cell communication of plants to activate defence mechanisms,^[122]^ whereby it has become clear that H_2_O_2_ is the primary mediator that responds to different stresses in plants.^[130]^ This has led to novel SWCNT sensor approaches to study ROS within plants.[^19^, ^20^, ^31^, ^32^] Wu et al. demonstrated remote H_2_O_2_ monitoring of plant health with sensitivity in the plant physiological range by using fluorescent SWCNT‐based sensors.^[31]^ Their rational approach was based on a DNA aptamer that specifically binds to the porphyrin hemin (HeAptDNA‐SWCNT). Hemin binds ferric ions, which undergo a Fenton‐like reaction with H_2_O_2_ to produce hydroxyl radicals (Figure 7 a) that directly quench the SWCNT fluorescence. For spatiotemporal in vivo monitoring, SWCNTs were embedded in leaves of plants and the plants exposed to different stresses such as UV‐B, high light intensities, and a pathogen‐associated peptide (flg22; Figure 7 b). The decrease in fluorescence reported remotely different aspects of the stress. These differences in fluorescence intensity quenching offer the possibility to interpret stress patterns in plants.

Similar to this approach, Lew et al. developed a platform for H_2_O_2_ detection in leaves of different plant species.^[32]^ This sensor platform used a ratiometric approach, with (GT)_15_‐SWCNTs (G‐SWNT) that respond to H_2_O_2_ by quenching, possibly as a result of a charge‐transfer phenomenon, and (AT)_15_‐(6,5)‐SWCNTs (A‐SWNT) as an invariant reference (Figure 7 c). They infiltrated both G‐SWNT and A‐SWNT into spinach leaves and monitored the H_2_O_2_ signal with a standoff detection platform in real time (Figure 7 d). In the presence of different stresses, for example, tissue wounding, distinct waveform characteristics were observed (Figure 7 e), whereby the wave speeds in different plant species post‐wounding differed in the range of 0.44 to 3.10 cm min^−1^.

**Figure 8:**
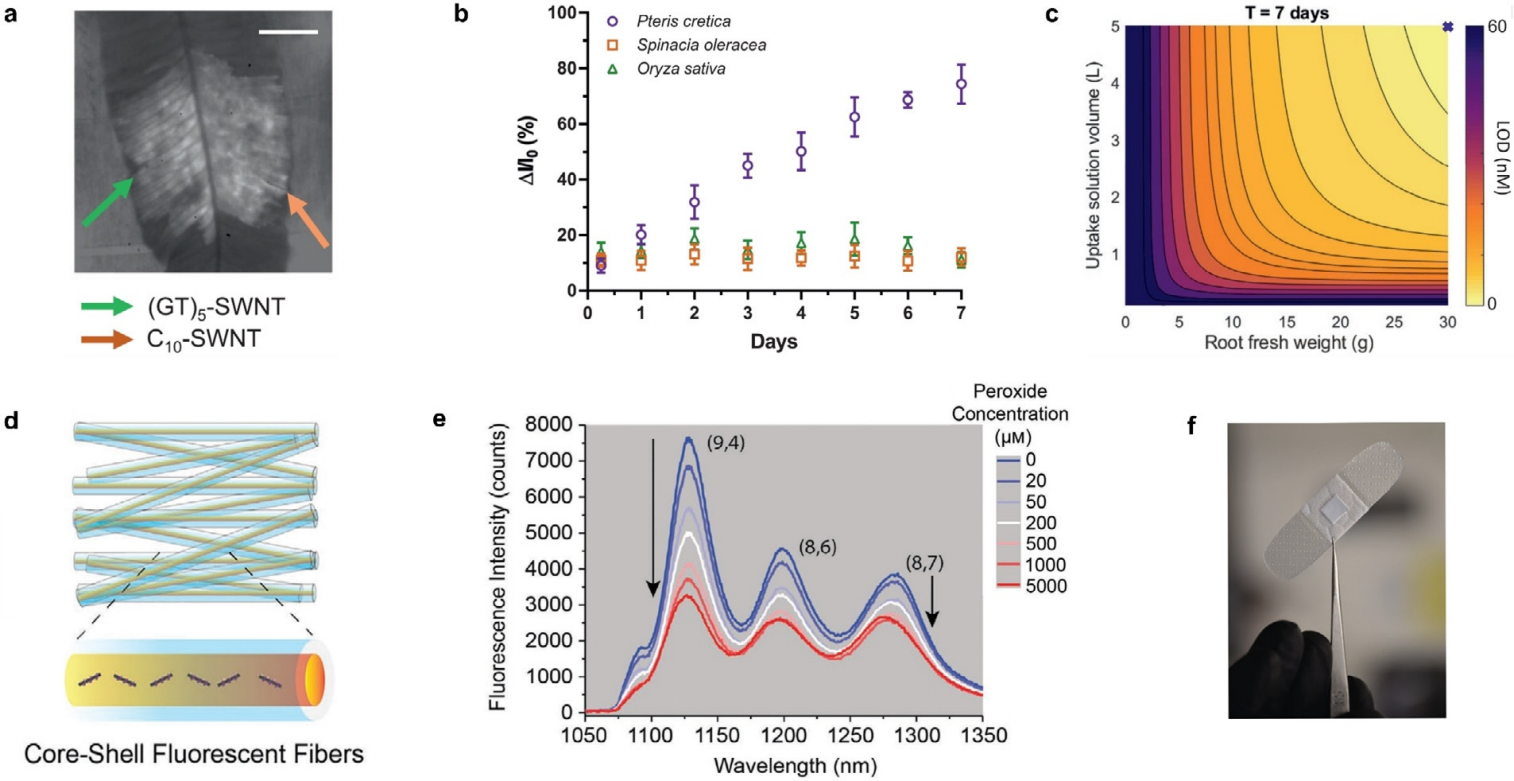
Macroscopic detection of small molecules. a) Bright‐field image of leaf (arsenic hyperaccumulator plant Pteris cretica) infiltrated with (GT)_5_‐SWCNT sensors and a C_10_‐SWCNT reference. b) Fluorescence intensity change in response to 10 μm arsenite uptake through the roots of a hyperaccumulator, spinach, and rice plants. c) Theoretical LOD as a function of root fresh weight and uptake solution volume after 7 days. Reprinted from Ref. [33] with permission. Copyright 2020 John Wiley and Sons. d) Wireless monitoring of oxidative stress. Schematic representation of fabricated core–shell NIR fluorescent microfibers. e) Fluorescence spectra of the microfibrous material in response to different peroxide concentrations. f) Optical fibrous sample integrated into a commercial wound bandage for real‐time wireless sensing. Adapted from Ref. [112] with permission. Copyright 2021 John Wiley and Sons.

In the same manner, Lew et al. developed a SWCNT‐based sensor system for the specific detection of arsenite in plants to monitor the uptake of the toxic heavy‐metal pollutant arsenic by using a self‐powered microfluidic system in real time.^[33]^ For this purpose, they infiltrated the sensors and the invariant reference into leaves of spinach, rice plants, and hyperaccumulating fern, which is able to preconcentrate and extract arsenic from soil (Figure 8 a). The intensity of the sensors increased steadily over several days, with the sensor response of the hyperaccumulating plant being significantly higher than those of the rice and spinach plants (Figure 8 b). Based on a kinetic model, the arsenite concentration in the leaf and the limit of detection (LOD) were calculated to be 4.7 nm and 1.6 nm as a function of the root fresh weight and uptake solution volume after 7 and 14 days (Figure 8 c). These examples show that SWCNT‐based H_2_O_2_ sensors are able to report plant stress on a microscopic and macroscopic level with potential applications in smart agriculture.

Another macroscopic situation in which H_2_O_2_ plays an important role is wound healing. Safaee et al. developed a wearable optical microfibrous material with encapsulated SWCNT‐based sensors (Figure 8 d) to monitor the H_2_O_2_ concentration in wounds.^[112]^ Their approach was based on the ratiometric signal of (8,7)/(9,4)‐SWCNT chiralities, which differed in their response to H_2_O_2_ (Figure 8 e). The fluorescence signal was invariant to the excitation source distance, and exposure time, which enabled detection within commercial wound bandages with a wireless readout (Figure 8 f). These microfibers encapsulated the SWCNTs for at least 21 days without structural changes.

Furthermore, Zheng et al. reported the selective interactions of SWCNTs coated with ten different ssDNA sequences in response to dissolved oxygen.^[131]^ The SWCNT emission intensity was quenched between 9 to 40 % depending on both the ssDNA sequence and SWCNT chirality in response to 1 atm O_2_ compared to samples purged with 1 atm argon, thus indicating that stronger coating interactions lead to reduced O_2_ access to the SWCNT surface. Since the quenching reversed completely after the removal of dissolved oxygen, it is probably based on physisorption on the SWCNT. Thus, the screening for fluorescence quenching by dissolved oxygen provides a simple approach to explore the structure‐selective interactions of ssDNA with SWCNTs.

ROS can also be generated by enzymes and SWCNTs. Yaari et al. demonstrated the first SWCNT‐based sensor that reports the degree of enzymatic suicide inactivation.^[201]^ The approach was based on enzyme‐bound SWCNTs, which report fluorescence modulations by quenching and red‐shifting selectively in response to substrate‐mediated suicide inactivation of tyrosinase. Mechanistic insights revealed that the red‐shifted response is most likely a result of the generation of singlet oxygen during the enzymatic reaction, which leads to the binding of ssDNA on the SWCNT surface.^[132]^

#### 3.2.2 Neurotransmitters

Neurotransmitters are an important class of signaling molecules. To understand neuronal networks and linked neurological diseases, imaging with high selectivity and spatiotemporal resolution is necessary, which existing methods are currently not able to provide.^[133]^ In the last few years several SWCNT‐based sensors based on functionalization with DNA have been explored and it has been shown that sensitivity and selectivity depend on the exact DNA sequence.[^100^, ^118^, ^119^]

**Figure 9:**
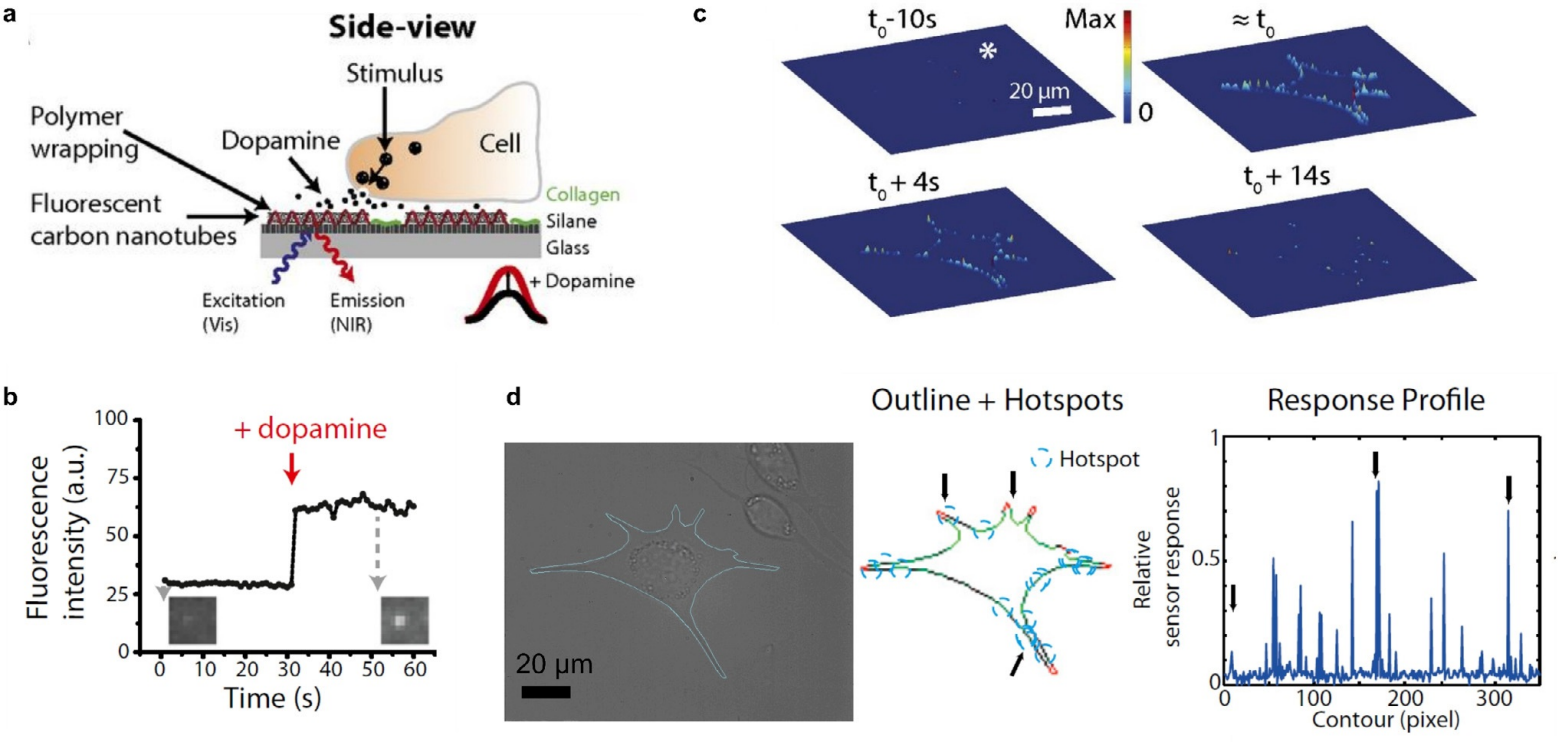
High‐resolution imaging of dopamine using SWCNT‐based sensor arrays. a) Specific ssDNA‐functionalized fluorescent SWCNTs respond selectively to dopamine. Sensors are immobilized on a glass substrate and dopamine‐releasing neuroprogenitor cells are cultivated on top. The SWCNT fluorescence changes in response to stimulation of the cells. b) Fluorescence intensity change of a single sensor in response to dopamine. c) Three‐dimensional release profiles for dopamine along the border of neuroprogenitor (PC12) cells at different time points relative to the stimulation at *t*_0_. The height and color indicate the relative fluorescence change normalized to the maximum fluorescence change. d) Bright‐field image of the cell stimulated on top of the nanosensor array and corresponding hotspots (blue circles) along the cell border. Arrows indicate positions belonging to the hotspots in the response profile. Reprinted from Ref. [100] with permission.

The first SWCNT‐based sensors for the detection of neurotransmitters were reported by Kruss et al.^[15]^ By using a screening approach, it was found that certain ssDNA‐SWCNTs change their fluorescence in the presence of catecholamine neurotransmitters such as dopamine. These sensors were reversible and showed sensitivities in the nanomolar range. Similar sensors were used for the high‐resolution imaging of cellular dopamine efflux from stimulated neuroprogenitor cells.^[100]^ Here, the sensors were immobilized on a collagen‐coated glass substrate to increase cell adhesion, and dopamine‐releasing neuroprogenitor (PC12) cells were cultured on top. In response to a stimulation event, the fluorescence intensity of the sensor layer image consisting of up to 20 000 pixels increased (Figure 9 a,b). This allowed both the spatial and temporal dynamics of dopamine release events to be studied with extraordinary high resolution and to identify hotspots (Figure 9 c,d).

**Figure 10:**
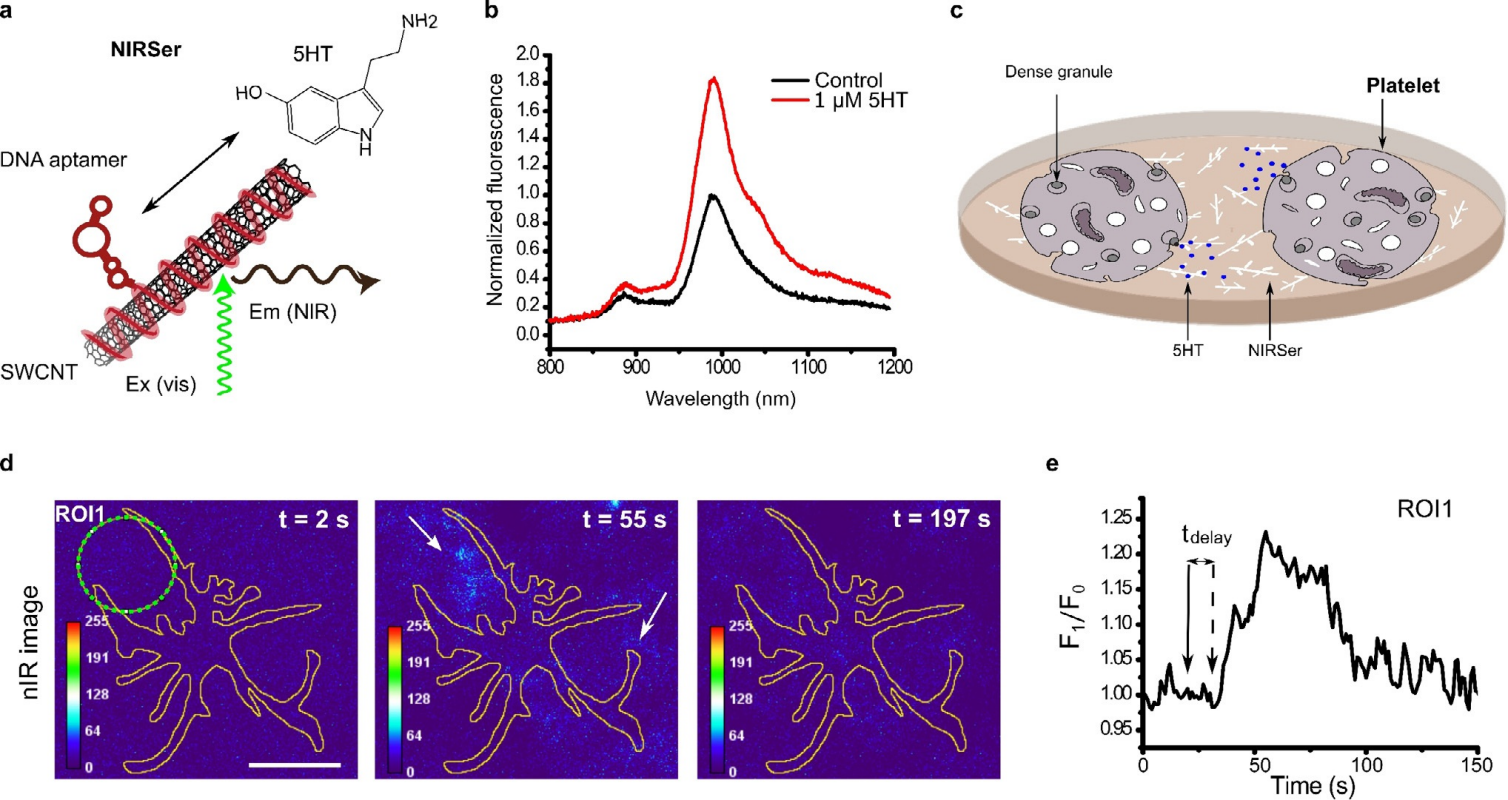
High‐resolution imaging of serotonin (5HT) release from cells. a) NIRSer sensor: SWCNTs functionalized with a serotonin aptamer respond selectively to serotonin. b) NIRSer increases its fluorescence in response to serotonin. c) Sensors are immobilized on a surface and serotonin‐releasing cells are cultured on top. Here, blood platelets are used, which contain most of the body's serotonin. d) Color‐coded image of serotonin release from a single platelet at three time points (before, during, and after serotonin release). e) Fluorescence response from a region of interest (ROI, green circle in (d)). The activation and delay time of the onset of serotonin release are marked with arrows. Reprinted from Ref. [27] with permission. Copyright 2020 American Chemical Society.

This approach of imaging many nanosensors under cells is also applicable to other neurotransmitters. Dinarvand et al. imaged the release of serotonin from human blood platelets in real time,^[27]^ as most of the serotonin is stored in blood platelets and not in the brain of humans. This serotonin sensor (NIRSer) consisted of a serotonin‐binding aptamer on a SWCNT, which exhibited an increased fluorescence emission of up to 80 % in response to serotonin (Figure 10 a). High‐resolution images of serotonin release patterns from single cells were obtained by placing the sensors below and around serotonin‐releasing cells (Figure 10 b,c). This approach allows serotonin release to be studied with unprecedented resolution and the time delay between stimulation and release to be resolved.

In a similar fashion, artificially added serotonin was detected in acute brain slices by Jeong et al.^[134]^ In this case, a DNA sequence was found by an expanded screening approach from a library of around 6.9×10^10^ different ssDNA‐SWCNTs. These ssDNA‐SWCNTs exhibited a selective response to serotonin over serotonin analogues, metabolites, and receptor‐targeting drugs.

The high spatiotemporal resolution of SWCNT‐based neurotransmitter sensors is especially useful when it comes to resolving parallel processes on the subcellular to cell‐network length scale. However, placing the sensors below cells can be a drawback, especially with cells that need to differentiate on this layer for several weeks. Elizarova et al. developed a new sensor paint approach (AndromeDA) to use sensors and study the dopaminergic signaling in primary neurons.^[135]^ In this case, the sensors were adsorbed (′painted′) onto the complex cell networks, which included different cell types. This approach allowed the heterogeneity of dopamine release events to be quantified from up to 100 release sites (varicosities), which is highly important to understand the information processing and plasticity of neurons.

An effect that has to be accounted for in these studies is that SWCNT fluorescence is affected by changes in the local cation concentration, which is also a hallmark of neuronal activity.[^56^, ^57^] To circumvent this problem, Gillen et al. used locked nucleic acids to develop sensors with improved stability to cation‐induced fluctuations of the fluorescence intensity.^[136]^ By systematically introducing locked bases along the (GT)_15_‐DNA sequence they found that the fluorescence stability in the presence of Ca^2+^ ions depends on the type of the locked bases. Certain SWCNT chiralities exhibited improved stability against Ca^2+^ ions and retained their ability to detect dopamine in the presence of Ca^2+^ ions, thus highlighting the importance of the exact conformation of the nucleic acid sequence. Moreover, the detection of both Ca^2+^ and dopamine was possible by monitoring multiple chiralities simultaneous.

Interestingly, the fluorescence responses of SWCNTs suspended in sodium cholate to dopamine and serotonin can be altered by modulating the exposed area by the surfactant concentration. However, such surfactants would not be compatible with cellular systems.^[137]^

A central challenge in biomedicine is the controlled delivery (uptake, transport, and release) of (nano)materials such as sensors and pharmaceuticals. Even whole cells can serve as vehicles to take up such materials. Certain immune cells (neutrophils) have been shown to be suitable for cargo delivery by hijacking a process known as neutrophil extracellular trap formation (NETosis). During NETosis, cells lyse after rupture of the cellular membrane by chromatin swelling.^[138]^ Meyer et al. showed that human immune cells take up ssDNA‐SWCNT‐based sensors and can be triggered to release the cargo after a certain time by using NETosis.^[139]^ Moreover, the sensors maintained their functionality to detect dopamine and H_2_O_2_, which offers opportunities for in vivo delivery.

All these discussed neurotransmitter sensors responded by a fluorescence increase. Interestingly, the introduction of a small number of aryl defects into ssDNA‐functionalized SWCNTs completely reversed the sensing response (Figure 6 d).^[87]^ The E_11_ emission slightly decreased and strongly decreased the red‐shifted E_11_^*^ emission. Apart from new insights into the sensing, this approach enables ratiometric detection schemes. For a more detailed overview on the biological relevance of catecholamine neurotransmitters and alternative detection methods (e.g. electrochemical) readers are referred to the literature.[^28^, ^114^]

#### 3.2.3 Other Small Molecules

Beyond neurotransmitters, recognition strategies for other small molecules have been developed, for example, adenosine 5′‐triphosphate,^[140]^ nitroaromatics,[^30^, ^141^] riboflavin,[^49^, ^70^, ^142^] l‐thyroxine,^[70]^ oestradiol,^[70]^ doxorubicin,[^115^, ^143^] and steroids.^[144]^

Finding a specific molecular recognition element is difficult for many of these biomolecules, such as hormones, due to their chemical similarity. Therefore, Lee et al. used a polymer self‐templating synthetic approach, which is based on the attachment of a chemical appendage similar in molecular weight and structure to the target analyte to create a binding pocket within the corona. This approach reduced the library size for screening and led to implantable SWCNT‐based sensors for the selective detection of the human steroid hormones cortisol and progesterone.^[144]^

**Figure 11:**
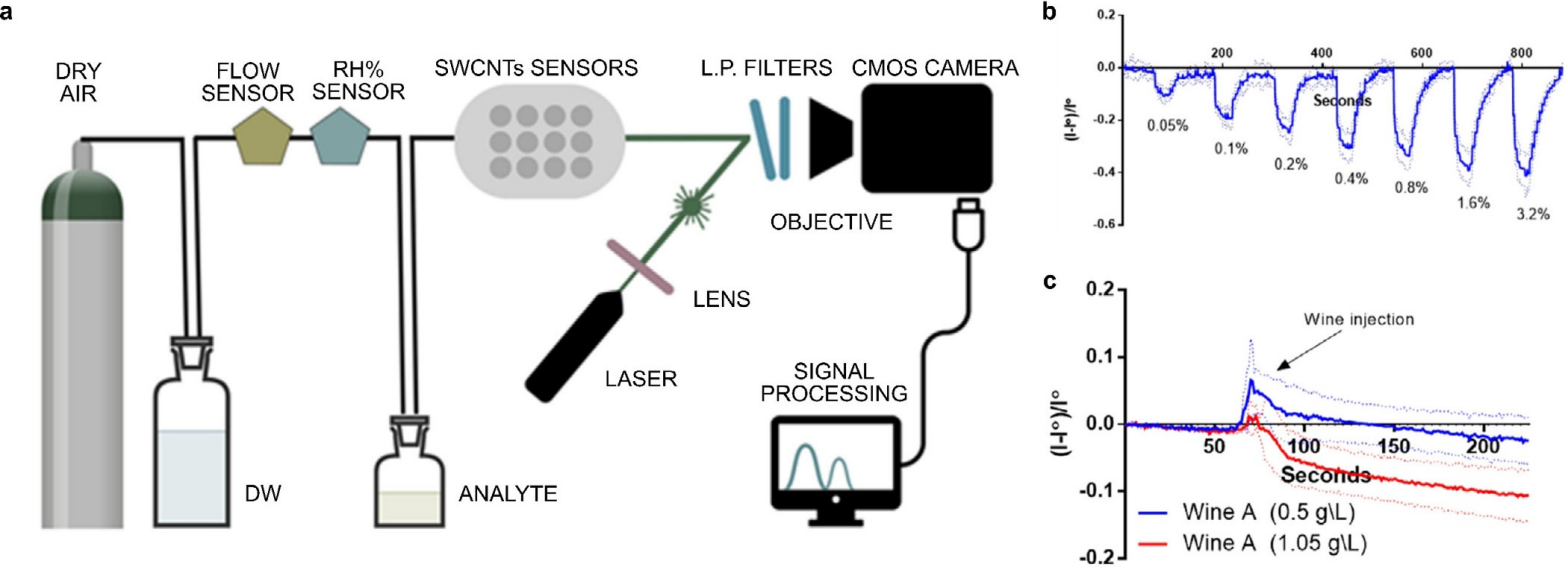
Detection of volatile compounds in the gas phase. a) Air with analyte (acetic acid) flows to the SWCNT‐based sensors, which produces an optical readout. b) Dynamic response of the sensor to rising acetic acid concentrations. c) Dynamic response of the sensor to wine spiked with acetic acid. Reprinted from Ref. [145] with permission. Copyright 2021, Elsevier.

Recently, the first reversible fluorescent SWCNT‐based sensor for volatile organic compounds was also reported, which has potential for the detection of wine spoilage.^[145]^ For this, Shumeiko et al. used peptide‐encapsulated SWCNTs, which were adsorbed onto a polystyrene cuvette to detect low concentrations of acetic acid (down to 0.05 % (v/v)) in air. Using (6,5)‐SWCNTs, which fluoresce below 1000 nm they demonstrated the detection with a low‐cost Si‐based camera (Figure 11 a). The sensor was exposed to different concentrations of acetic acid, which quenched the fluorescence but was reversible when switching to clean air (Figure 11 b). The ability to identify wine spoilage was investigated by using two wine types with and without the addition of acetic acid to simulate an undesirably high acetic acid concentration (Figure 11 c).

In an extension of this study, this system was expanded to an array of five different peptide‐encapsulated SWCNTs on a nitrocellulose paper. The optical patterns enabled the distinction of volatile molecules such as ethanol, methanol, and 2‐propanol as well as the aromas of red wine, beer, and vodka by linear discriminant analysis and machine learning.^[146]^

One way to detect or study small molecules is to mimic parts of larger biomolecules, such as enzymes. Dong et al. screened a library of 24 amphiphilic polymers to find a corona phase which demonstrates a binding specificity very similar to the enzyme phosphodiesterase type 5 (PDE5), which catalyzes the hydrolysis of secondary messengers.^[147]^ The SWCNT‐based sensor consisted of a poly(methacrylic acid‐*co*‐styrene) motif. This synthetic corona mimics the H loop of the native enzyme and is, thus, able to bind to Vardenafil, a PDE5 inhibitor, and its molecular variant as a result of the unique corona phase configuration. It is selective over other off‐target inhibitors, but not completely over the chemically similar inhibitor Sildenafil.

One of the challenges in SWCNT‐based sensing is the heterogeneous material that is used for most sensors. Even though purification has made tremendous progress, getting access to chirality‐pure SWCNTs with a tailored surface chemistry has been a challenge. Nißler et al. showed sensing of small molecules such as riboflavin and ascorbic acid as well as pH value with chirality‐pure SWCNTs by using aqueous two‐phase extraction and a subsequent surface functionalization exchange process.^[49]^ The chirality‐pure sensors were up to ten‐times brighter than mixtures of SWCNT chiralities, and enabled insights into the impact of chirality and handedness of SWNCTs and the sensing mechanism. Additionally, long‐time stability over 14 days was demonstrated as well as ratiometric and multiplexed sensing based on the non‐overlapping fluorescence spectra (Figure 22 b,c).

**Figure 12:**
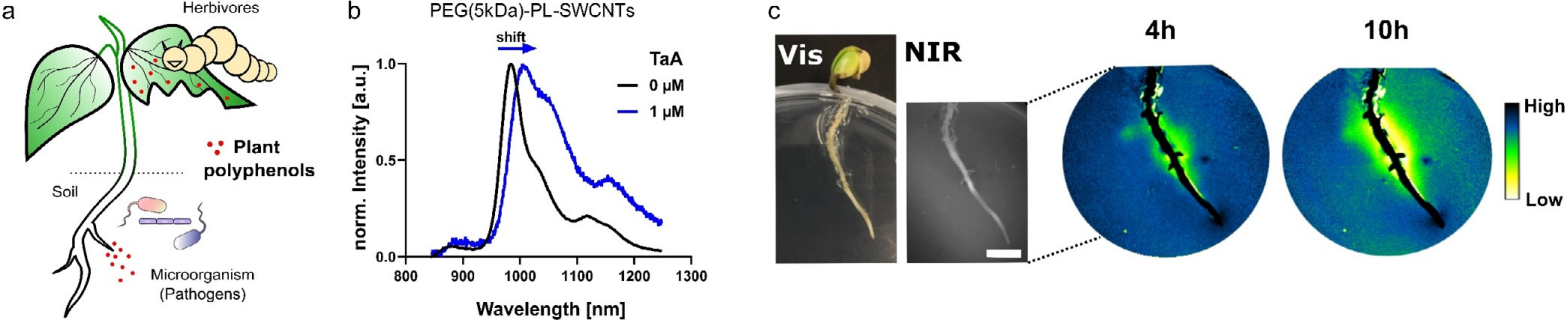
Detection and imaging of plant polyphenols. a) Polyphenols from leaves and roots are released in the immediate vicinity in response to pathogens or herbivores as a defence mechanism. b) SWCNTs functionalized with polyethylene glycol phospholipids (PEG‐PL) respond selectively to polyphenols such as tannic acid (TaA) through a red‐shift and decrease in the fluorescence emission. c) Visible and NIR images of a soybean root plated on top of the sensors (embedded in agar). The plant is challenged with a pathogen elicitor and the NIR fluorescence intensity decreases over time in response to the release of polyphenols in the immediate vicinity of the root. Reprinted from Ref. [148] with permission.

On a macroscopic level, monochiral SWCNTs were used by Nißler et al. to detect polyphenols in and around plants.^[148]^ Polyphenols are secondary metabolites and messenger molecules that are released from leaves and roots as a chemical defence against pathogens and herbivores (Figure 12 a). Certain polyethylene glycol phospholipids (PEG‐PL) were identified for the selective detection of different polyphenols over interfering molecules such as sugars or H_2_O_2_. The SWCNT‐based sensors responded through quenching and red‐shifting of up to 20 nm, for example, to tannic acid (TaA, Figure 12 b). To image the plant polyphenol release over time, the sensors were embedded in agar, soybean seedlings were plated on top, and polyphenol secretion was triggered with a pathogen‐derived elicitor, which resulted in a decrease in the NIR fluorescence over time (Figure 12 c). These sensors help to understand this plant defence mechanism and could improve the breeding of stress‐resistant plants for precision agriculture.

#### 3.2.4 Lipids

The investigation of lipid‐linked diseases is challenging because methods for accurate in vivo monitoring of lipid accumulation have been missing. The Heller group was the first to address this issue by developing a SWCNT‐based optical sensor, which non‐invasively detects the lipid flux within the lumen of endolysosomal vesicles in vitro and in vivo.[^117^, ^149^] In a first approach, they used (GT)_6_‐functionalized (8,6)‐SWCNTs, which fluoresce at 1200 nm and responded through a wavelength shift to biological lipids and water‐soluble lipid analogues.^[117]^

By incubating the sensors with fibroblasts from a lysosomal storage disorder Niemann‐Pick‐type C patient in vitro, the sensors localized in the lumen of endolysosomal organelles without affecting their properties and resolved the lipid accumulation down to the subcellular level in real time. The authors proved the reversibility of this sensor by administering a drug which reverses the disease phenotype.

**Figure 13:**
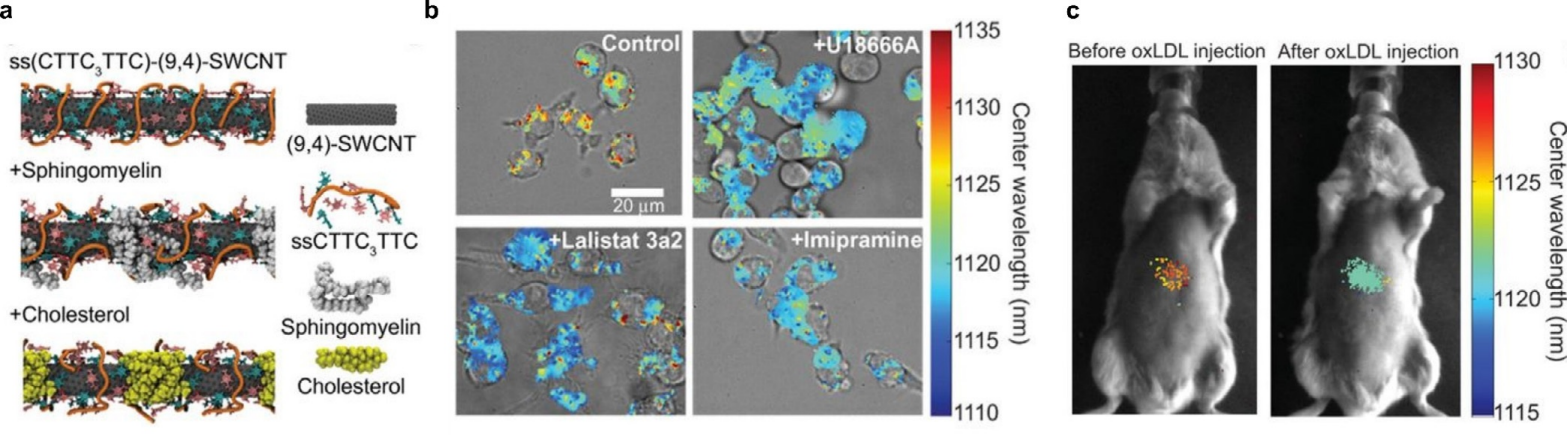
In vivo detection of lipid accumulation. a) Molecular dynamics simulations of ssCTTC_3_TTC‐(9,4)‐SWCNTs that serve as a sensor for the lipids cholesterol and sphingomyelin through a change in the SWCNT emission wavelength. b) Bright‐field images overlaid with hyperspectral NIR images of the sensors in RAW 264.7 macrophages with or without the chemical inhibitors U18666A, Lalistat 3a2, and Imipramine that change the intracellular lipid levels. c) Hyperspectral NIR images of the sensor before and after injection of oxLDL in mice report the in vivo lipid status. Reprinted from Ref. [149] with permission.

The second approach was based on the screening of several ssDNA‐SWCNT chirality combinations to identify CTTC_3_TTC‐(9,4)‐SWCNTs with the greatest wavelength shift of up to 8 nm in response to lipid accumulations.^[149]^ The emission wavelength at 1125 nm is spectrally separated from the lipid absorption band at 1210 nm, thus facilitating promising in vivo applications. Molecular dynamics calculations led to the assumption that the lipid molecules sphingomyelin and cholesterol bind to the SWCNT surface by hydrophobic interactions, thereby decreasing the water density in the SWCNT environment and leading to the observed blue shift (Figure 13 a). To validate the sensor functionality in live cells, endolysosomal lipid accumulation was induced in macrophage cells with chemical inhibitors to mimic different lipid phenotypes. After sensor incubation, a blue‐shift was observed for all sensors within the drug‐treated cells compared to the control (Figure 13 b). For in vivo applications, two mouse models were used with lipid accumulation within the organelles of many cell types, for example, in Kupffer cells. By intravenously injecting the SWCNT‐based sensors, a rapid decrease of the SWCNT fluorescence and consequently removal of SWCNTs from all parts of the mice except for the liver were observed. The sensors were also able to report uptake and endolysosomal lipid accumulation of oxidized low‐density lipoprotein (oxLDL; Figure 13 c). Therefore, these types of sensors provide novel insights into the complexity of lipid metabolism and related health states.

#### 3.2.5 Proteins

Proteins are one of the major biomacromolecules. Consequently, the study of protein–protein interactions helps to understand the function of proteins or to find novel drugs.^[150]^ As discussed above, the detection of proteins with SWCNTs is either based on attaching a known natural recognition element[^21^, ^64^, ^65^, ^102^, ^103^, ^104^, ^105^, ^106^, ^151^] or a synthetic heteropolymer to the SWCNT surface (Figure 3).[^71^, ^101^, ^152^, ^153^] Such studies go beyond detecting the presence of a whole protein.

**Figure 14:**
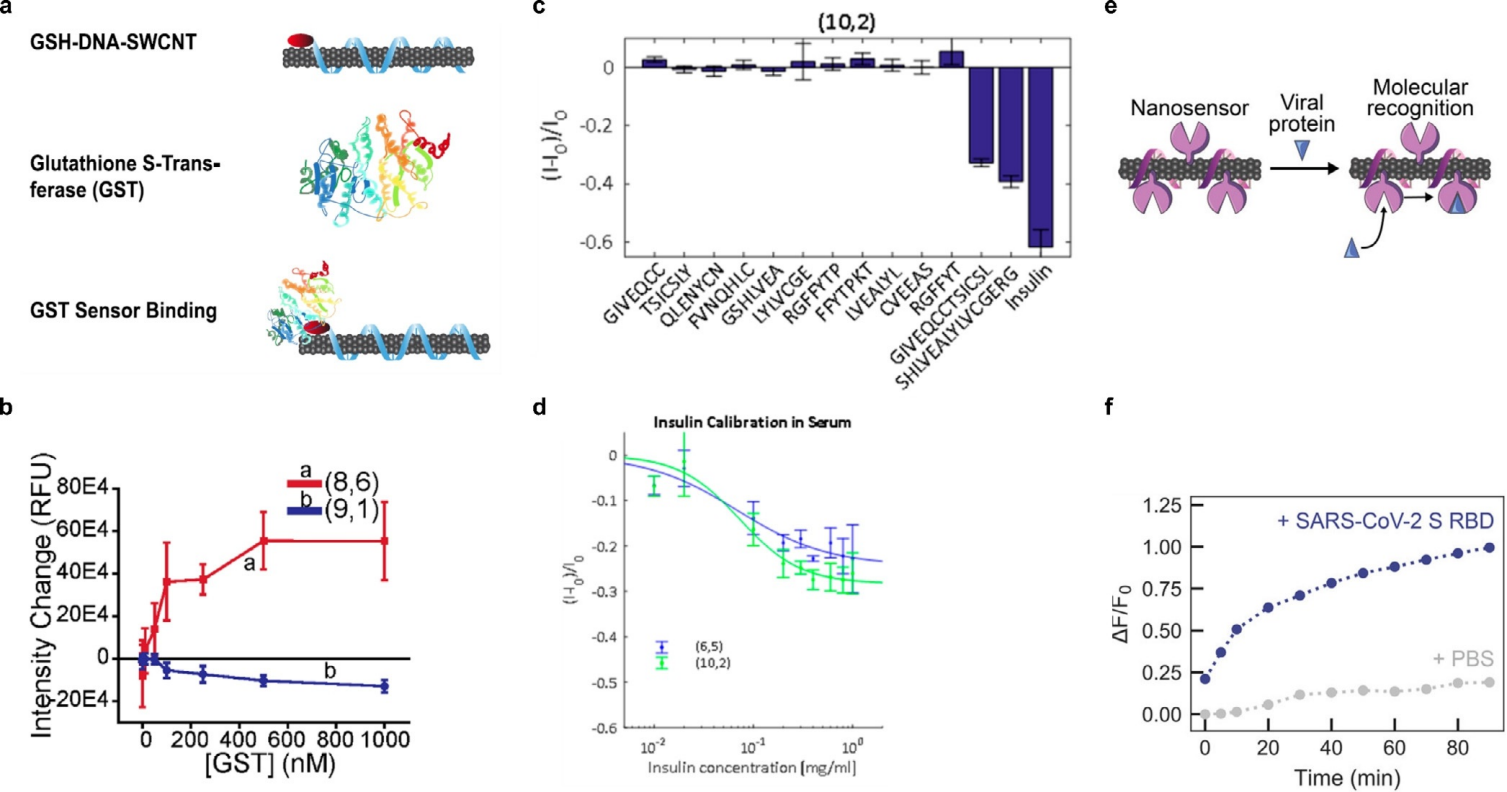
Detection of proteins. a) SWCNT‐based sensor design for the detection of glutathione‐S‐transferase (GST) fusion proteins. b) Monotonic intensity changes of (8,6) and (9,1) sensors with increasing GST concentration. Reprinted from Ref. [154] with permission. Copyright 2020 American Chemical Society. c) Detection of insulin by SWCNTs functionalized with a PEG‐conjugated lipid. Intensity change of the sensor in response to insulin and shorter insulin fragments. d) Insulin calibration curve in serum. Reprinted from Ref. [101] with permission. Copyright 2018 American Chemical Society. e) Detection of the SARS‐CoV‐2 spike protein. ACE2‐(GT)_6_‐SWCNTs interact with the SARS‐CoV‐2 spike protein receptor‐binding domain (S RBD). f) Time‐dependent change in the relative fluorescence intensity of the sensor in response to the viral protein S RBD. Reprinted from Ref. [157] with permission. Copyright 2021 American Chemical Society.

Williams et al. developed the first label‐free optical sensor for the detection of glutathione‐S‐transferase (GST) fusion proteins based on glutathione‐(TAT)_6_‐SWCNTs (GSH‐DNA‐SWCNTs; Figure 14 a).^[154]^ The system used distinct differences in the emission wavelength and intensity of SWCNT chiralities in response to GST and, consequently, the ratiometric intensity of two chiralities (8,6)/(9,1) (Figure 14 b). The functionality of this sensor in response to four different GST fusion proteins was tested, which all showed a similar ratiometric response and a LOD in the low nanomolar regime. This approach has potential for the tracking of protein expressions in real time.

The detection of insulin is essential for diabetes as it controls the glucose levels in blood. The first optical SWCNT‐based sensor approach for detecting insulin was realized in 2010 by Cha et al.^[155]^ They used an insulin‐binding aptamer, which showed a highly specific and sensitive decrease in the fluorescence intensity on forming a guanine quadruplex. Furthermore, they incorporated these sensors in a collagen extracellular matrix and demonstrated sensor reversibility with enzymatic proteolysis and the detection of insulin secreted by pancreatic β‐cells.^[156]^

Recently, insulin detection was enabled by screening a library of PEG‐conjugated lipids. It was found that the C_16_‐PEG(2000 Da)‐Ceramide‐SWCNT complex causes a significant decrease in the fluorescence intensity in the presence of insulin compared to other relevant proteins present in human whole blood.^[101]^ Additionally, nonspecific recognition mechanisms such as hydrophobicity or molecular weight were ruled out. For example, the sensor response to shorter insulin fragments was measured, however no correlation to the molecular weight was observed (Figure 14 c). The analysis of insulin fragments was further supported by enthalpy measurements that showed no affinity of the PEG‐conjugated lipid itself, which highlights that the exact CoPhMoRe phase bound to the SWCNT is responsible for the insulin binding. In more complex environments, such as serum, the sensor affinity was lower but still sensitive enough (Figure 14 d).

The application of sensors in complex biological samples is often challenging. The spontaneous adsorption of proteins onto all kind of materials changes the actual corona structure and might affect sensing. To improve the performance of sensors in those protein‐rich environments, a fundamental understanding of the interaction between sensors and their biological environment is necessary. Pinals et al. addressed this issue by studying the protein corona formation on (GT)_15_‐SWCNTs in cerebrospinal fluid and blood plasma by mass spectroscopy.^[98]^

Their results showed strong binding to fibrinogen and other proteins involved in blood clotting, lipid transport, and complement activation. The identification of interactions responsible for the formation of protein corona revealed that the outer corona formation can be reduced by optimizing the electrostatic interactions through the sensor design and dynamic flow conditions (e.g. with lateral flow assays or microfluidic systems), while entropic calculations must be considered for the inner corona. This study highlights the urgent need to investigate sensors not only in simple buffers but biologically complex environments. Most recently, Ehrlich et al. developed a complementary approach using an insulin aptamer (found within the natural insulin gene promoter) functionalized to the SWCNT surface through a (AT)_15_ ssDNA anchor sequence.^[158]^ In contrast to the synthetic PEGylated‐lipid, which has no prior affinity to insulin, this aptamer possesses a known affinity to insulin. However, the observed sensitivity was lower than the previous approach.

Most of the SWCNT‐based sensors that have been discussed so far were measured in solution. The immobilization of SWCNTs on different porous paper matrices, for example, nitrocellulose, has several advantages for a robust assay.^[116]^ Paper‐based immobilization enabled analyte detection within non‐aqueous solvents such as edible oil, which was previously not possible. To further extend this system the authors used wax to pattern hydrophobic regions onto the paper to create a multiplexed one‐dimensional sensor barcode consisting of different ssDNA‐wrapped SWCNTs.

Another important class in biomedical diagnostics are antibodies. The detection of immunoglobulin G (IgG) with SWCNTs was realized by using chitosan‐wrapped SWCNT noncovalently modified with immunoglobulin‐binding proteins.[^104^, ^151^] Recently, Kozawa et al. designed a flexible fiber optic interface coupled to nanosensors that was capable of detecting the aggregation status of human IgG by reporting the relative fraction of monomers and dimer aggregates with sizes of 5.6 and 9.6 nm.^[159]^ For this purpose, the SWCNT‐based sensors were incorporated into a hydrogel (HG) and attached to the end of a fiber waveguide. Proteins are also part of pathogens and consequently disease markers. Pinals et al. developed a SWCNT‐based sensor which is functionalized with the angiotensin‐converting enzyme 2 (ACE2), a host protein which shows a high binding affinity for the SARS‐CoV‐2 spike protein (Figure 14 e).^[157]^ A twofold NIR fluorescence increase was detected 90 min after the addition of the purified spike protein (Figure 14 f). Passivation with a hydrophilic polymer was used to enable detection of the spike protein in saliva and viral transport medium.

However, antibodies are not always available for all targets. In addition, the development of new recognition units can be expensive and tedious, which is why new approaches are directed to the development of multiplexed sensor arrays[^146^, ^151^] to overcome the limited selectivity of existing single sensors. Recently, Yaari et al. developed a SWCNT solution‐based sensor platform to detect multiple gynecologic cancer biomarkers in uterine lavage samples.^[160]^ The array consisted of eleven different ssDNA‐SWCNT sensors, and the optical change in the intensity and wavelength was extracted for twelve chiralities present in the sample, which resulted in 132 individual ssDNA‐SWCNT complexes. With machine learning algorithms a classification accuracy (F1 score) of 0.95 was achieved. With retraining, this sensor platform may not be limited to the detection of cancer biomarkers. The large variety of possible SWCNT chiralities in combination with unlimited SWCNT wrappings opens possibilities to meet the rising demand of new recognition strategies.

#### 3.2.6 Sugars

Sugars are important building blocks and metabolites. Glucose, in particular, is a major target, for which continuous monitoring of the glucose level in blood is desired. SWCNT‐based sensing ranges from the use of glucose‐specific enzymes[^24^, ^161^, ^162^] or proteins^[108]^ to the first affinity sensor based on the competitive binding between glucose and its polymer dextran.^[11]^ Although improvements were made, the first approaches suffered from limited reversibility and/or physiological detection range. One sensor that meets the requirements is based on the functionalization of SWCNTs with glucose oxidase (GOX), a glucose‐specific enzyme.^[161]^ The addition of glucose causes an increase in fluorescence emission. The proposed mechanism is based on SWCNT fluorescence being quenched by defect sites on the SWCNT surface, which are hole‐doped through oxygen adsorption. The addition of glucose causes an oxidation of the GOX wrapping, which behaves as an electron donor and passivates the oxygenated sites of the SWCNT, thereby resulting in a fluorescence increase. This effect is reversible by removing the glucose. The sensor showed responses to five other tested saccharides, but with the highest response to glucose.

Another recognition element for saccharides is phenylboronic acids, which have been used to functionalize SWCNTs noncovalently for the detection of sugars.^[68]^ Recently, covalent aryl‐boronic acid defects were also incorporated in SWCNTs.^[81]^ Upon interaction with fructose and glucose, these sensors decreased in fluorescence intensity and the E_11_^*^ signal shifted, which can be used for spectrally encoded sensing.

#### 3.2.7 DNA/RNA

One of the most abundant and important types of biomacromolecules are nucleic acids that store and process genetic information. Using a construct of a complementary capture sequence connected to a (GT)_15_‐sequence serving as an anchor, thus providing colloidal stability, SWCNTs were recently used to detect hybridization events of microRNA and other oligonucleotides directly in serum, urine, and in mice in vivo.^[163]^ Upon the addition of complementary nucleic acids, a specific blue‐shift for different chiralities upon hybridization was observed. Additionally, the sensor response was reversible through toehold‐mediated strand displacement and the sensors possessed a LOD in the picomolar range.

**Figure 15:**
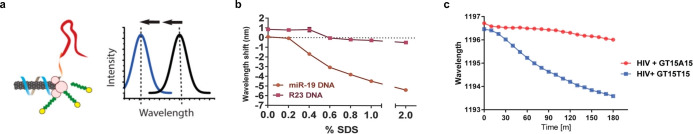
Detection of viral (HIV) RNA. a) Detection of viral RNA using a HIV lentivirus model. The virus is denatured by SDS, which liberates the RNA genome. It hybridizes to the complementary ssDNA on the SWCNT‐based sensor and increases the free surface area, which is then occupied by serum proteins and causes a blue‐shift of the spectrum. b) Wavelength shift in response to the target miR‐19 DNA or control R23 DNA in bovine serum albumin with various SDS concentrations. c) Kinetics of the response of sensors with complementary ((GT)_15_T_15_) and negative control ((GT)_15_A_15_) ssDNA in the presence of HIV particles, FBS, and 2 % SDS. Reprinted from Ref. [164] with permission. Copyright 2019 American Chemical Society.

Further development of this sensor led to the first SWCNT‐based sensor for the detection of HIV in serum.^[164]^ Harvey et al. discovered that SDS‐denaturized serum proteins lead to an enhanced optical response of the SWCNTs in response to DNA hybridizations. They hypothesized that the addition of SDS ensured both the liberation of the viral RNA genome and the denaturation of the proteins which competitively bind to the freed surface of the sensor. The interaction leads to a blue‐shift in the SWCNT emission (Figure 15 a). This was first shown for hybridization with complementary target miR‐19 DNA compared to control R23 DNA (Figure 15 b). A dose‐dependent enhancement of the blue‐shift occurred in the region of the critical micelle concentration of SDS; thus, the denaturation of proteins by SDS is considered to involve an unfolding process of the tertiary structure of the protein to complete denaturation. As the SDS concentration was increased, the amount of denatured protein absorbed onto the SWCNT surface after hybridization of the DNA increased and saturated at 2 % SDS. For HIV detection, the recognition strategy was based on a sensor consisting of (GT)_15_‐T_15_‐SWCNTs, which hybridize with the polyadenylation elements of HIV RNA (Figure 15 c). In a similar way, a control sensor with the noncomplementary capture sequence, namely (GT)_15_‐A_15_‐DNA, was constructed. The wavelength shifts of both the sensor and negative control in the presence of HIV particles treated with 1 % SDS were recorded over time and and the sensor displaying the complementary capture sequence showed a blue‐shift of around 3 nm after 180 min.

A completely different application of DNA chemistry on SWCNTs was established by Cha et al. Based on the consumption of chemical energy delivered by the RNA molecules, they developed a synthetic motor that transports nanoparticles through the mechanical motion of DNA conformation changes along the SWCNTs.^[165]^ Movements of the motor of over 3 μm with a speed of 1 nm min^−1^ were observed.

#### 3.2.8 Enzymes

So far, the detection of enzymes with SWCNTs has been shown for the characterization of proteases and DNases as well as cellulases and pectinases. The hydrolytic enzyme activity was measured by Kallmyer et al., who used hydrolytic enzyme wrapped SWCNTs that respond to the target enzyme with a fluorescent intensity quenching because of the degradation of the enzyme‐wrapping.^[166]^ Different polymer wrappings consisting of polysaccharides and polypeptides were used to study cellulase, pectinase, and bacterial protease. Most recently, they used this approach to evaluate the enzyme activity in soil using a low‐cost multiplexed and portable fluorimeter able to perform the measurement outside the laboratory only minutes after extraction from the field.^[167]^ As a consequence of the fresh nature of the soil sample, field tests indicated activities an order of magnitude larger than those obtained in benchtop experiments.

Enzymes are also released by microorganisms, which can be used to fingerprint them. Nißler et al. chemically tailored SWCNTs to detect enzymes such as DNases and proteases.^[29]^ For targeting extracellular proteases, SWCNTs were modified with bovine serum albumin (BSA), which serves as an enzymatic substrate, while SWCNTs were functionalized with calf thymus (CT) DNA for reporting DNase I and *S. aureus* nuclease activity. The sensors showed a fluorescence decrease, most likely as a result of decomposition of the BSA surface coating. These SWCNT‐based sensors were further used for the discrimination of bacteria, which are known to alter their chemical environment through the release of signaling molecules, enzymes, and metabolites (see section below).

In contrast, Shumeiko et al. used peptide‐encapsulated SWCNTs, which also responded through a fluorescence decrease upon enzymatic digestion of the SWCNT wrapping.^[168]^ They utilized a low‐cost paper‐based dipstick system, with which they evaluated the trypsin activity in urine samples as a mimic for acute pancreatitis, where abnormal trypsin concentrations are common.

To study the enzyme myeloperoxidase, which is involved in the regulation of inflammation processes, He et al. used a ratiometric system based on graphene oxide (GO) wrapped SC‐SWCNT sensors and GO‐wrapped carboxymethylcellulose (CMC)‐SWCNTs as a reference.^[169]^ GO and SC‐SWCNTs showed an opposed fluorescence signal in response to enzymatic degradation. Whereas the blue fluorescence intensity of GO was increased due to oxidation and degradation of GO leading to the formation of graphene quantum dots, the NIR emission of SWCNTs decreased due to the generation of defects on the SWCNT surface. In contrast, the CMC‐SWCNT reference was almost stable as a result of the better surface protection of the CMC‐wrapping.

#### 3.2.9 Epitopes and Metabolites from Pathogens

**Figure 16:**
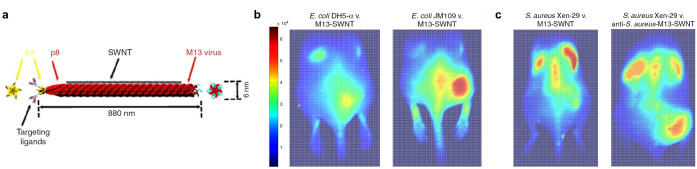
Labeling of bacteria. a) The bacteriophage M13 and its proteins p8 and p3 are used to longitudinally functionalize the SWCNT and to bind a target. b) Fluorescence image of M13‐SWCNTs, intravenously injected into mice infected with *E. coli* strains. The right flank is either infected with F′‐negative *E. coli* DH5‐α strains (left) or the targeted F′‐positive *E. coli* JM109 strains (right). The left flank serves as a control (PBS). c) Fluorescence images of mice infected with *S. aureus* strain Xen‐29. The left mouse received M13‐SWCNTs, while the right mouse received antibody labelled sensors (anti‐*S. aureus*‐M13‐SWNTs). Adapted from Ref. [170] with permission. Copyright 2014 Nature Publishing Group.

Microbial infections are one of the major causes of mortality worldwide. Currently, the limited number of diagnostic methods in combination with increasing antibiotic resistances demonstrate the rising need for the rapid, contactless, and specific detection of pathogens such as bacteria. The optical properties of SWCNTs promise advantages for pathogen detection. Bardhan et al. developed M13 bacteriophage functionalized SWCNTs (M13‐SWNTs, Figure 16 a ), which are able to distinguish F′‐positive and F′‐negative bacterial strains by modulation of the fluorescence intensity.^[170]^ The M13 bacteriophage has a known binding affinity to F′‐positive *E. coli* strains. Therefore, they intramuscularly infected the right flank of living mice with *E. coli* strains, either F′‐negative DH5‐α strains (Figure 16 b , left) or targeted F′‐positive JM109 strains (Figure 16 b, right) and observed a 1.6‐fold intensity increase over the nonspecific DH5‐α strains. Injection of PBS into the right flank served as a control. To extend this SWCNT‐based label to a wider range of bacterial strains lacking F′‐pili, they additionally attached an antibacterial antibody on those M13‐SWCNTs (anti‐*S. aureus*‐M13‐SWNT) through a streptavidin–biotin reaction. This complex specifically detected *S. aureus* intramuscular infections in a mouse model in vivo (3.1‐fold intensity increase over the nontargeted M13‐SWCNTs, Figure 16 c).

**Figure 17:**
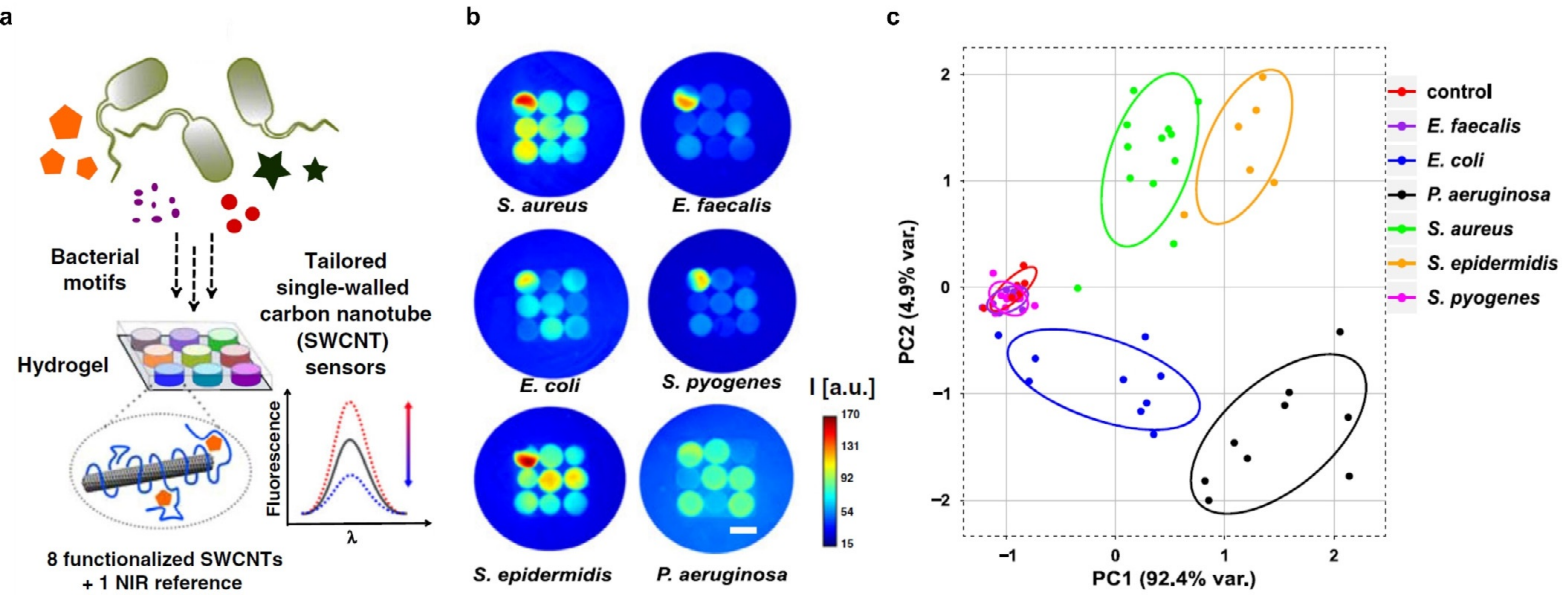
Remote detection of pathogens. a) Eight fluorescent SWCNT‐based nanosensors and one NIR fluorescent reference material are incorporated into a polyethylene glycol hydrogel (HG) array that is remotely monitored in the NIR region. The sensors change their fluorescence signal in response to bacterial metabolites and virulence factors, which are released by bacteria growing on top of this HG. b) The unique NIR fingerprint of the multiplexed sensor array allows important pathogens to be differentiated. c) Principal component analysis (PCA) of the fluorescence fingerprint of all the analyzed strains (72 h). Each point represents one bacterial sample, including clinical isolates from different patients. The clusters show that different bacteria can be identified and distinguished. Control=medium only. Reprinted from Ref. [29] with permission.

This approach is a NIR labeling rather than sensing. In contrast, Nißler et al. created a concept to remotely distinguish six important pathogens using an array of SWCNT‐based sensors:^[29]^ four for specific bacterial target detection based on a rational approach and four generic lower sensitivity sensors together with an invariant reference consisting of NIR fluorescent Egyptian Blue nanosheets (Figure 17 a).^[171]^

To overcome unspecific effects arising from the complex composition of bacterial media, the sensors were incorporated into a hydrogel (HG) array, in which the pore size was varied in accordance to the size of the analyte. For the detection of small molecules (such as siderophores), HGs with a low porosity were used, whereas HGs with a high porosity allowed large enzymes to diffuse to the sensors. The sensors were exposed to clinical isolates of bacteria and the unique change in the fluorescence intensity for each sensor was monitored remotely. Within 24–72 h, a unique fingerprint in response to the tested pathogens was visible (Figure 17 b), which further allowed differentiation of the pathogens by principal component analysis (PCA; Figure 17 c). Besides this spatially encoding, spectral multiplexing was implemented to differentiate *S. aureus* and *P. aeruginosa* by using two monochiral SWCNT‐based sensors with different wavelengths compared to the reference. Moreover, the signal of the sensor could be detected through tissue to a depth of >7 mm, which highlights the potential for biomedical in vivo applications.

### 3.3 Mechanism of Fluorescence Modulation

**Figure 18:**
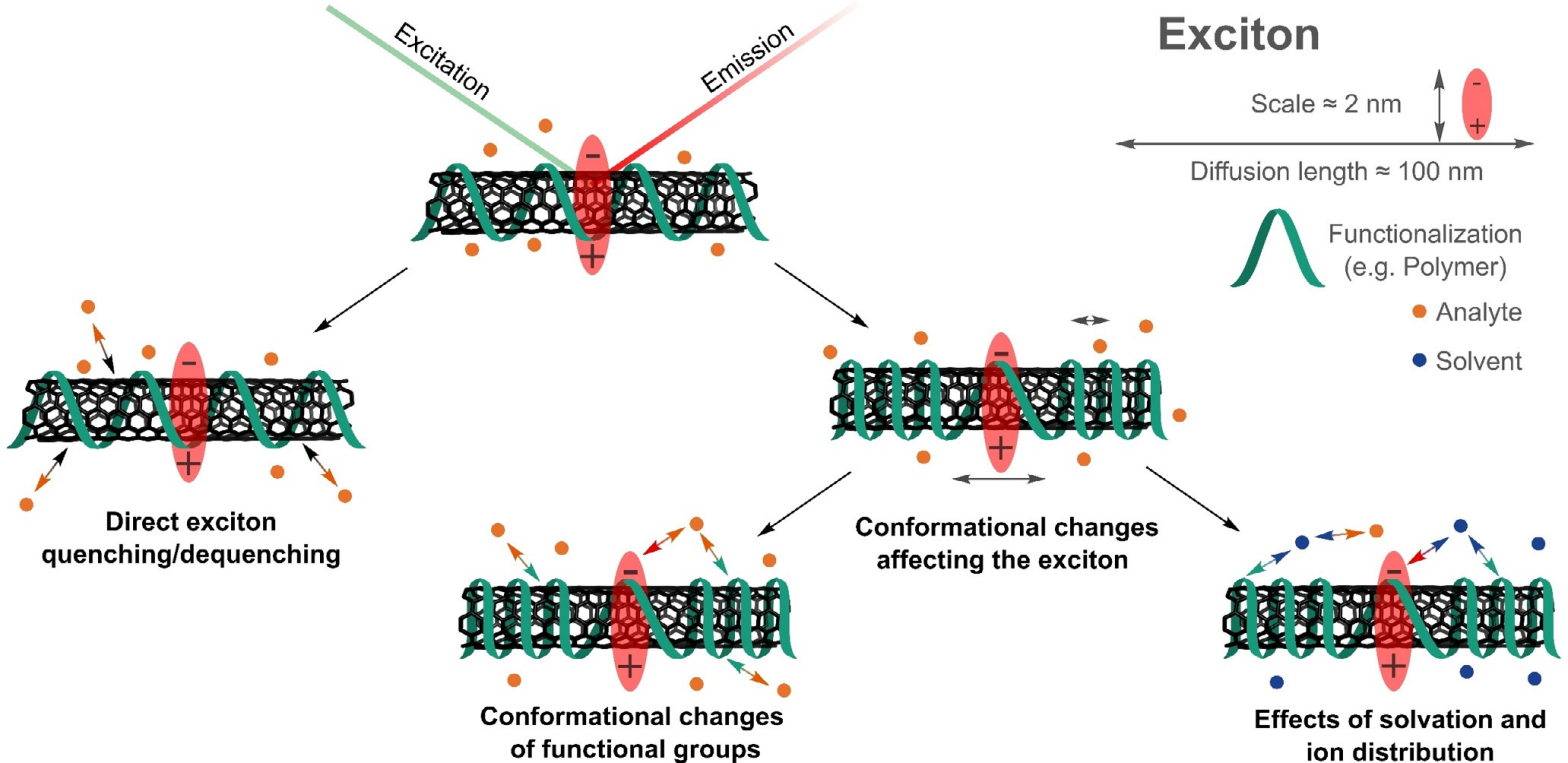
Mechanisms of fluorescence modulation in SWCNT‐based biosensors. Note that the size of the objects and their arrangement is simplified and not to scale.

In SWCNT‐based sensors or probes, the SWCNTs serve as transducer elements that translate chemical changes caused by an analyte in the vicinity of the SWCNT into a fluorescent signal. Thus, SWCNT‐based sensing involves molecular recognition and signal transduction. The precise mechanisms are most likely different for different analytes and different surfaces. However, from the literature one can distinguish several possible generic mechanisms (Figure 18).

#### 3.3.1 Direct Quenching

**Figure 19:**
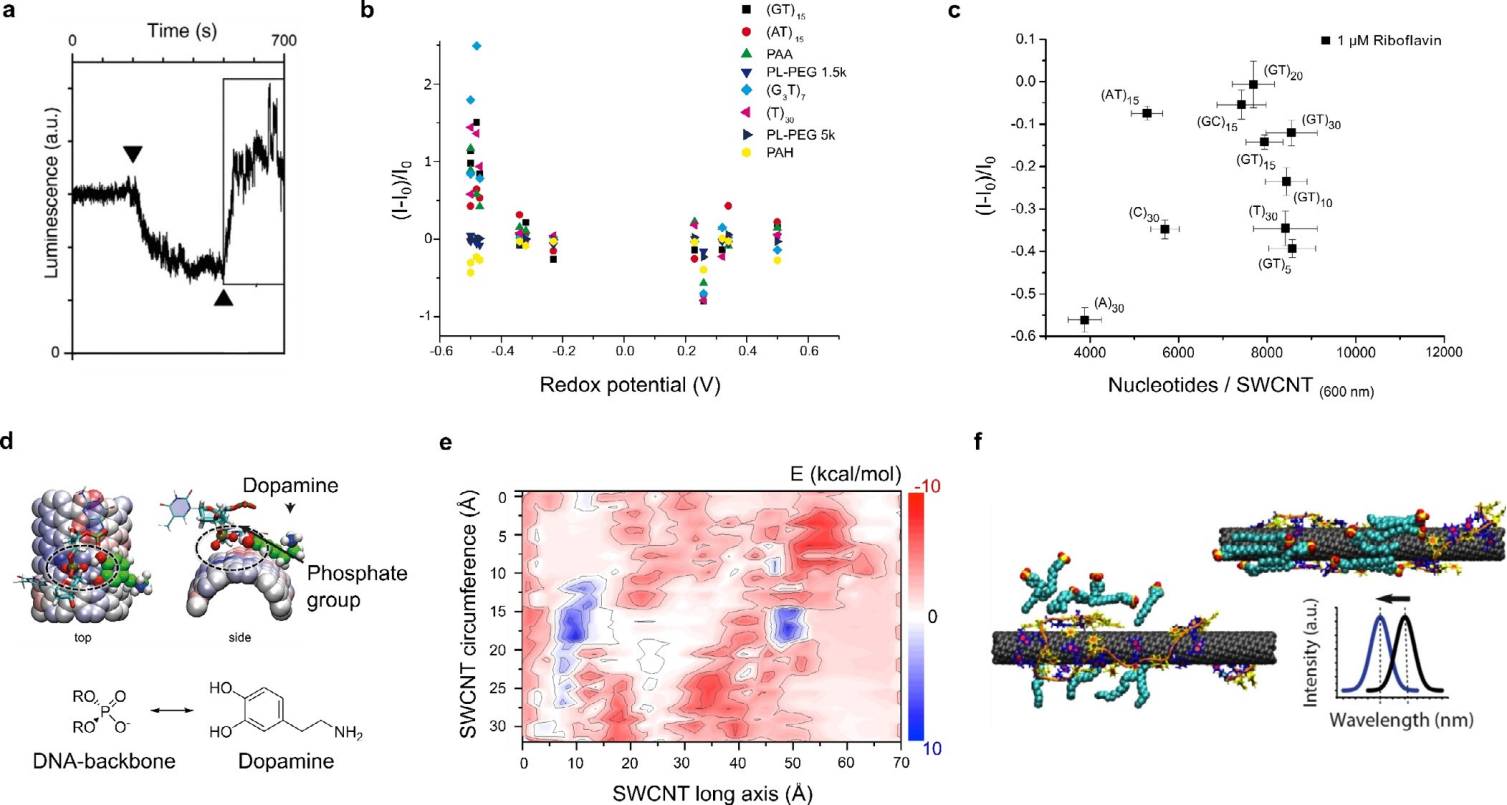
Insights into the mechanism of SWCNT‐based sensors. a) Change in the fluorescence signal of an individual (7,6)‐SWCNT upon successive addition of acid (▾) and base (▴). Luminescence recovery is observed after base addition, which indicates direct quenching by protons. Adapted from Ref. [47] with permission. b) Correlation between the fluorescence response of different SWCNT/polymer conjugates for different analytes (replicate symbols), such as dopamine and ascorbic acid, and the redox potential. The spread along the *y* axis indicates that the redox potential alone cannot explain the fluorescence change. Adapted from Ref. [63] with permission. Copyright 2016 American Chemical Society. c) There is also no simple correlation between the fluorescence response and surface coverage, shown here for ssDNA‐functionalized SWCNTs and the response to riboflavin. Adapted from Ref. [176] with permission. Copyright 2019 American Chemical Society. d) Mode of interaction between dopamine and DNA‐functionalized SWCNTs. The hydroxy groups of dopamine interact with the phosphate groups of the DNA backbone, which pulls them closer to the SWCNT surface. Most likely, solvation effects play an important role. e) Corresponding potential landscape on a SWCNT functionalized with (GT)_15_ DNA. The blue regions with changed potential colocalize with the dopamine interactions site and affect the exciton fate. In this particular case, the fluorescence quantum yield increases. Adapted from Ref. [100] with permission. f) Wavelength shift in response to an interaction between ssDNA‐suspended SWCNTs with SDBS, a surfactant. The hybridization of ssDNA is influenced by the salt concentration and changes the surface accessibility of the SWCNT. Reprinted from Ref. [177] with permission. Copyright 2018 American Chemical Society.

Direct quenching, that is, the decrease of fluorescence, is caused by adsorption of the analyte onto the SWCNT surface. Changes in the pH value caused by the addition of an acid can cause protonation of the SWCNT sidewall and result in direct, reversible quenching of the SWCNT fluorescence (Figure 19 a). Mechanistically, this can be explained by the injection of an electron hole into the π‐system near the protonation site.^[47]^ Excitons encountering such an electron hole will be quenched through a nonradiative Auger process.^[172]^ Furthermore, electron transfer between the valence band of 3,4‐diaminophenyldextran‐functionalized SWCNTs and the lowest unoccupied molecular orbital (LUMO) of nitrogen oxide results in rapid, reversible quenching of the SWCNT fluorescence.^[124]^ Both of these interactions take part in the vicinity of the SWCNT and, consequently, solvent effects should play a large role.

#### 3.3.2 Impact of Conformational Changes and Solvation

Within the dimensions of the exciton, the fluorescence of SWCNTs is very sensitive to the surrounding environment. To study the contributions of the solvent to the fluorescence of SWCNTs, fluorescence spectra were analyzed in different dielectric environments.[^173^, ^174^] By using the solvatochromic shifts, a semiempirical scaling model was developed that linked optical with structural parameters and suggested an inverse dependence of exciton polarizability on the diameter and the square of the transition energy.^[173]^ In nonpolar solvents, the solvatochromic modulation of the fluorescence intensity becomes more pronounced for larger diameters.^[174]^ Changes in the solvatochromic shift, on the other hand, were more pronounced for SWCNTs with smaller diameters.^[174]^

In general, the displacement of H_2_O or DNA from the surface of SWCNTs by surfactants leads to a strong blue‐shift and increase in the fluorescence intensity. Interestingly, the change in the fluorescence characteristics of pristine SWCNTs and DNA‐coated SWCNTs immobilized in gel are highly similar. This suggests that considerable portions of the nanotube surface are exposed to H_2_O.^[175]^ However, to date, a local model of solvatochromism that accounts for the nonhomogeneous structure around SWCNTs is missing.

One of the best understood systems is the recognition of small molecules by DNA‐functionalized SWCNTs. Here, the mechanism of sensors for catecholamine neurotransmitters such as dopamine have been studied in greater detail (Figure 19 d,e).

As these molecules are redox‐active, they could reduce or oxidize either the SWCNT or the surrounding organic phase, thereby affecting the fluorescent properties. To study this potential mechanism, the redox potential and the fluorescent response of certain analytes were correlated. This study showed that molecules with a negative redox potential are more likely to increase the SWCNT fluorescence (Figure 19 b).^[63]^ However, as molecules of the same redox potential can induce drastically different fluorescence responses, the redox potential alone cannot account for the fluorescence changes observed.^[63]^ Likewise, fluorescent responses of ssDNA‐functionalized SWCNTs to dopamine and riboflavin cannot be correlated to the amount of adsorbed nucleotides/DNA molecules on the SWCNT surface (Figure 19 c), which suggests that more complex conformational changes are responsible for the change in the SWCNT fluorescence.^[176]^ Molecular dynamics simulations showed that a stacking of dopamine with DNA‐functionalized SWCNTs leads to interactions between the phosphate backbone of the DNA as well as the hydroxy and amine groups of dopamine (Figure 19 d,e).^[100]^ As a result of this interaction, the phosphate backbone moves toward the SWCNT surface and the electrostatic potential at the SWCNT surface changes (Figure 19 d,e).^[100]^ It is also known that the diffusion coefficient of the excitons in surfactant‐containing systems changes with the surfactant identity and, furthermore, correlates with the fluorescence intensity.^[48]^

As pristine SWCNTs do not show any fluorescence response to dopamine^[100]^ and differently functionalized SWCNTs display different affinities to neurotransmitters,^[118]^ it follows that the organic phase (DNA) governs both the sensitivity as well as selectivity for this neurotransmitter.^[118]^ The used biopolymers are typically charged and, consequently, electrostatic interactions play an integral part for biosensing.^[177]^ It has been shown that the presence of certain salts alters the conformation of the DNA wrapping on the SWCNT[^56^, ^57^] and decreases the electrostatic repulsions between equally charged molecules.^[177]^ To reduce ion‐induced fluorescence effects, the flexibility of the DNA can be altered by using xeno nucleic acids.^[57]^ A good example of the influence of electrostatic repulsion and screening effects is the increased SWCNT surface accessibility at higher salt concentrations (Figure 19 f).^[177]^ In a surfactant‐containing system, the exposed surface is covered by the surfactant, which causes a blue‐shift of the NIR fluorescence, again demonstrating that the dynamics of the organic phase around the SWCNT is crucial for the sensing mechanism.^[177]^

By modulating the exposed surface area, it is furthermore possible to tune surfactant‐suspended SWCNTs to respond to different bio‐analytes.^[137]^ Together, these findings indicate that fluorescence is modulated by the precise 3D arrangement of the molecules, ions, and water molecules in the vicinity of the nanotube.

#### 3.3.3 Exciton Decay and Defects

As described above, the introduction of certain sp^3^ defects into the carbon lattice of SWCNTs can increase the NIR fluorescence through the trapping of excitons.[^53^, ^74^] The defined introduction of quantum defects, thus, provides a way to perturb the exciton decay and elucidate the involved processes that ultimately affect the fluorescent response. As the NIR fluorescent response of SWCNT‐based sensors to dopamine in H_2_O and D_2_O did not show major differences, electronic‐to‐vibrational energy transfer (EVET)^[178]^ seems not to be a main factor in (dopamine) sensing.^[87]^ In contrast, the correlation between the length of the SWCNTs and their fluorescent response seems to indicate that quenching at the ends plays a role. However, the fluorescence response was independent of the variation of defect density.^[87]^ Together with the finding that a small number of quantum defects reverse the fluorescent response of DNA‐functionalized SWCNTs to dopamine (Figure 6 d), it follows that multiple rate constants are affected by the analyte.^[87]^ Computationally, the experiments were best explained by a three rate constant model (3RC) that includes a decrease in the nonradiative decay from the E_11_ state (*k*_nr_), an increase in the exciton diffusion constant (D_e_), and an increase in the nonradiative decay constant from the E_11_^*^ (*k*_nr_^*^) caused by dopamine.^[87]^ Together, these insights highlight the complex interplay between photophysics and molecular recognition as well as new avenues to tailor sensing using defects.

### 3.4 Considerations on Kinetics and Imaging

The processes related to sensing happen on a certain time scale. How fast an analyte binds or dissociates from a sensor is determined by its kinetics. Additionally, the optical signal is detected in setups that determine aspects such as spatial resolution or imaging speed. In the following sections we discuss how these hallmarks of fluorescence sensors affect their performance.

#### 3.4.1 Kinetics of Sensors and Impact on Spatiotemporal Resolution

Chemical imaging with many SWCNT‐based sensors at one time is a highly effective strategy to gain chemical information from a sample with outstanding spatial and temporal resolution.^[179]^ To understand how the collective image of such an array of sensors reflects the concentration of an analyte, Meyer et al. used stochastic Monte Carlo simulations to study the kinetic requirements for spatiotemporal chemical imaging with nanoscale sensors such as SWCNTs.^[180]^

**Figure 20:**
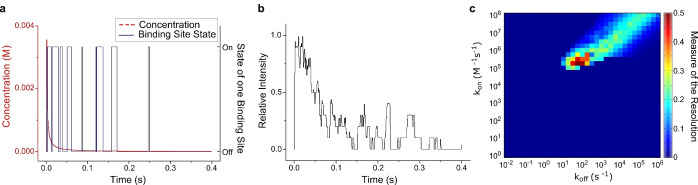
Kinetic requirements of sensors for spatiotemporal imaging. a) A stochastic‐kinetic simulation allows the binding states (blue) to be predicted and consequently the fluorescence traces of single sensors in response to a changing concentration profile (red), for example, release of molecules from a cell. b) Relative intensity response of a single sensor over time based on the concentration profile shown in (a) and the assumption of 10 binding sites. c) Spatial and temporal resolution phase diagram as a function of rate constants. The red regions show combinations of rate constants that allow the detection of the biological events (here, release of molecules from a cell). Reprinted from Ref. [180] with permission. Copyright 2017 American Chemical Society.

The subject of the simulation was a nanosensor array being exposed to a changing concentration gradient of an analyte. To predict the image one gains from many sensors, single sensor responses were first simulated. In a typical scenario, the time‐dependent concentration/diffusion profile of a dynamic process, such as release of signaling molecules (e.g. neurotransmitters) from cells and the stochastic binding site state of the sensors for certain rate constants, was simulated to calculate the expected fluorescence change of single sensors (Figure 20 a,b). The overall image was then calculated considering the individual fluorescence emission point spread functions and technical considerations, such as the frame rate of the camera. This simulation allows the prediction of how the rate constants of a sensor (*k*_on_, *k*_off_) and other factors affect the spatiotemporal resolution, for example, to resolve fast concentration changes such as neurotransmitter release from cells. It can serve as guiding principle for the chemical design but also for the interpretation of signals from a given biological problem. Phase diagrams (Figure 20 c) indicated that the senors need a surprisingly low affinity (*K*_d_=100 μm) to resolve fast processes.

#### 3.4.2 Ratiometric Detection

So far, optical biosensors based on SWCNTs have mainly been fabricated from mixtures of multiple chiralities. This leads to a large spectral overlap in the fluorescence emission, which complicates multiplexing and reduces sensitivity.

Advances in the separation and functionalization of SWCNTs have recently enabled the sole use of SWCNTs emitting below 1000 nm together with low‐cost silicon‐based detectors[^145^, ^181^] for the development of ratiometric sensors,[^20^, ^29^, ^30^, ^32^, ^33^, ^112^, ^121^, ^154^, ^169^] in which two or more distinct NIR signals are detected simultaneously. Here, one SWCNT chirality is typically not responsive to the analyte and acts as a reference. Besides the fact that single chirality SWCNTs lead to six‐ to tenfold higher fluorescence intensities compared to multichirality SWCNT mixtures at the same concentration,[^49^, ^182^] ratiometric approaches are more stable to external noise. Despite the clear advantages, the implementation was not possible for a long time because of difficulties in gaining chirality‐pure SWCNTs.

Although progress was made in the synthesis of chirality‐enriched SWCNT samples,^[183]^ only a few chirality‐enriched samples are commercially available. Different separation and purification methods have been developed. They range from density gradient centrifugation,^[184]^ gel chromatography,^[185]^ ion‐exchange chromatography,^[55]^ and aqueous two‐phase extraction^[186]^ to wrappings of special macromolecules that preferably solubilize certain chiralities.[^22^, ^187^] What they all have in common is that the resulting pure chiralities are solubilized in certain polymers or surfactants. However, for a biosensing application it is necessary to tailor the surface chemistry. Therefore, straightforward processes are required that yield chirality‐pure SWCNTs with tunable functionalization.^[121]^

**Figure 21:**
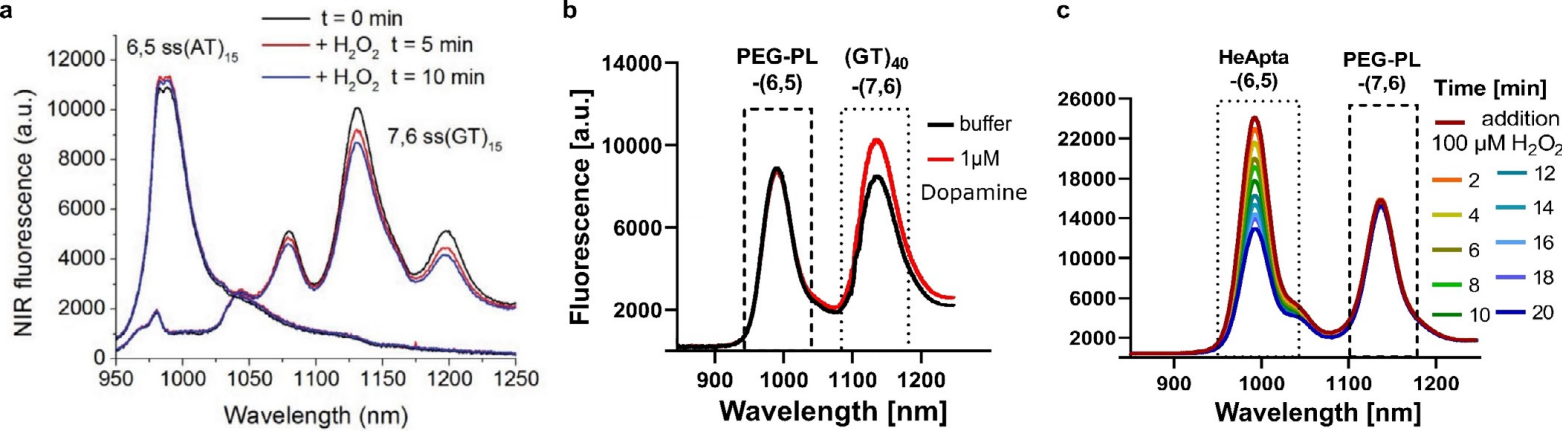
Ratiometric sensing. a) NIR fluorescence of (GT)_15_‐(7,6)‐SWCNTs in response to H_2_O_2_ compared to the nearly invariant (AT)_15_‐(6,5)‐SWCNT reference at different time points. Reprinted from Ref. [20] with permission. b,c) Ratiometric sensing of dopamine and H_2_O_2_ using PEG‐PL as a reference and (GT)_40_‐SWCNTs or SWCNTs functionalized with the hemin‐binding aptamer (HeApta). The reference sensor can be made from different colors of SWCNTs ((6,5) or (7,6) chirality). Adapted from Ref. [49] with permission. Copyright 2021 American Chemical Society.

The first ratiometric SWCNT‐based sensor was demonstrated by Giraldo et al., who integrated into the leaves of plants two sensors: one for the detection of H_2_O_2_ and one for NO.^[20]^ The sensor system was based on the ratio of the distinct emission bands of two chirality species (Figure 21 a). Whereas the (GT)_15_‐(7,6)‐SWCNT‐based sensor was quenched by 20 % within 10  min in the presence of 100 μm H_2_O_2_, the reference sensor remained mostly invariant to the analyte. The overall sensor response was similar to in vitro tests.

**Figure 22:**
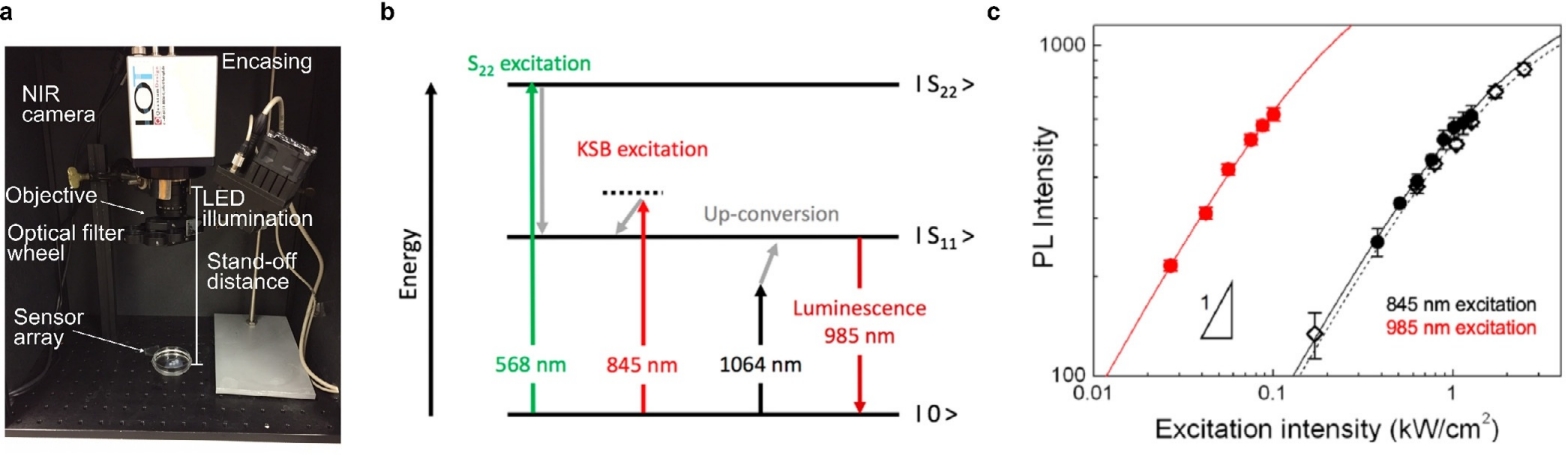
Imaging and excitation approaches. a) Customized portable NIR standoff device enables remote detection (25 cm) by fluorescence sensors, for example, of bacteria.[^29^, ^171^, ^188^] Adapted from Ref. [29] with permission. b) Different possible excitation pathways for fluorescent (6,5)‐SWCNTs. Reprinted from Ref. [189] with permission. Copyright 2018 American Chemical Society. c) Comparison of fluorescence brightness of *p*‐nitroaryl SWCNTs with introduced sp^3^ defects wrapped with PEG‐PL (solid circles) on excitation of the KSB (845 nm) or E_11_ (985 nm) with PEG‐PL‐SWCNTs (open diamonds, KSB excitation). Reprinted from Ref. [190] with permission.

Most recently, Nißler et al. combined the isolation of specific SWCNTs and their subsequent functionalization for the detection of neurotransmitters and other small molecules.^[49]^ They used aqueous two‐phase extraction to obtain chirality‐pure (6,5)‐, (7,5)‐, (9,4)‐, and (7,6)‐SWCNTs and applied a surface exchange through dialysis to remove the DOC wrapping of the SWCNTs and replace it with specific ssDNA sequences or aptamers. This approach enabled the fabrication of ratiometric sensors, for example, for the detection of dopamine and H_2_O_2_ (Figure 22 b,c). For these sensors, SWCNTs were functionalized either with a (GT)_40_ sequence or, in the case of H_2_O_2_ detection, with an aptamer (hemin binding aptamer, HeApta) that binds the protoporphyrin hemin and catalyzes the decomposition of H_2_O_2_. PEG‐PL‐functionalized SWCNTs of another chirality served as an invariant reference. These examples show the potential of ratiometric or multiplexed sensing and given the large wavelength range of SWCNT fluorescence, there are plenty of opportunities for advanced sensing schemes.

#### 3.4.3 Remote Imaging and Alternative Excitation Pathways

Remote imaging—the spatial separation of molecular sensors and detectors—is particularly beneficial to observe biochemical processes non‐invasively, for example, in biomanufacturing or in vivo. As only a small portion of the emitted light is captured by a camera within a certain distance to the sensors, remote detection requires either bright fluorophores or cameras with a high quantum yield in the spectral window of the fluorophore emission.

An example for remote imaging are NIR fluorescent nanosheets, which were remotely detected in a customized portable setup.[^171^, ^188^] Due to the high brightness and emission closer to the visible range (910 nm), a low‐cost CMOS camera (Complementary Metal Oxide Semiconductor, Si based), which typically has a low quantum efficiency of around 5 % in the NIR region, could be used here.

Additionally, this setup was modified for SWCNT detection by using an NIR‐sensitive InGaAs camera, a white‐light LED, and corresponding filters for wavelength‐specific excitation and detection of emission (Figure 22 a). Up to now, remote imaging of fluorescent SWCNT‐based sensors has been used for the identification of bacteria^[29]^ and monitoring of plant health,[^30^, ^31^, ^32^, ^148^] for which resolutions in the (sub‐)millimeter range were achieved with standoff distances of up to 1 m.

Apart from exploiting the decreasing sensitivity of Si‐detectors in the NIR, efforts have been made to promote NIR‐fluorescent transducers using straightforward designs for inexpensive NIR fluorimeters.^[167]^ Instead of using high‐cost InGaAs photodiode arrays, single InGaAs diodes were combined with a motorized stage controlled with an open source programming language. The robustness of these devices outside of the laboratory was demonstrated in a high‐throughput format with field‐side measurements of soil samples. Future developments might also show remote detection using pure (6,4)‐SWCNTs with Si‐based cameras and also versatile use as smart surfaces, for example, for monitoring contamination with pathogens, such as bacteria, on medical surfaces or even implants.

Fluorophore brightness is not the only performance‐related factor when measurements in live biological tissues are carried out. Fluorophore stability as well as wavelength‐dependent tissue scattering, absorption, and the background originating from autofluoresence must also be considered. From this point of view, SWCNTs have excellent photophysical properties.

However, imaging at the single molecule level is always accompanied by considerations to achieve a high signal to noise ratio. Long‐term single SWCNT imaging has, in particular, become established for (6,5)‐SWCNTs in living cells^[191]^ and brain tissue.^[192]^

Although the second‐order excitonic transition, E_22_ (S_22_), is typically used for excitation, alternative excitation pathways such as the K‐momentum exciton‐phonon sideband (KSB) excitation^[193]^ and up‐conversion excitation^[194]^ have been successfully demonstrated, but excitation efficiency, photostability, as well as absorption and scattering of molecules in tissue differ depending on the excitation wavelength. Danné et al. evaluated the different excitation options (Figure 22 b) for optimal single (6,5)‐SWCNT imaging.^[189]^ Here, E_22_ excitation of PEG‐PL SWCNTs was found to be four times more efficient than KSB excitation and an order of magnitude more efficient than 1064 nm up‐conversion excitation, while the signal to noise ratio was more than five times higher for KSB and up‐conversion excitation. However, the excitation at the E_22_ transition is not ideal due to limited tissue penetration depth and autofluorescence. In addition, simulations to quantify the impact of tissue absorption showed that a higher temperature rise of the tissue induced by up‐conversion excitation might be an issue, thus suggesting that KSB excitation is the best choice when considering all the factors. Nevertheless, it still requires relatively high excitation doses in the kW cm^−2^ regime.

Recently, single SWCNT imaging was performed in brain tissue in vivo by using ultralow excitation doses of 0.1 kW cm^−2^ (Figure 22 c).^[190]^ To achieve this, sp^3^ defects were introduced into (6,5)‐SWCNTs, which lead to a fluorescence emission at E_11_^*^ (1160 nm) when exciting at the first‐order excitonic transition E_11_ (985 nm). This approach is beneficial as a result of excitation in the NIR window, but also because of the increased brightness by channeling free excitons to defect sites and subsequent E_11_^*^ emission.

Another way to both excite and detect in the NIR region is the use of multiphoton microscopy. This technique relies on the nonlinear excitation of at least two photons and is optimal for in vivo tissue applications when using NIR radiation. Del Bonis‐O′Donnell et al. demonstrated two‐photon 1560 nm excitation of dopamine‐sensitive SWCNT‐based sensors, which showed only 4 % scattering (one‐photon excitation: 42 % scattering).^[195]^ However, the frame rate is significantly reduced for single sensor imaging compared to wide‐field one‐photon excitation.^[196]^

#### 3.4.4 Hyperspectral and Spinning Disc Microscopy

**Figure 23:**
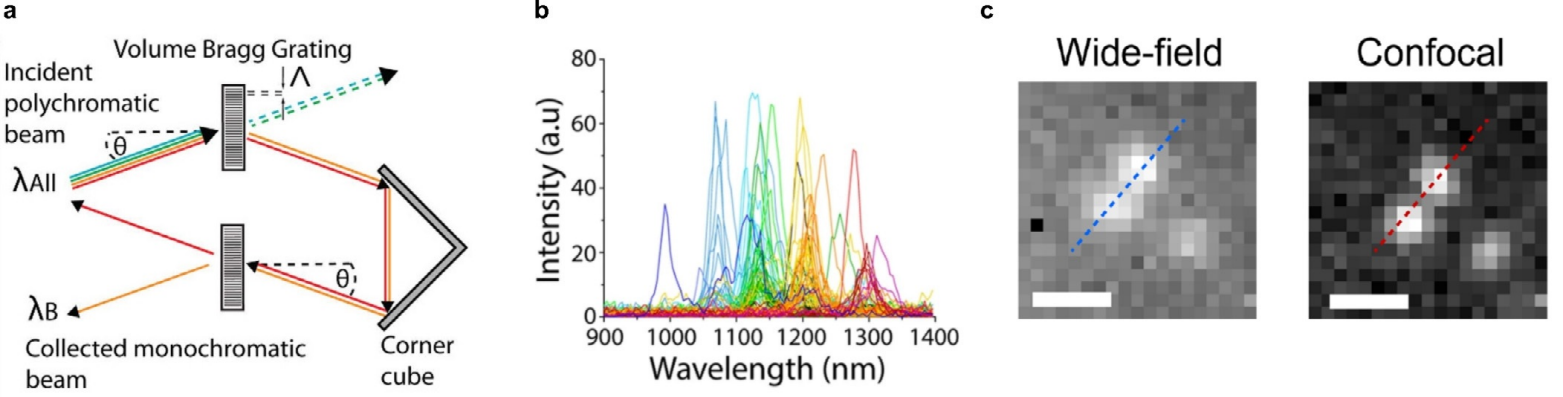
Advances in spatial and spectral resolution. a) A Volume Bragg grating filters one specific emission wavelength depending on the incident angle *θ*, refractive index *n*, and grating period *Λ*, which enables hyperspectral imaging. b) Detection of 12 different SWCNT chiralities without deconvolution in live human cervical cancer cells. Reprinted from Ref. [197] with permission. c) Improved NIR image contrast of NIR fluorescent beads with a spinning disc NIR fluorescence microscope in comparison to wide‐field microscopy. Reprinted from Ref. [198] with permission.

Hyperspectral microscopy (i. e. simultaneous imaging at different wavelengths) provides another approach to exploit the spectral variety for multiplexed SWCNT imaging. Roxbury et al. resolved up to 17 distinct chiralities with single nanotube spatial resolution.^[197]^ In contrast to organic fluorophores, more chiralities can be imaged simultaneously in a certain emission window due to the narrow emission bands of SWCNTs. The hyperspectral imaging is based on the use of a volume Bragg grating (VBG) placed between the emission port of an inverted fluorescence microscope and the NIR camera. The VBG filters one specific emission wavelength depending on optical properties such as the incident angle θ and the grating period Λ (Figure 23 a).

The spatial imaging of different chiralities is then achieved by measuring a continuous stack of 152 images within a time frame of 20 s and 10 min depending on the signal intensity and, thus, the integration time. By using this approach 12 different chiralities from DOC‐SWCNTs in live human cervical cancer cells could be detected (Figure 23 b). Additionally, individual SWCNTs adsorbed on a surface in live mammalian cells, murine tissues ex vivo, and zebrafish endothelium in vivo were imaged.

Another challenge of typical wide‐field microscopy setups is the poor *z*‐resolution. To improve this limitation, a NIR spinning‐disc confocal laser microscope with an increased resolution and imaging contrast was demonstrated by Zubkovs et al. (Figure 23 c).^[198]^ The custom‐built microscope was based on a spinning‐disc module integrated between a cooled InGaAs camera and the microscope body, which rejects out of focus light.

To achieve a maximized photon intensity, the lenses in the spinning disc unit were optimized for the NIR region. The authors showed in different biological applications the advantages of an improved lateral/axial resolution of 0.5±0.1 μm /0.6±0.1 μm (enhancement of 17 % /45 %) compared to the wide‐field configuration, reaching from single‐particle tracking over the spatial distribution of nanoparticles within an organelle to the optical in situ monitoring of glucose by GOx‐SWCNT‐based sensors embedded within an agarose gel. Overall, this approach showcases the opportunities for in vitro and in vivo imaging and sensing with improved spatiotemporal resolution.

## 4 Outlook and Perspectives

SWCNTs have shown their large potential as versatile building block for biosensors. The last few years have provided fundamental mechanistic insights, novel recations, and novel applications such as sensing in plants or primary cells. The advent of covalent quantum defect chemistry as well as the broader availability of monochiral samples will further advance typical figures of merit such as selectivity and robustness in demanding biochemical environments. One of the key challenges remains a basic understanding of molecular recognition and also signal transduction in the organic phase around these materials. Advances in this space can directly translate into superior selectivities. Additionally, high‐throughput or screening approaches will increase the speed of chemical discoveries. This is particularly interesting for applications in complex biological environments, where interactions become too complex to be predicted. Several milestones remain to be achieved before transfer into commercially available products. In vivo applications require long‐term evaluation of each sensor's toxicology and stability profile. Here, new covalent quantum defect functionalization strategies as well as the standardization of protocols and the purity of SWCNTs offer promising opportunities. Additionally, higher degrees of multiplexing and the adaptation of conventional cameras and readout systems will increase the application potential of SWCNT‐based sensors. In summary, SWCNT‐based biosensors offer a rich playground for chemistry and related disciplines and promise further advances and breakthroughs in the near future.

## Appendix

Table 1, 2, 3, 4, 5, 6, 7, 8, 9

**Table 1:** Detailed overview of fluorescent SWCNT‐based sensors of ROS/RNS.^[a]^

**Target**	**Biological system**	**Recognition strategy**	**Sensitivity**	**Selectivity**	**SWCNT chirality/ wavelength**	**Spatial** **resolution**	**Temporal resolution**	**Reversibility**
**Arsenite^[33]^**	In buffer (NaCl, MES, TES), embedded in plant tissue by syringe infiltration, uptake by plants through the roots	Screening approach: (GT)_5_‐SWCNTs FI increase TO‐PRO‐1‐(GT)_5_‐SWCNTs (dye) FI decrease	In buffer (NaCl) LOD 122 nm In hyperaccumulator plants theoretical LOD 4.7 nm/1.6 nm after 7/14 days	Over other heavy‐metal ions present in soil, e.g. Mn^2+^, Cd^2+^, Pb^2+^, Ni^2+^, Hg^2+^	HiPCO (9,4) at 1128 nm Dye for additional fluorescence in the visible range: 540 nm	Sub‐mm for a standoff distance of 1 m Subcellular with TO‐PRO‐1‐(GT)_5_‐SWCNTs (visible confocal microscopy)	Real time Intensity increase of sensors in plants 30 min after 10 μm arsenic introduction, after 5 h 11—15% increase	No
**Group 2 and 12 metal ions^[199]^**	In solution (NH_4_OH, NaOH)	SDBS‐SWCNTs FI decrease	0.5—5 mm tested	Varying quenching efficiency for different ions and SWCNT chiralities	HiPCO Analysis of each individual chirality at 925—1425 nm	ND	Measurement after 1 h of metal ion addition	ND
**Divalent ions^[25,109]^**	In Tris buffer, blood, black ink, tissue, within living mammalian cells	ssDNA‐SWCNTs ssDNA tested: (GT)_15_, (GC)_15_, 5’‐TAG CTA TGG AAT TCC TCG TAG GCA‐3’ Red‐shift	Concentration and chirality dependent red‐shift for different cations Hg^2+^ > Cu^2+^ > Co^2+^ > Ca^2+^ > Mg^2+[111]^ LOD 3.5 mm/8 mm in blood/tissue	Responds to different divalent ions	Chirality mix (6,5) peak mostly analyzed	ND	Within minutes	Yes, with dialysis to remove ions
**H_2_O_2_, H^+^, Fe(CN)_6_** ^**3− [200]**^	Embedded in collagen film	Collagen‐SWCNTs FI changes in discrete quenching steps	Single molecule	Different quenching equilibrium constants: 1.59/1.37 for H_2_O_2_/Fe(CN)_6_^3−^ at 20 μm, similar constant for H^+^ at 0.1 m	HiPCO (6,5), (10,2), (9,2), (8,7) 900—1600 nm	Single molecule 4 pixels, 900 nm × 900 nm^**^	Real time 1 s^**^	H_2_O_2_‐ and H^+^‐ induced quenching reversible with MnO_2_ catalyst for H_2_O_2_/NaOH decomposition
**H_2_O_2_** ^**[125]**^	H_2_O_2_ emitted from A431 human epidermal carcinoma cells	Collagen‐SWCNTs FI changes in discrete quenching steps	Single molecule	Over H^+^, NO, NO_2_^−^, NO_3_^−^, Dulbecco modified Eagle's medium (DMEM), L‐15, ^1^O_2_, O_2_ NO: largest forward rate constant, but binds nearly irreversibly, nitrites and nitrates: small rate constants	HiPCO	Sub‐μm	Real time Typical observation time 3000 s	Yes
**H_2_O_2_** ^**[126]**^	H_2_O_2_ generated from live human umbilical vein endothelial cells stimulated by vascular endothelial growth factor (VEGF) and artificial proangiogenic factor Eu(OH)_3_ nanorods	Collagen‐SWCNTs FI changes in discrete quenching steps	Single molecule 12.5—400 nm	see Ref. [125]	Chirality mix	Single molecule 300 nm pixel size^**^	Real time 1 s^**^	Yes
**H_2_O_2_** ^**[111]**^	Incubated in gemcitabine/ irinotecan‐treated Pancreatic ductal adenocarcinoma (PDAC) cells, implanted in tumors of PDAC murine model in response to gemcitabine	(GT)_15_‐SWCNTs FI quenching Alteration of Raman G‐band intensity	μmol L^−1^ range tested	ND	HiPCO Raman G‐band intensity at 1590 cm^−1^	μm	Real time Measurement after 0–48 h of gemcitabine‐/irinotecan‐treatment	Yes
**H_2_O_2_** ^**[31]**^	Embedded in leaves of Arabidopsis plants, in vivo monitoring of stresses (UV‐B light, high intensity light, pathogen‐related peptide)	Rational approach: Hemin‐binding‐aptamer SWCNTs Hemin binds Fe^3+^, which catalyzes a Fenton‐like reaction of H_2_O_2_ into hydroxyl radicals that quench fluorescence	10—100 μm	Over Ca^2+^, sugar (sucrose, glucose), plant hormone levels (methyl salicylate, abscisic acid (ABA), jasmonate (JA)), mechanical wounding	HiPCO >900 nm	Sub‐mm with standoff detection	Real time 60/120 min for saturation after pathogen‐/environmental‐related stress (UV‐B‐/high intensity light)	Yes
**H_2_O_2_** ^**[32]**^	Embedded in leaves of plants, in vivo monitoring of wound‐induced H_2_O_2_ waves and other stresses (high intensity light, heat stress, pathogen‐related peptide)	(GT)_15_‐SWCNTs FI decrease	μm—mm	Over other plant analytes (JA, auxin, ABA, salicylic acid, glutamates, Ca^2+^ ions), NO, NO_3_^−^, antioxidant O_2_, OH caused FI decrease, but not reversible	HiPCO 950 nm—1250 nm	Sub‐mm for a standoff distance of 1 m Wave speeds due to different stress treatments from 0.44 to 3.10 cm min^−1^	Real time Response within 4 min after wound infliction, recovery after 10–20 min	Yes, due to unbinding or consumption by antioxidants and peroxidases
**H_2_O_2_** ^**[112]**^	Embedded in wearable microfibrous textiles, in presence of peroxide‐producing macrophages, incorporated in wound bandages	(GT)_15_‐SWCNTs Chiralities show different quenching degree in response to H_2_O_2_	5 μm–5 mm	See Refs. [20, 32, 110]	HiPCO (8,7)/(9,4)	Sub‐μm	Real time within 5—30 min	Yes, within 50 min
**NO/H_2_O_2_** ^**[20]**^	Leaves mounted on microfluidic chamber, in vivo in leaves of Arabidopsis plants	(GT)_15_‐SWCNTs FI decrease	Tested: NO in vitro/in vivo 500 μm/50 mm, H_2_O_2_ in vitro/in vivo 10 mm/100 μm	See Ref. [110]	Monochiral (7,6) 1135 nm/1131 nm for NO/H_2_O_2_	Sub‐μm	Real time in vitro/in vivo: >600 s/50 s for saturation	Yes
**NO^[124]^**	In PBS, macrophage cells, mouse model	Rational approach: 3,4‐diaminophenyldextran‐SWCNTs FI decrease	LOD in solution/cells 70 nm/200 nm	Over other ROS/RNS (e.g. NO_2_^−^, NO_3_^−^, ONO_2_^−^, HNO, OCl^−^, OH*, H_2_O_2_)	Chirality mix 950—1350 nm	Sub‐μm in cells	Real time Bleaching rate *k*=0.856 s^−1^ for (10,5)‐SWCNTs	With reducing agent
**NO^[13]^**	Immobilized	Screening approach: (AT)_15_‐SWCNTs FI changes in discrete quenching steps	Single molecule LOD 300 nm	Over other ROS/RNS, dopamine, NADH, l‐ascorbic acid, riboflavin	HiPCO 900—1400 nm	Single molecule 4 pixels, 580 nm × 580 nm^**^	Real time Response time for NO: 1.1 s	Yes
**NO^[127]^**	Intravenous injection into mice, localization within the liver Implantation of alginate‐encapsulated SWCNTs within specific tissue	PEG‐(AAAT)_7_‐SWCNTs FI decrease	LOD 1 μm	Less quenching with other ROS/RNS	CoMoCAT‐SWCNTs (6,5) at 990 nm	Sub‐mm	For injection/implantation within s min^−1^ (93 % quenching after 30 min)	Yes
**NO^[128]^**	In cultures of A375 melanoma cells through micropinocytosis, NO production using NO‐releasing anticancer drug JS‐K VEGF‐mediated NO production in endothelial cells	(AT)_15_‐SWCNTs FI decrease	JS‐K concentrations of 16–28 μm tested VEGF concentrations between 10 ng mL^−1^ (LOD) and 100 ng mL^−1^	See Ref. [13]	HiPCO	Sub‐μm	Real time 400 s to reach steady state (after JS‐K addition)	Yes
**NO^[19]^**	In extracted chloroplasts and leaves of Arabidopsis plants, in vivo by infiltration through the leaf lamina	(AT)_15_‐SWCNTs FI decrease	40%—60% FI decrease	See Ref. [13]	HiPCO (8,6), (12,1), (11,3), (8,7), (10,5) 1150—1450 nm	Sub‐μm	Real time Less than 150 s for reaching steady state (after NO exposure)	ND
**O_2_** ^**[131]**^	In buffer (NaH_2_PO_4_/Na_2_HPO_4_)	Screening approach: 10 ssDNA sequences tested, e.g. (GT)_10_‐, (GT)_20_‐, (ATT)_4_‐SWCNTs FI decrease	Depending on ssDNA and SWCNT chirality, 9—40% quenching with 1 atm O_2_ compared to samples purged with 1 atm Ar	Relatively insensitive to pH	HiPCO	ND	Within seconds	yes
^**1**^ **O_2_** ^**[201]**^	SWCNTs challenged with different tyrosinase substrates for enzyme‐catalyzed reactions	Tyrosinase‐conjugated‐PEG_12_‐(GT)_15_‐amine‐SWCNTs Red‐shift and FI decrease	0.01–1 mm of tyrosinase substrates Response curves for different tyrosinase inhibitors measurable, IC_50_ values of suicide inactivation, e.g. 0.5 mm for kojic acid	For tyrosinase inhibitors which induce suicide inactivation of the enzyme	HiPCO 950—1350 nm	Single‐sensor	Within minutes 0.0281 nm min^−1^ with l‐tyrosine, 0.75 nm min^−1^ with pyrogallol	ND
**Single protons/pH^[47]^**	Embedded in agarose gel	SDBS‐SWCNTs FI changes in discrete steps	Single molecule	Response to chemical reactions with acid, base, diazonium reactants	HiPCO individual chiralities, e.g. (8,6) at 1175 nm, (8,3), (11,1)	Single molecule 4 pixels, 670 nm × 670 nm^**^	Real time 54 ms^**^	For acid reactions

[a] FI: fluorescence intensity, ND: not determined, **: limited by the Abbe limit, diffusion, and detection speed.

**Table 2:** Detailed overview of fluorescent SWCNT‐based sensors for neurotransmitters.^[a]^

**Target**	**Biological system**	**Recognition strategy**	**Sensitivity**	**Selectivity**	**SWCNT chirality/ wavelength**	**Spatial** **resolution**	**Temporal** **resolution**	**Reversibility**
**Dopamine (DA)^[15]^**	In PBS, immobilized	Screening approach: (GT)_15_‐SWCNTs and other candidates FI increase	Depending on chirality LOD in solution 11 nm	Response to other catecholamines with different magnitude	HiPCO (6,5) at 991 nm, (7,5) at 1044 nm, (10,2) at 1077 nm, (9,4) at 1132 nm, (8,6) at 1203 nm	Single sensor 4 pixels, 585 nm × 585 nm^**^	Real time Within seconds	Yes
**Reducing and oxidizing molecules, e.g. ascorbic acid/oxidized ascorbic acid, cysteine/cystine, DA, epinephrine (EPI), glutathione/oxidized glutathione, riboflavin, trolox^[63]^**	In PBS	FI increase with negatively charged polymer wrapping (e.g. ssDNA, poly(acrylic acid)) FI decrease with positively charged wrapping (polyallylamine‐SWCNTs) No FI change with PL‐PEG‐SWCNTs	100 μm tested Polymer‐wrapped SWCNTs that respond to reducing molecules (e.g. +141%, ascorbic acid) also respond to oxidizing molecules (e.g. 81%, riboflavin) Up to 250% FI increase with reducing molecules such as ascorbic acid, EPI, trolox with negatively charged wrapping, but redox potential alone cannot explain FI changes	Different responses for different small molecules	CoMoCAT (6,4), (8,3), (9,1), (6,5), (7,5) 800—1050 nm	ND	ND	ND
**Catecholamine (DA, EPI, norepinephrine (NE), amino acids, saccharides, riboflavin^[202]^**	In PBS	ssDNA‐SWCNTs (24 sequences tested, e.g. (ATT)_4_, (TAT_)4_, (ATTT)_3_) FI increase for catecholamines and ascorbic acid	Chirality‐dependent FI change, e.g. smaller diameter SWCNTs with (TAT)_4_ wrappings are less responsive than larger diameter SWCNTs	Different responses for different small molecules	HiPCO (8,3), (6,5), (7,5), (10,2), (9,4), (7,6), (12,1), (11,3)	ND	ND	ND
**DA^[100]^**	Collagen‐coated SWCNTs for increased cell adhesion, immobilized on glass, PC12 neuroprogenitor cells cultivated on top	Screening approach: (GA)_15_‐SWCNTs FI increase Interaction of hydroxy groups of DA with phosphate groups of DNA backbone pulls phosphate groups closer to the SWCNT surface, which increases the FI.	Single molecule LOD 100 pm	Not over catecholamine homologues (e.g. EPI, NE, l‐ascorbic acid), but PC12 cells mainly release dopamine	(6,5)‐enriched chiralities (6,5) at 980 nm	>20,000 sensors per cell 850 nm pixel size^**^	<100 ms	Yes
**Catecholamine (DA, EPI, NE)^[118]^**	In PBS, DA in presence of NE	Screening approach: different ssDNA‐SWCNTs, e.g. A_30_, (GT)_10_, (GC)_15_, (AT)_15_, (GT)_10_, for different selectivity and sensitivity FI increase (GT)_10_‐SWCNTs for sensing DA in presence of NE	LOD mostly in single‐digit nm regime LOD of dopamine in presence of NE 4.9 nm, without NE 0.1 nm	Dissociation constants vary between 2.3 nm ((GC)_15_‐SWCNTs + NE) and 9.4 μm ((AT)_15_‐SWCNTs + DA)	(6,5)‐enriched at 987 nm	Single nanosensor	Real time Within seconds	Yes
**Catecholamine release, e.g. DA^[120]^**	Brain slices from dorsal striatum of mouse incubated in artificial cerebrospinal fluid (ACSF)	(GT)_6_‐SWCNT FI increase	10 nm—100 μm	Over γ‐aminobutyric acid, glutamate, acetylcholine In the presence of pharmacological DA receptor ligands Not for DA over NE	HiPCO (9,4) at 1128 nm	μm	Sub‐second	Yes
**DA^[121]^**	In PBS	(GT)_10_‐SWCNTs FI increase	>20% intensity increase (100 nm)	ND	Monochiral (6,5) at 980 nm	ND	ND	ND
**DA, riboflavin, H_2_O_2_, pH^[176]^**	In PBS	ssDNA‐SWCNTs, e.g. (GT)_5_, (GT)_15_, A_30_, T_30_, C_30_	In most cases FI change does not correlate with the number of adsorbed ssDNA molecules	Different responses for different small molecules	CoMoCAT	ND	ND	ND
**DA^[203]^**	Acute mouse brain slices in ACSF, electrically evoked neurotransmitter release	PEG_2000_‐PL‐(GT)_6_‐SWCNTs FI increase Improved sensor response due to PL‐induced quenching of SWCNT baseline FI	In vitro: increased sensor response to 200 μm DA of Δ*I*/*I*_0_=2.01 compared to nonpassivated sensors Δ*I*/*I*_0_=1.44 Ex vivo in brain slices: increased responsivity by 52±8 % and improved number of identified ROI by 160±50 %	Reduced protein adsorption of 28% compared to nonpassivated sensors	HiPCO	Sub‐μm	Real time 111 ms^**^	Yes
**DA^[80]^**	Self‐assembled on neutravidin‐coated microscopy slides	Screening approach: Combination of covalent and noncovalent SWCNT functionalization (GT)_15_‐amine‐PEG2k‐biotin‐SWCNTs (GT)_15_ is able to detect DA, while biotin is able to detect neutravidin and streptavidin	Δ*I*/*I*_0_=0.8658±0.1986 for 25 μm DA	ND	HiPCO (10,2) at 1053 nm	ND	Real time Sub‐seconds	Yes
**DA, H_2_O_2_** ^**[139]**^	SWCNT uptake, transport, and programmed release by human immune cells (neutrophilic granulocytes, neutrophils)	DA: (GT)_15_‐SWCNTs, FI increase H_2_O_2_: Heminaptamer‐SWCNTs, FI decrease	100 nm DA and 100 μm H_2_O_2_ tested Sensors functional after cargo transport and release	ND	CoMoCAT	ND	Within seconds	ND
**DA^[136]^**	Immobilized on surface in complex medium (e.g. Schneider's medium) In presence of CaCl_2_, Drosophila neurons	Locked nucleic acid bases incorporated into (GT)_15_ sequence	LOD 10 nm Superior compared to (GT)_15_‐SWCNTs in presence of CaCl_2_/Schneider's medium	Over Ca^2+^	HiPCO (7,5), (7,6)	ND	Measurement after 5 min—24 h	ND
**DA, NE^[119]^**	In PBS, NaCl, protein‐rich media (DMEM + 10 % fetal bovine serum (FBS)) and ACSF	Screening approach of (GT)_x_ sequences: (GT)_6_‐SWCNTs FI increase	Down to 100 nm tested 3500%/2300% increase to 100 μm NE/DA in PBS 143%/260% increase to 100 μm DA in DMEM + FBS/ ACSF	Over acetylcholine, serotonin, histamine, γ‐aminobutyric acid, glutamate, glycine, aspartate, DA transporter inhibitor nomifensine, DA receptor agonist quinpirole, antagonist sulpiride, and haloperidol	HiPCO (9,4) at 1127 nm	ND	Measurement after 5 min/1 h of analyte incubation	ND
**DA, riboflavin, pH, H_2_O_2_** ^**[49]**^	Immobilized	DA: (GT)_40_‐SWCNTs, Riboflavin: (GT)_40_‐SWCNTs, pH: PEG‐PL‐SWCNTs, H_2_O_2_: hemin‐binding aptamer‐SWCNTs	Higher brightness of monochiral sensors, μm range for H_2_O_2_ detection	Affected by surfactant residues, differences in sensing magnitudes for different sequences/analytes	Monochiral (6,5) at 990 nm, (7,6) at 1130 nm	ND	ND	ND
**DA^[135]^**	Release from primary neurons (varicosities) using a sensor paint (AndromeDA)	(GT)_10_‐SWCNTs FI increase Sensors are applied on top of cells as sensor coating/paint (AnromeDA)	1 nm–100 μm EC_50_: 299 nm	Over neurons treated with inhibitor reserpine (depletes DA)	CoMoCat	Subcellular Discriminates individual varicosities (up to 100 within a single imaging field)	Real time 15 images s^−1^	Yes
**Serotonin^[27]^**	Release from human blood platelets	Serotonin‐binding‐aptamer‐SWCNTs Fl increase	100 nm—10 μm	Over tryptophan, histamine, tyrosine, glucose 11 % increase to 100 nm DA compared to 45 % increase to serotonin	(6,5)‐enriched chiralities (6,5) at 995 nm	Subcellular 63 nm pixel size^**^	Real time (Sub)second	Yes
**Serotonin^[134]^**	Acute striatal brain slices of mice and exogenous addition of serotonin	Screening approach: Identification of polynucleotides by exponential enrichment for selective ssDNA‐SWCNTs	100 nm—50 μm	Over serotonin analogues, metabolites, and receptor‐targeting drugs (e.g. 5‐hydroxytryptophan, 5‐methoxytryptophan, fluoxetine) FI increase due to DA of 1400 %	HiPCO (8,6) at 1195 nm	Sub‐μm	Real time (Sub)second	Yes

[a] FI: fluorescence intensity, ND: not determined, **: limited by the Abbe limit, diffusion and detection speed.

**Table 3:** Detailed overview of fluorescent SWCNT‐based sensors for other small molecules.^[a]^

**Target**	**Biological system**	**Recognition strategy**	**Sensitivity**	**Selectivity**	**SWCNT chirality/ wavelength**	**Spatial resolution**	**Temporal resolution**	**Reversibil‐ity**
**Acetic acid^[145]^**	In DI water, wine Analytes injected into glass bottle with inlet and outlet tubes	Screening approach: Peptide‐encapsulated SWCNTs (YK‐SWCNT) detect volatile molecules Synthesized peptide (YK): contains positive charge to potentially improve acetic acid binding to the sensor and aromatic amino acids to enhance π‐π interaction and binding to SWCNTs FI decrease	In DI water: concentrations of 0.05 %–3.2 % (v/v) tested In wine: 0.05 % and 0.1 % (v/v) tested	Slightly increased sensor response at higher humidity results in stronger quenching due to additional binding of water molecules to the sensor In wine, other volatile compounds interfere with sensor	CoMoCAT (6,5) at 970–1050 nm	ND	Real time Steady state after 40 s In wine: no steady state after 90 s, but significant FI change	Yes, but slow. Illumination with UV light enhances recovery speed
**Adenosine‐5’‐triphosphate (ATP)^[140]^**	In Tris‐HCl buffer, cellular ATP detection in living HeLa cells	Rational approach: PL‐PEG‐functionalized SWCNT/luciferase enzyme conjugate FI quenched by the luciferase‐mediated bioluminescence reaction product oxyluciferin in the presence of ATP and d‐luciferin	LOD 240 nm	Over Adenosine 5’‐monophosphate, adenosine 5’‐diphosphate, cytidine 5’‐triphosphate, guanosine 5’‐triphosphate	Chirality mix	μm	Real time Measurement after 15 min of analyte addition	No
**DA, riboflavin, pH, H_2_O_2_** ^**[49]**^	Immobilized	DA: (GT)_40_‐SWCNTs Riboflavin: (GT)_40_‐SWCNTs pH: PEG‐PL‐SWCNTs H_2_O_2_: hemin‐binding aptamer‐SWCNTs	Higher brightness of monochiral sensors, μm range for H_2_O_2_ detection	Affected by surfactant residues, differences in sensing magnitudes for different sequences/analytes	Monochiral (6,5) at 990 nm, (7,6) at 1130 nm	ND	ND	ND
**Doxorubicin^[143]^**	In water or water/DMSO mixture, human blood plasma, mouse tissue	Screening approach: CCCCCCCCAGAATTACTTCCCCCCCC‐SWCNTs FI change and red‐shift (shift for readout)	LOD 8 μm	Over dacarbazine (chemotherapeutic, commonly co‐injected with doxorubicin) Not over other anthracycline chemotherapeutic drugs (epirubicin, daunorubicin)	Chirality mix (7,6), (6,5)	ND	Steady state within minutes 20 min for diffusion of doxorubicin into tissue	Yes
**Doxorubicin^[115]^**	In saline‐sodium citrate buffer, FBS, intracellularly (incubated in Raw 264.7 murine macrophage cells), incorporated into membrane and implanted into peritoneal cavity of living mice	(GT)_15_‐SWCNTs FI decrease and red‐shift	In buffer: 500 nm–50 μm In serum: LOD 5 μm In vivo: LOD 50 μm	Not over other DNA‐intercalating agents (SYBR green, Hoechst 33258, ethidium bromide, 1‐pyrenebutyric acid)	HiPCO (9,4)	Sub‐μm	Real time Within minutes	No
**Human steroid hormones (cortisol, progesterone)^[144]^**	In DMSO, PBS, 10% mouse serum, subcutaneously implanted in mice (progesterone detection, SWCNTs in HG)	Semirational approach: Cortisol sensor: p(AA_197_‐ran‐AC_5_)‐SWCNTS Progesterone sensor: p(AA_53_‐ran‐S_22_‐ran‐AC_4_)‐SWCNT FI increase	In solution: cortisol: 10–100 μm, progesterone: 5–100 μm In serum: 12 % fluorescence increase at 100 μm progesterone In HGs inside dialysis bag: three times higher sensor response (22.1 %) compared to control	Over other steroids by a factor of 2 Progesterone additionally over other small molecules and large proteins	HiPCO (6,5) for cortisol sensor, (7,6) for progesterone sensor	Sub‐mm	3 h needed for stabilization of fluorescence signal in HG	Yes
**Nitroaromatics^[141]^**	In solution, immobilized on glass in Tris buffer	Screening approach: Bombolitin II‐SWNTs Wavelength shift (AT)_15_‐SWCNTs for TNT detection FI changes in discrete quenching steps	Single molecule	Distinction with PCA possible: over several containing nitroaromatic and non‐aromatic compounds containing nitro groups, e.g. picric acid, cyclotrimethylenetrinitramine, 2,4‐dinitrophenol, over other nitroaromatics (e.g. RDX, TFM) but 2,4‐dinitrotoluene and 2‐nitrophenol show quenching to a lesser degree	HiPCO Analyzation of 8 chiralities, e.g. (7,5), (11,3)	Single molecular	Real time 500 ms–1 s	Yes
**Nitroaromatics/picric acid^[30]^**	Within spinach plant leaf mesophyll by root uptake or through direct uptake through leaf surface	Bombolitin II‐SWCNTs FI quenching	85 %/78 % quenching with 400 μm picric acid by root/leaf uptake	ND	HiPCO (6,5)‐enriched chiralities	0.5 mm pixel size for a stand‐off distance of 0.85 m	Real time 5–15 min transport time of picric acid uptake from the roots to the leaves, 40–50 min for saturation Sensor response after 10 s by leaf uptake	No
**Odors, volatile molecules^[146]^** **Alcoholic vapors (ethanol, methanol, propanol, 2‐propanol)** **Distinction between aromas of red wine, beer, vodka** **Distinction between limonene, undecanal, and geraniol vapors**	Analytes injected into glass bottle with inlet and outlet tubes	Array of five different peptide‐encapsulated SWCNTs adsorbed on a nitrocellulose paper, recognition by LDA and machine learning FI increase	LOD around 216 ppm of ethanol	Slightly increased sensor response at higher humidity Discrimination of a mixture of ethanol and methanol	CoMoCAT (6,5) at 1000 nm	ND	Real time Response within seconds Steady state after 24 s, some continue to rise after 45 s	Yes, within 160 s
**Phosphodiesterase type 5 (PDE5) inhibitor Vardenafil^[147]^**	In PBS	Screening approach: Poly(methacrylic acid‐*co*‐styrene)‐SWCNTs with methacrylic acid and styrene at 90:10 ratio Synthetic corona mimics H loop of native enzyme PDE5 FI decrease	LOD 0.02–0.2 μm by varying the polymer length Sensor has smaller binding affinity than the enzyme, interaction is disrupted in the presence of PDE5a	Over 22 tested small molecules, not over inhibitor Sildenafil (chemical similar), but smaller emission modulation	HiPCO (8,3), (6,5)	ND	5 min incubation time	ND
**Polyphenols (tannins, flavonoids, …)^[148]^**	In plant extracts, tissue culture media Sensors embedded in agar, pathogen‐induced release from soybean roots (stimulated with pathogen‐derived elicitor or mechanical wounding)	PEG‐PL‐SWCNTs FI decrease, red‐shift	*K*_d_=90 nm, saturation in lower μm range (−80 % FI change, 20 nm wavelength shift) Differences for different polyphenols	Over potential interferant molecules from the root such as sugar, H_2_O_2_	CoMoCAT, HiPCO, and monochiral (6,5)	Sub‐mm with standoff detection	Real time (s)	ND
**Porphyrins/ heme^[204]^**	Detection of plasma samples with solvent extraction method	Heme‐binding‐aptamer‐SWCNTs FI decrease	LOD 20 nm	Over other porphyrins, BSA, lysozyme, phthalocyanine	CoMoCAT (7,5)	ND	Real time Immediately	ND
**Riboflavin, l‐thyroxine, oestradiol^[70]^**	In buffer Riboflavin imaging in live Raw 264.7 macrophage cells	Screening approach: Oestradiol: Rhodamine isothiocyanate‐difunctionalized poly(ethylene glycol)‐SWCNTs FI decrease l‐thyroxine: Fmoc‐l‐phenylalanine PEG‐SWCNTs FI decrease Aliphatic chain and Fmoc group adsorb onto SWCNT, strong interaction between Fmoc and l‐thyroxine leads to molecular recognition Riboflavin: 53 mol/mol boronic‐acid‐substituted phenoxydextran‐wrapped‐SWCNTs (BA‐PhO‐Dex‐SWCNTs) Red‐shift through polymer dielectric change	0.6 FI change to 100 μm oestradiol/l‐thyroxine and 11 nm shift to riboflavin	Over 35 biological molecules	Chirality mix Deconvoluted into 8 chiralities (7,6) at 1147 nm for tracking of riboflavin in macrophage cells	Sub‐μm	In macrophage cells within seconds	Yes, for riboflavin through riboflavin‐binding protein
**Riboflavin^[113]^**	SWCNTs embedded in HG with water and barium chloride, subcutaneously implanted into mice	(GT)_15_‐SWCNTs FI decrease	10 mm riboflavin results in 48 % quenching for HG SWCNTs with a concentration of 10 mg L^−1^ LOD in 5.4 mm depth tissue	Pore size of HG can be engineered to exclude large molecular weight interfering molecules, average pore size: 3.2 nm	(6,5)‐enriched SWCNTs (6,5)	Sub‐mm	Two characteristic quenching times: 14.1 min (riboflavin‐SWCNT reaction), 5.8 h (riboflavin diffusion) for HGs with 10 mg L^−1^ SWCNT concentration	ND
**Riboflavin^[142]^**	In HG for in vitro and ex vivo tissue measurement of marine organisms (Sparus aurata, Stenotomus chrysops, Galeus melastormus)	(AC)_15_‐SWCNTs FI decrease	In vitro: 1–100 μm in vivo: LOD in 7 mm skin and muscle tissue	Pore size of HG can be engineered to exclude large molecular weight interfering molecules, average pore size: 15 nm	CoMoCAT	mm	In vitro: immediate response, fluorescence decreases continuously while riboflavin diffuses to sensors	ND

[a] FI: fluorescence intensity, ND: not determined, HG: hydrogel.

**Table 4:** Detailed overview of fluorescent SWCNT based sensors for lipids.^[a]^

**Target**	**Biological system**	**Recognition strategy**	**Sensitivity**	**Selectivity**	**SWCNT chirality/ wavelength**	**Spatial resolution**	**Temporal resolution**	**Reversibility**
**Endolysosomal lipids^[117]^**	In PBS, in fibroblasts from an NPC patient (Niemann‐Pick type C, lysosomal storage disease)	Screening approach: (GT)_6_‐SWCNTs Wavelength shift	For lipid analogues, e.g. PEG‐cholesterol: 10 nm—1 μm	Over BSA, double‐stranded DNA from salmon, carboxymethyl cellulose (CMC)	HiPCO (8,6) at 1200 nm	Subcellular	< 2 min for incubation of fibroblasts from NPC patient with SWCNTs Measurement after 24 h	Yes, upon administration of a drug (hydroxypropyl‐β‐cyclodextrin) that reverses disease phenotype
**Endolysosomal lipids^[149]^**	In cell culture medium with 10 % FBS, intravenous injected into mice with NPA/B and NPC disease to detect lipid accumulation in the Kupffer cell endolysosomal organelles	Screening approach: CTTC_3_TTC‐SWCNTs Blue‐shift	In cell culture medium 6–8 nm blue shift In vivo 4.2–5.3 nm blue shift	Minimal response to BSA, genomic DNA, CMC Not over different lipid types	Chirality mix (9,4) at 1125 nm	μm	Within min–h	ND

[a] FI: fluorescence intensity, ND: not determined.

**Table 5:** Detailed overview of fluorescent SWCNT based sensors for proteins.^[a]^

**Target**	**Biological system**	**Recognition strategy**	**Sensitivity**	**Selectivity**	**SWCNT chirality/ wavelength**	**Spatial resolution**	**Temporal** **resolution**	**Reversibility**
**β‐Carotene^[116]^**	In canola oil	Screening approach: (GT)_15_‐SWCNTs adsorbed onto nitrocellulose paper	Dissociation constant 2.2 μm	Less FI increase to fat‐soluble vitamins (menadione, retinyl acetate and α‐tocopherol)	CoMoCAT	ND	Real time	ND
**Aggregation status of human IgG^[159]^**	In HG	Chitosan‐SWCNTs modified with Cu^2+^‐NTA/His‐tagged protein A interacting with human IgG leading to ion based proximity quenching	LOD below 660 nm	ND	CoMoCAT (6,5)	mm	Real time Within 5 min	Usable twice
**Albumin^[152]^**	In clinical urine samples of patients with microalbuminuria Samples need to be filtered to remove small interfering molecules	Screening approach: Constructed polymer mimics fatty binding to albumin Carboxylate‐functionalized polycarbodiimide polymer‐SWCNTs incorporated into acrylic‐based paint Blue‐shift, FI increase	LOD 3 mg L^−1^	Potential interfering proteins such as transferrin, γ‐globulins, degraded albumin showed either negligible change or red‐shift	HiPCO (9,4)	ND	Saturation after 20 min FI change: 0.214 min^−1^ Wavelength change: 0.1845 min^−1^	ND
**Avidin**	In solution	SWCNTs noncovalently bound to dye‐ligand conjugates (biotinylated anthracene), which are covalently bound to a biological receptor ligand (biotin), which binds to the analyte FI increase	LOD 1–5 nm	Response to BSA, but with three orders less sensitivity	HiPCO 900—1500 nm	ND	ND	Yes
**Cardiac** **biomarker troponin T^[64]^**	In HG	Rational approach: Chitosan‐wrapped SWCNTs noncovalently modified with troponin antibody using a Ni^2+^‐NTA/hexahistidine‐tagged protein mechanism for divalent ion‐based proximity quenching FI increase	LOD 2.5 nm	Over BSA, IgG, cancer biomarker Pisum sativum agglutinin (PSA), 10000 times diluted human plasma (7 μg mL^−1^ proteins) Affected by viscosity change (e.g. 100 times diluted plasma), unable to quantitatively detect in full plasma	Chirality mix 1000–1400 nm	ND	Real time Within 5 min saturation	Yes
**Cell surface receptors** **CD20, HER2/neu^[205]^**	Incubation of cells in SWCNT solution	Rational approach: PL‐PEG‐NH_2_‐SWCNTs conjugated to antibodies Rituxan antibody to recognize CD20 cell surface receptor on B‐cell lymphoma Herceptin to recognize HER2/neu positive breast cancer cells	ND	Rituxan‐SWCNTs: over T‐cell lymphoma Herceptin‐SWCNTs: over HER2/neu negative cell line	HiPCO 900–2200 nm	Sub‐μm	1 h incubation time before measurement	ND
**Electrostatic charge accumulation mediated by cell membrane proteins^[206]^**	SWCNTs in contact with cell monolayer (HeLa cells, murine fibroblast cell line (NIH/3T3), human lymphocyte cell line (Jurkat))	(AT)_15_‐SWCNTs Wavelength shift Determined between membrane‐bound SWCNTs and SWCNTs in serum‐free solution	Emission energy correlated with the degree to which a cell adheres to a substrate and the zeta potential of the cell Jurkat cells: Δλ=6.02±0.28 nm NIH/3T3: Δλ=4.97±0.23 nm HeLa cell: Δλ=2.72±0.12 nm	Cell type dependent trend	HiPCO 900–1600 nm	20 μm pixel size^**^	ND	ND
**Fibrinogen^[71]^**	In serum (10% FBS in PBS)	Screening approach: Dipalmitoyl‐phosphatidylethanolamine (DPPE)‐PEG (5 kDa)‐SWCNT	Lowest concentration tested 0.05 mg ml^−1^	Negligible response of < 5 % intensity to 13 proteins (some are highly abundant in blood and others are relatively rare but of clinical significance)	HiPCO (9,4), (7,6)	Single sensor	Real time Signal quenching occurs rapidly (within seconds), Saturation within minutes	ND
**Glutathione‐S‐transferase (GST) fusion proteins^[154]^**	In solution	Glutathione‐(TAT)_6_‐SWCNTs Modulation of FI and wavelength in response to GST and GST fusions depending on SWCNT structure	LOD 10 nm	Responds to multiple classes of GST‐tagged proteins (e.g. cell‐cycle proteins, RNA‐binding proteins, ovarian cancer protein biomarkers) Affected by powdered milk (protein blocking agent), BSA	HiPCO (8,6), (9,1)	ND	Real time 5 min incubation time	ND
**Green fluorescent protein (GFP), GFP‐fusion proteins^[79]^**	Incubation with GFP	GFP‐binding nanobody‐SWCNTs, covalently bound aryl maleimide Quantum defects introduced by light‐driven diazonium chemistry serve as anchor points for peptides and proteins, conjugation of cysteine‐containing proteins to maleimide	ND	Over negative control, where no diazonium salt was added	E_11_^*^ at 1135 nm	μm	ND	ND
**Gynecologic cancer biomarker** **human epididymis protein 4, cancer antigen 125, chitinase‐3‐like protein, mesothelin^[160]^**	In 10% FBS, uterine lavage samples	Machine‐perception‐based sensor array consisting of 132 distinct DNA‐SWCNT complexes: 11 ssDNA‐SWCNT‐based sensors with 12 chiralities, e.g. (AT)_11_‐, (GT)_12_‐, (ATT)_4_, (TCT)_5_‐SWCNTs FI increase/decrease and wavelength shift	LOD in pm range	Simultaneous distinction/ detection of biomarkers with classification accuracy (F1‐score) of ca. 0.95	HiPCO Deconvoluted to extract individual chiralities, e.g. (6,5), (8,4), (10,3), (7,5), (7,6), (8,3)	ND	ND	ND
**Human α‐thrombin^[207]^**	In PBS	Rational approach: Dye (FAM) labelled human α‐thrombin binding aptamer‐SWCNTs FI increase	LOD 1.8 nm Dynamic range 4–150 nm	In presence of 100 nm of other proteins (BSA, HAS, IgG)	HiPCO	ND	Incubation of target 2–3 h	ND
**Immunoglobulin IgG^[104]^**	In HG	Rational approach: Chitosan‐wrapped SWCNT noncovalently modified with immunoglobulin‐binding proteins A using a Cu^2+^‐NTA/His‐tagged protein mechanism for divalent ion based proximity quenching FI increase	LOD 10 ng mL^−1^	ND	HiPCO	ND	Real time Within seconds Equilibrium reached within 5 min	ND
**Immunoglobulin (human IgG, mouse IgM, rat IgG2a, human IgD)^[151]^**	Ink printed	Rational approach: Chitosan‐SWCNTs noncovalently modified with immunoglobulin‐binding proteins. Cu‐NTA chelating chemistry with histidine tag for divalent ion based proximity quenching Binding proteins: protein A, protein G, protein L FI increase	LOD 25 μg mL^−1^ of human IgG, 2.5 μg mL^−1^ at 20x magnification	Different association constants for different antibody/binding proteins	CoMoCAT (6,5) at 980 nm	Sub‐mm	Real time 1 s^**^ Response within seconds	Yes
**Insulin^[155]^**	In buffer	Insulin‐binding aptamer‐SWCNTs FI quenching	Lowest concentration tested: 9 nm	Over BSA, proteinase K	HiPCO (6,5)	Sub‐μm	ND	ND
**Insulin^[156]^**	In collagen extracellular matrix Insulin secreted by pancreatic β‐ cells due to glucose addition	Insulin‐binding aptamer‐SWCNTs FI quenching upon analyte binding by photoinduced charge‐transfer mechanism (electron transfer from conduction band of SWCNTs to LUMO of the bound insulin)	LOD 10 nm	Over BSA, proteinase K, cell culture media RPMI 1640, glucose	CoMoCAT (7,5) at 1044 nm	Sub‐μm	Real time Within seconds FI quenching rate 5.85×10^14^ m ^−1^s^−1^ diffusion reaction rate 0.129 s^−1^	Yes, through enzymatic proteolysis within 1 h enzyme: α‐chymotrypsin
**Insulin^[101]^**	In PBS, blood serum (10 % FBS in PBS)	Screening approach of PEG‐conjugated lipids C_16_‐PEG(2000 Da)‐ceramide‐SWCNTs FI decrease	180 pm—3.5 μm tested In FBS reduced response	Over library of proteins (< 5 % intensity change) Not over apolipoproein A‐I and longer fragments of isolated α‐ and β‐peptide chains of insulin	HiPCO (10,2)	ND	Measurement after 30 min of insulin addition	ND
**Insulin^[158]^**	In PBS, Krebs‐Ringer HEPES buffer (KRHB), secreted by pancreatic β‐cells due to glucose addition	C_16_‐PEG(2000 Da)‐ceramide‐SWCNTs (AT)_15_‐Insulin‐binding aptamer‐SWCNT FI decrease	8.1 nm–74 mm tested C_16_‐PEG‐ceramide‐SWCNTs: response stable and reproducible (AT)_15_ ‐Insulin aptamer‐SWCNT: diminishing response over time, batch‐to‐batch variations	Over BSA, KRHB, IBMX, glucose	HiPCO (10,2) (6,5)	ND	5 min incubation time Detection in real time	ND
**Integrins^[66]^** **Human platelet integrin α_IIβ_β_3_**	Immobilized on epithelial cells	Arg‐ly‐Asp (RGD) peptide conjugated to different ssDNA sequences in linear ssDNA‐RGD or bridged ssDNA‐RGD‐ssDNA geometries Integrin affinity of RGD motif depends on its conformational freedom due to ssDNA‐RGD geometry	IC_50_ values varied from 309 nm for C_20_‐RGD‐SWCNTs to 29 nm for (GT)_15_‐SWCNTs	DNA sequence affects overall RGD affinity	CoMoCat	ND	ND	ND
**Green fluorescent protein (GFP), GFP‐fusion proteins^[67]^**	Tracking of motor protein (Kinesin‐5‐GFP) in embryos of Drosophila melanogaster	Rational approach: GFP binding nanobodies conjugated to DNA‐wrapped SWCNT GBP‐(GT)_20_‐SWCNTs	ND		CoMoCAT (6,5)	Single sensor	Real time Resolving the velocity of a molecular motor in vivo at 1340 nm s^−1^	ND
**Ovarian cancer biomarker HE4 (human epididymis protein 4)^[65]^**	In serum (10 % FBS), living mice, sensors loaded into semipermeable PVDF membrane capillary	Rational approach: HE4 Antibody (Ab) is goat polyclonal anti‐HE4 IgG antibody Ab‐(TAT)_6_‐amine‐SWCNT passivated with BSA	In serum: 10 nm–500 nm	Mostly over nontarget proteins like uPA, CA‐125 (another ovarian biomarker), BSA or 93 % FBS	HiPCO 900 — 1400 nm	Single sensor	Change after 1 min, stabilized signal after 1 h	ND
**Prostate cancer biomarker uPA^[106]^**	In PBS, blood, FBS, plasma	Rational approach: Mouse monoclonal IgG anti‐uPA‐(TAT)_6_‐SWCNTs passivated with BSA to increase sensitivity Red‐shift	LOD In buffer 100 pm In serum 25 nm In plasma 100 nm	Increased selectivity in complex protein environment due to BSA passivation of SWCNTs	HiPCO (9,4), (8,6), (8,7)	ND	Saturation after 30/90 min in human serum/ plasma	ND
**Prostate tumors^[21]^**	In tissues/ tissue‐like phantoms, serum (FBS), incubated on human prostate cancer cell lines, in vivo in mice (intravenous injection)	Rational approach: Prostate specific membrane antigen (PSMA) antibody conjugated to M13‐phage SWCNTs	At 2 μg ml^−1^ SWCNTs detection depth up to 2.5 cm in tissue‐like phantoms	4‐fold improved uptake in PSMA positive prostate tumors compared to control, stable in serum	HiPCO	mm in vivo	After 2–4 h post injection	ND
**RAP1^[105]^**	Release from *E. coli* cell lysates	Rational and screening approach: RAP1‐aptamer‐(AT)_11_‐SWCNT With spacers between (AT)_11_ enhanced FI increase (4x) due to less adhesion of aptamer to SWCNT surface	Zeptomolar protein detection	Over eight tested other proteins	Chirality mix (6,5)	Single cell	ms^**^	Yes
**SARS‐CoV‐2 spike protein^[157]^**	In saliva, viral transport medium	Rational approach: ACE2‐(GT)_6_‐SWCNTs, passivation with PE‐PEG FI increase	LOD 12.6 nm/ 10^4^–10^6^ viral copies per μL in solution/on surface	Less increase with other viral spike‐like proteins (SARS‐CoV‐1 S RBD, MERS S RBD, FLU hemagglutinin subunit, serum albumin), in biological fluids (e.g. saliva, viral transport medium)	HiPCO 1130 nm	ND	Within 90 min/5 s in solution/ immobilized	ND
**Wheat germ agglutinin (WGA) (sugar‐binding lectin protein)^[153]^** **Sugars/*N*‐acetylglutamic acid (GlcNAc) and** ***N*** **‐acetylneuraminic acid (Neu5Ac)**	In buffer, complex biological media, immobilized	Screening approach: Peptoid‐SWCNT FI decrease in response to WGA Sensor shows preserved protein activity and is able to bind its target sugars	LOD 3.4 μm	Minor responses to protein A, BSA, NeutrAvidin, lysozyme FI increase to peanut agglutinin, ConA Attenuated ability to detect WGA in complex biological media (DMEM, FBS) Over other sugars commonly bound by lectins (fructose, galactose, glucose, mannitol mannose, sucrose), but not fucose	HiPCO (7,6)	Single sensor	Equilibrium reached within 1 h	ND

[a] FI: fluorescence intensity, ND: not determined, HG: hydrogel.

**Table 6:** Detailed overview of fluorescent SWCNT based sensors for sugar.^[a]^

**Target**	**Biological system**	**Recognition strategy**	**Sensitivity**	**Selectivity**	**SWCNT chirality/ wavelength**	**Spatial resolution**	**Temporal resolution**	**Reversibility**
**d‐glucose^[24,208]^**	SWCNT solution loaded into dialysis capillary imaged through human epidermal tissue sample	Rational approach: GOX‐SWCNTs with Fe(CN)_6_^3−^ addition as electroactive species results in FI decrease or Fermi level shift into the VB due to irreversible adsorption on the SWCNT surface Addition of analyte results in increasing restored FI GOX catalyzes the reaction of d‐glucose to d‐glucono‐1,5‐lactone with a H_2_O_2_ co‐product	LOD 34.7 μm	ND	HiPCO	μm	Within 80 s	ND
**d‐glucose^[108]^**	In PBS, immobilized	Rational approach: Glucose‐binding protein (GBP) covalently conjugated to PVA‐SWCNTs Quenching in discrete steps in response to glucose due to conformation change of GBP	2.5–50 mm Linear until 10 mm	Over fructose, mannose	CoMoCAT (8,3), (6,5), (7,5)	Single‐sensor 500 nm pixel size^**^	Immediately (s)	Yes
**Glucose^[11]^**	In BSA + PBS BSA to prevent concanavalin A (ConA, lectin with four glucose‐binding sites) adsorption to the walls of the cuvette	Rational approach: Phenoxydextran‐SWCNTs with ConA Competitive binding between target analyte and dextran, ConA introduces protein‐controlled aggregation and FI decrease, introduction of glucose causes dissolution of aggregate and FI recovery	LOD 3.8 nm Response range based on ConA concentration	Over glycerol (hydrogen‐bond‐breaking agent)	Chirality mix	ND	3–28 min to reach steady state	Yes
**Glucose^[162]^**	In solution, 36°C	Rational approach: GOX‐DNA‐SWCNTs with 2 mm/5 mm potassium ferricyanide (PFC) as electron transfer mediator FI increase	LOD 1 mm With increasing amount of glucose portions (1–2 mm) less sensitive (FI restoration decreases)	ND	HiPCO (9,2) at 1136 nm	ND	Within minutes	No
**Glucose^[68]^**	In solution	Screening approach: Boronic acid derivatives complexed with SC‐SWCNTs Identified derivatives: 4‐cyanophenylboronic acid, 4‐chlorophenylboronic acid Blue‐shift and FI increase	5–30 mm	ND	CoMoCAT (6,5)	ND	Within seconds	Yes
**Glucose, fructose^[81]^**	In solution	SWCNTs locally functionalized with a phenylboronic acid group Blue‐shift and FI decrease	13.5 nm shift with 8.8 mm fructose/19 mm glucose	ND	CoMoCAT E_11_^*^ of (6,5) at 1138 nm	ND	ND	ND
**Glucose^[161]^**	In PBS, human serum, incorporated into membrane device	Rational approach: GOX‐SWCNTs FI increase	In PBS: 3 mm—30 mm tested In serum: 3.3 mm–7.8 mm tested	Different FI increase to mannose, galactose, xylose Over maltose, fructose	CoMoCAT (6,5) at 995 nm	ND	Without/with membrane setup saturation after 1/12–15 min	Yes
**Glycan^[107]^** **Free biotinylated glycans, glycans tethered to streptavidin** **Explored glycan‐lectin pairs: fucose to PA‐IIL lectins and** ***N*** **‐acetylglucosamine to GafD lectins**	In HG	Rational approach: Chitosan‐SWCNTs noncovalently modified with lectins using a Ni^2+^‐NTA/His‐tagged protein mechanism for divalent ion based proximity quenching FI increase	LOD 2 μg/100 ng of glycosylated protein/free glycan to 20 μg of lectin	Greater response of known high affinity pairs than to cross reactions	CoMoCAT (6,5)	Single sensor	Within seconds	Yes
**Glycoforms with high mannose content^[209]^**	In PBS, metabolically induced hypermannosylation of human IgG from CHO cells, SWCNTs in HG	Rational approach: Chitosan‐SWCNTs noncovalently modified with PSA‐lectin using a Ni^2+^‐NTA/His‐tagged protein conjugation for divalent ion based proximity quenching FI increase	Dissociation constant 1.3–55 μm	Over human/mouse IgG with absent mannose content	HiPCO	ND	Within 5 min	ND
**Saccharide^[210]^**	In PBS	Semirational approach: Phenylboronic acid (PBA) grafted, polyethylene glycol 8‐membered branched polymers (PPEG8) wrapped SWCNTs Three PBAs to measure saccharide binding: 4‐carboxyphenylboronic acid, *N*‐(4‐phenylboronic)‐ succinamic acid, 3‐carboxy‐5‐nitrophenylboronic acid FI quenching	FI changes varied for different saccharide, e.g. 4‐carboxyphenylboronic acid‐PPEG8‐SWCNTs respond with −20 % FI change upon 10 mm ribose and 15 % upon arabinose	Not selective over DA Most responsive to pentoses, such as arabinose, ribose, and xylose	Chirality mix (9,4)	ND	1 h incubation of analytes	ND
**Saccharide^[211]^**	In DMSO/PBS	Screening approach: Polymer or surfactant SWCNTs modified with noncovalent adsorption of PBAs Saccharide responses occur only within corona phases that have allowed PBAs to adsorb and prequench SWCNT emission (e.g. most PBAs fail to penetrate SDBS corona) Recognition upon saccharide adsorption into SWCNT corona, followed by surface reaction of PBA	mm range tested	Corona‐phase environment has a profound effect on selectivity of saccharide binding	Chirality mix	ND	Within minutes	ND

[a] FI: fluorescence intensity, ND: ‐not determined, HG: hydrogel, **: limited by the Abbe limit, diffusion and detection speed.

**Table 7:** Detailed overview of fluorescent SWCNT based sensors for DNA/RNA.^[a]^

**Target**	**Biological system**	**Recognition strategy**	**Sensitivity**	**Selectivity**	**SWCNT chirality/ wavelength**	**Spatial resolution**	**Temporal resolution**	**Reversibility**
**DNA hybridization^[212]^**	In Tris buffer	Rational approach: ssDNA‐SWCNTs Hybridization of 24‐mer oligonucleotide sequence with its complement causes blue‐shift	LOD 6 nm	Over noncomplementary DNA	HiPCO (6,5)	ND	13 h to reach steady state (for 625 nm DNA)	ND
**MicroRNA/DNA hybridization^[163]^**	In urine, serum, implanted in live mice by using a semipermeable membrane capillary	Rational approach: SWCNTs functionalized with 23‐mer microRNA capture sequence (complementary to target oligonucleotide) and (GT)_15_ (for SWCNT colloidal stability) Low concentrations of SDBS (5.7 mm) cause an increased blue‐shift (and FI enhancement) by an order of magnitude	In buffer: 10 pm–1 μm In urine: LOD 1 nm In serum: similar, but addition of proteinase K necessary for detection of RNA (to deactivate RNase, which can degrade RNA sequence) In vivo: LOD 100 pm	Over random 23‐mer sequences and similar sequences	Chirality mix	Single sensor	Within 10 min/120 min in buffer/in vivo Kinetics are 1.8 × faster for DNA vs. RNA	Yes, by toe‐hold‐mediated strand displacement, but slower than detection in forward direction
**Single nucleotide polymorphism (SNP)^[213]^**	In Tris buffer	Rational approach: TAG CTA TGG AAT TCC TCG TAG GCA ‐SWCNTs Blue‐shift upon SNP detection Not all ssDNA sequences can be used for SNP detection, surface coverage of SWCNTs with ssDNA shouldn't be too high	0.8 meV blue‐shift	Distinction from response caused by complementary DNA due to higher energy shift of 1.2 meV	HiPCO (6,5)	ND	8 h to reach steady state	ND
**ssRNA genome of intact HIV^[164]^**	In PBS, FBS	Rational approach: (GT)_15_(T)_15_‐SWCNTs SDS for blocking nonspecific binding in serum and denaturation of virus RNA genome hybridizes to sensor, freeing space on the SWCNT surface, denatured viral proteins bind to the surface, eliciting an enhanced blue‐shift	3–9 nm blue‐shift depending on chirality	Control DNA causes slight red‐shift	HiPCO (8,6)	ND	Wavelength shift within 20 min (after 180 min 3 nm shift)	ND

[a] FI: fluorescence intensity, ND: not determined.

**Table 8:** Detailed overview of fluorescent SWCNT based sensors for enzymes.^[a]^

**Target**	**Biological system**	**Recognition strategy**	**Sensitivity**	**Selectivity**	**SWCNT chirality/ wavelength**	**Spatial resolution**	**Temporal resolution**	**Reversibility**
**Enzyme activity^[166]^**	In solution	BSA‐SWCNTs for bacterial protease, citrus pectin‐SWCNTs for pectinase, carboxymethylcellulose (CMC)‐SWCNTs for cellulase Target enzyme degrades the polymer‐wrapping, resulting in FI quenching	LOD 5 fm Sensitivity and repeatability of sensors varies due to affinity of polymers to the SWCNT	ND	CoMoCAT, (6,5) at 975 nm	ND	Real time Signal stabilized 1–3 h after incubation (24–29 h for 5 fm)	No
**Enzyme activity^[167]^**	In soil, diluted with DI water	Albumin‐SWCNTs for proteolytic activity, lignosulfonic acid (LSA)‐SWCNTs for lignin‐modifying activity, CMC‐SWCNTs for cellulolytic activity Target enzyme degrades the polymer‐wrapping resulting in FI quenching or target enzyme further protects the SWCNT resulting in FI increase	ND	LSA‐SWCNTs respond to pH changes CMC‐SWCNTs to nonspecific binding of proteins	Chirality mix, (6,5)	ND		ND
**Myeloperoxidase (MPO)^[169]^**	MPO/H_2_O_2_/Cl^−^ system	GO‐SC‐SWCNTs Increasing FI at 420/430 nm due to oxidation and degradation of GO Decreasing NIR SWCNT FI due to generation of defects on the SWCNT surface	Linear response to MPO‐catalyzed degradation	ND	CoMoCAT, 998 nm GO: 420 nm	ND	Real time, within 5 days	No
**Trypsin^[168]^**	SWCNTs drop‐casted and dried on glass fiber paper, fixed on plastic strips Urine samples diluted in PBS	Screening approach: Peptide‐SWCNTs (HexCoil‐Ala) Target enzyme degrades the peptide‐wrapping resulting in FI quenching	1–20 μg mL^−1^ Urine samples: reduced sensitivity	8.3 %/33 % urine (without trypsin) caused 33 %/50 % FI decrease over urea	CoMoCAT, (6,5)	ND	Significant FI decrease after 2 h/1 h of incubation at concentrations of 1/5–20 μg mL^−1^	No

[a] FI: fluorescence intensity, ND: not determined.

**Table 9:** Detailed overview of fluorescent SWCNT based sensors for epitopes and metabolites from pathogens.^[a]^

**Target**	**Biological system**	**Recognition strategy**	**Sensitivity**	**Selectivity**	**SWCNT chirality/ wavelength**	**Spatial resolution**	**Temporal resolution**	**Reversibility**
**Distinction between F’‐positive and F’‐negative bacterial strains, specific F’‐negative bacteria^[170]^**	In tissue phantom, mice, SWCNTs injected intravenously	Rational approach: For distinction: M13 bacteriophage‐SWCNTs F’‐negative bacteria: Anti‐bacterial antibody‐SWCNTs via streptavidin‐biotin reaction	F’‐negative bacteria: Detection of *S. aureus* intramuscular infections with 3400 % FI enhancement	Over control (injection of PBS)	HiPCO	μm	Imaging of mice 8–48 h after injection	ND
**Distinction between pathogens**: ***S. aureus, S. epidermidis, S. pyogenes, E. faecalis, E. coli, P. aeruginosa*** ^**[29]**^	SWCNTs in HG array with low porosity for small molecules (e.g. siderophores) and high porosity for large enzymes, bacteria plated on top, in human synovial fluid, tissue phantom	Spatially encoding, distinction with PCA: Sensors for specific bacterial targets (rational approach): 1) Lipopolysaccharides (LPS) sensor: LPS‐binding peptide linked to NH_2_‐(GT)_10_‐SWCNTs, FI increase 2) Siderophore sensor: hemin‐binding aptamer‐ (HeApta)SWCNTs, HeApta binds to hemin, which brings Fe^3+^ into proximity of the SWCNT and quenches it. Siderophore can reverse this effect by removing iron, which increases FI again 3) Nuclease activity sensor: calf‐thymus‐(CT‐)DNA‐SWCNTs, report to DNase I and *S. aureus* nuclease activity due to degradation of CT‐DNA via FI modulation 4) Protease sensor: BSA‐SWCNTs, FI decrease in response to protease Generic lower‐selectivity sensors to increase the discrimination power of the sensor array: (GT)_10_‐, C_30_‐, (GC)_15_‐, PEG‐PL‐SWCNTs react to changes in pH, oxygen or protein concentration For spectrally encoding: LPS‐(6,5)‐SWCNTs, PEG‐PL‐(9,4),(8,6),(9,5)‐SWCNTs and EB‐NS reference	LPS sensor: up to 12.5 μm Siderophore sensor: up to 10 μm LOD in >7 mm depth tissue	Distinction of *P. aeruginosa*, *S. aureus*, *S. epidermidis*, *E. coli* and of 43 different clinical isolates of *S. aureus* and *S. epidermidis* with 80% likelihood *E. faecalis* and *S. pyogenes* not distinguishable Over human synovial fluid LPS sensor: responds to different LPS structures with different sensitivity, siderophore sensor: not responsive to weaker chelators such as ethylenediaminetetraacetic acid citrate	Spatial encoding: CoMoCAT, >900 nm Spectral encoding: EB‐NS: 920 nm, (6,5): 920 nm, (9,4), (8,6), (9,5) > 1110 nm	mm for a stand‐off distance of ≥25 cm	Limited by diffusion of analytes through HG Within 24–72 h distinction possible	ND

[a] FI: fluorescence intensity, ND: not determined, HG: hydrogel.

## Conflict of interest

The authors declare no conflict of interest.

## Biographical Information

*Julia Ackermann received her M.Sc. in nanoengineering with a focus on nano(opto)electronics at the university of Duisburg‐Essen (Germany). Since 2020 she has been persuing her Ph.D. in the technology division of biomedical nanosensors of the Fraunhofer Institute for Microelectronic Circuits and Systems in Duisburg (Germany). Her work focuses on improving the overall selectivity of carbon nanotube based sensors by pattern recognition*.

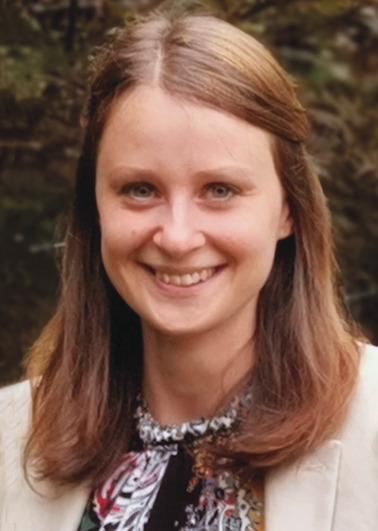

## Biographical Information

*Justus Metternich received his Bachelor's degree in biotechnology from the University of Applied Sciences Darmstadt. After a short stay at the Centro de Investigaciones Biológicas Margarita Salas in 2018, he continued with his Master's studies in chemistry at Uppsala University. Since November 2020, he has been part of the Attract group of Sebastian Kruss and pursuing his Ph.D. at Ruhr‐University Bochum. His research focuses on the design of fluorescent carbon nanotube functionalizations for the detection of pathogens*.

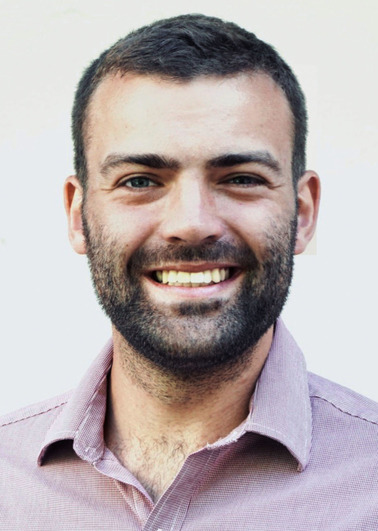

## Biographical Information

*Svenja Herbertz received her Ph.D. in Physics at the Solid‐State Physics Laboratory at the Heinrich‐Heine‐University in Düsseldorf (Germany) in 2019. During her years of study in Medical Physics she gained experience in the field of optical spectroscopy and use of semiconductor quantum dots for fluorescence labeling in biomedical applications. She is currently a researcher at the Fraunhofer Institute for Microelectronic Circuits and Systems in Duisburg (Germany) in the technology division of biomedical nanosensors*.

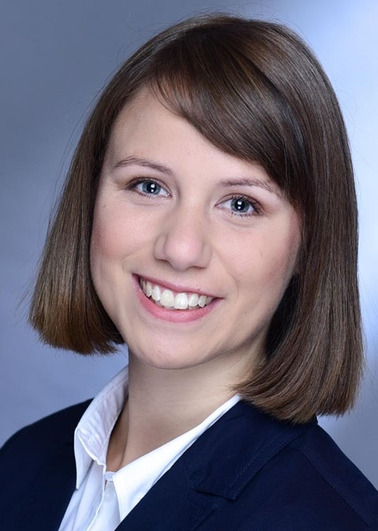

## Biographical Information

*Sebastian Kruss received his Ph.D. in biophysical chemistry at Heidelberg University and the Max Planck Institute for Intelligent Systems (with Prof. Joachim Spatz). He then moved to the Massachusetts Institute of Technology (with Prof. Michael Strano), where he worked on carbon nanomaterials. After heading an independent research group at Göttingen University (2015–2020), he became professor of physical chemistry at Ruhr‐Universität Bochum and Attract group leader at Fraunhofer IMS. His research focuses on novel materials, fluorescence spectroscopy and microscopy, biosensors, and cell biophysics*.